# *Gb3* encodes a unique kinase fusion protein conferring greenbug resistance in wheat

**DOI:** 10.1038/s41588-026-02754-0

**Published:** 2026-09-23

**Authors:** Dhondup Lhamo, Jianqiang Shen, Genqiao Li, Toni Mohr, Roger Thilmony, Prakitchai Chotewutmontri, Han-Chang Chang, Honglian Ye, Mingcheng Luo, Shuyu Liu, Guihua Bai, Jackie Rudd, Brett F. Carver, Yong Q. Gu, Xiangyang Xu, Steven S. Xu

**Affiliations:** 1 US Department of Agriculture—Agricultural Research Service, Crop Improvement and Genetics Research Unit, Western Regional Research Center, Albany, CA USA; 2 Department of Plant and Microbial Biology, University of California, Berkeley, CA USA; 3 US Department of Agriculture—Agricultural Research Service, Peanut and Small Grains Research Unit, Stillwater, OK USA; 4 Department of Plant Sciences, University of California, Davis, CA USA; 5 Department of Soil and Crop Science, Texas A&M University, College Station, TX USA; 6 Department of Agronomy, Kansas State University, Manhattan, KS USA; 7 Texas A&M AgriLife Research, High Plains Research and Extension Center, Canyon, TX USA; 8 Department of Plant and Soil Sciences, Oklahoma State University, Stillwater, OK USA

**Keywords:** Gene expression, Genome-wide association studies, Mutagenesis, Metabolomics, Transcriptomics

## Abstract

Understanding plant resistance to insects has lagged owing to a scarcity of cloned resistance genes. Here we report cloning of the greenbug resistance gene *Gb3* in wheat (*Triticum aestivum* L.), initially introgressed into cultivars such as ‘TAM112’ from *Aegilops tauschii*. *Gb3* encodes an intracellular kinase–pseudokinase protein, featuring a unique MAP3K-like domain. This gene probably originated through fusion, duplication and indel events in the Triticeae tribe, retaining its functionality in several *Aegilops* species. Transgenic *Gb3* expression in the susceptible Fielder wheat enhanced resistance to multiple biotypes, while the *Gb3* knockout in TAM112 resulted in susceptibility. Comprehensive analyses indicate that greenbugs preferentially feed on susceptible plants, inducing a prolonged oxidative stress response. Conversely, resistant plants expressing *Gb3* mediate greenbug resistance by producing oxylipins and other defensive compounds. Our findings lay the groundwork for investigating plant–insect interactions and provide a crucial genetic resource for developing sustainable aphid resistance in cereal crops.

## Abstract (web summary)

*Gb3* encodes an intracellular kinase–pseudokinase protein with a unique MAP3K-like domain that confers resistance to multiple greenbug biotypes in wheat.

## Main

Global wheat yields suffer major losses from both diseases and insect pests, with invasive insects alone accounting for at least US$70 billion in crop production losses annually^1^. Over the past three decades, more than 450 resistance (R) genes have been cloned in various crops^2^. Approximately 70 of these R genes have been documented within the Triticeae tribe, predominantly encoding canonical nucleotide-binding leucine-rich repeat (NLR) proteins^3^. Recently, kinase fusion proteins (KFPs) have emerged as an important class of noncanonical R genes, with 19 reported so far for fungal pathogens; among these, 11 encode tandem kinase (TK) proteins characterized by dual kinase domains^3,4^. These cloned R genes for TKs include *Yr15* (*WTK1*) for stripe rust^5^; *Rpg1* (ref. ^6^), *Sr60* (*WTK2*)^7^ and *Sr62* (*WTK5*)^8^ for stem rust; *Pm24* (*WTK3*)^9^, *WTK4* (ref. ^10^), *Pm57* (*WTK6b-vWA*)^11^ and *Pm36* (*WTK7-TM*)^12^ for powdery mildew; *Lr9* (*Lr58*/*WTK6-vWA*)^13^ and *Lr39* (*WTK-MSP-WD40*)^14^ for leaf rust; and *Rwt4* (ref. ^15^) for wheat blast. The cloning and functional characterization of these R genes have substantially enhanced our understanding of their roles as intracellular immunoreceptors that activate plant immunity^16–19^.

By contrast, the progress in the cloning of insect R genes in wheat has been markedly slower, with only one R gene identified in wheat and a total of ten across all plant species^20–29^. This disparity arises from the complexities of resistance phenotyping and the unpredictable nature of insect R gene expression in plants. Consequently, cloning insect R genes and resolving their molecular mechanisms remain challenging, impeding the development of crop cultivars with durable resistance.

Greenbug, *Schizaphis graminum* (Rondani), is a major nonhost-altering aphid of 70 Poaceae species and a vector of the devastating barley yellow dwarf virus^30^. Since its initial detection in 1882, the greenbug has remained a major threat to wheat, sorghum and barley production in the Great Plains of the USA, causing US$100 million in losses during severe outbreaks^31^. It consists of highly diverse populations of about 50 biotypes identified in the USA (Supplementary Table 1), with biotypes E and I being the most prevalent^32^. The continuous occurrence of new biotypes often compromises crop resistance and has prompted the discovery of several greenbug R (*Gb*) genes, each exhibiting distinct resistance profiles (Supplementary Table 1). However, none of these genes have been cloned since the discovery of *Gb1* in the 1950s^33^. Six *Gb* genes (*Gb3*, *Gb4*, *Gb7*, *Gb8*, *Gb9* and *Gb49921*) have been identified on chromosome 7D of *Ae. tauschii*. Of these, *Gb3* holds historic significance, having served for more than 40 years in US breeding programs as the only *Gb* gene widely deployed in bread wheat cultivars (TAM110, TAM112 and TAM204) without associated fitness costs^34–37^. Remarkably, *Gb3* provides resistance to 26 of the 47 previously tested greenbug biotypes (Supplementary Table 1).

This study utilized association mapping of an *Ae. tauschii* panel to map and clone *Gb3*, validating its resistance function against multiple biotypes through complemented and CRISPR–Cas9 knockout transgenics. Our analyses of protein and domain sequences, subcellular localization and in vitro kinase assays demonstrated that *Gb3* encodes a unique intracellular KFP with a kinase–pseudokinase configuration. Haplotype and evolutionary studies showed that *Gb3* evolved specifically within the Triticeae tribe, with nonfunctional mutations rarely observed in the panel. By using newly developed markers and nanopore sequencing, we consolidated six 7D *Gb* genes that encode the same KFP and identified functional allelic variants in other *Aegilops* species. In addition, we carried out histological, transcriptomic and metabolomic analyses to explore the mechanism of *Gb3*-mediated resistance.

## Results

### *Gb* gene identification using association mapping

**Fig. 1:**
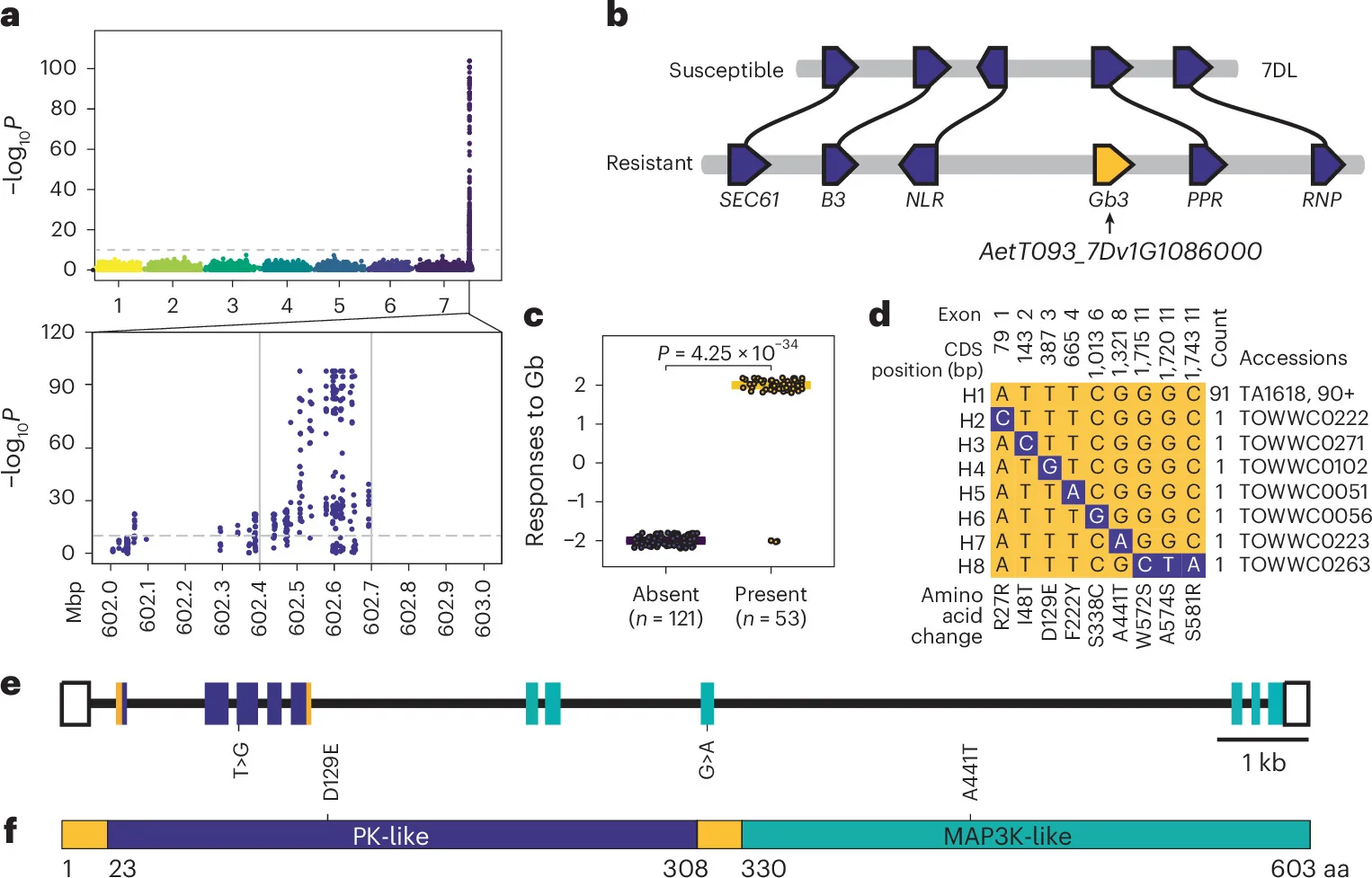
*Gb3* encodes a KFP. **a**, A genome-wide association study delineated the *Gb3* locus to a 300-kb interval (marked by gray vertical lines) on chromosome arm 7DL of AL8/78. The significance threshold (dashed gray line) was set at −log_10_*P* ≥ 10. **b**, Collinearity analysis of the *Gb3* locus using resistant and susceptible *Ae. tauschii* pangenome sequences. *Gb3* is represented in yellow, with gene annotation based on the *Ae. tauschii* reference genome T093. **c**, A box plot illustrating the presence and absence of *Gb3* in *Ae. tauschii* accessions resistant (score of 2) and susceptible (score of −2) to all four greenbug biotypes. An unpaired two-tailed *t*-test was performed to assess statistical significance, with the *P* value indicated. **d**, Natural variations in the CDS of *Gb3* in the *Ae. tauschii* panel, consisting of 260 accessions. H1–H8 represent eight different haplotypes, and aa indicates amino acid substitutions. **e**,**f**, Schematic representations of the predicted *Gb3* gene structure (**e**) and protein domains (**f**). Exons are designated by colored boxes, 5′ and 3′ UTRs by white boxes and introns by black lines. Purple and cyan indicate kinase domains, and yellow indicates flexible linkers. Natural mutations in nucleotides (**e**) that lead to amino acid changes (**f**) are shown.

**Extended Data Fig. 1:**
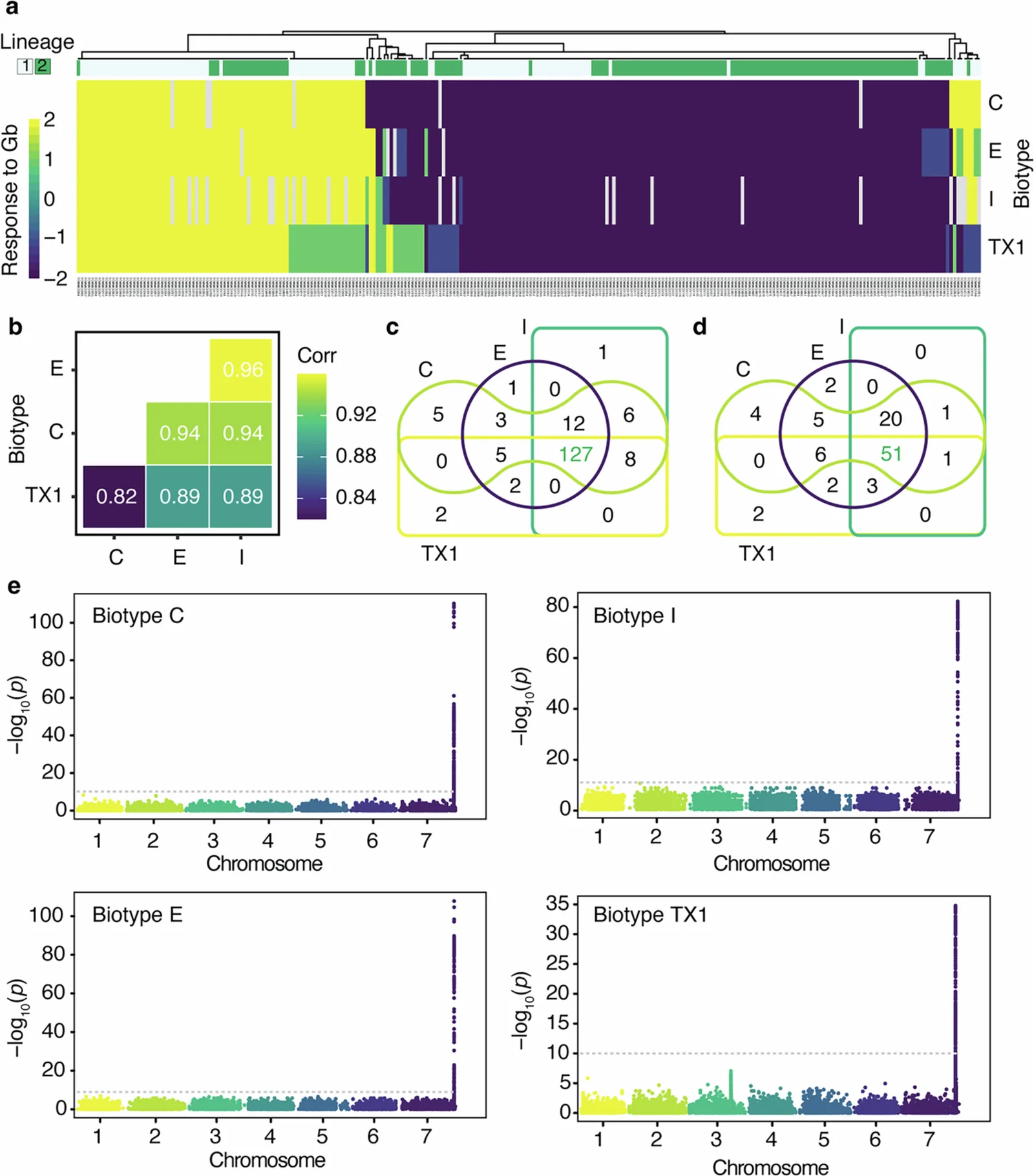
Response of the *Ae. tauschii* panel to different greenbug biotypes. **a**, Heatmap displaying the phenotypic screening of the panel against biotypes C, E, I, and TX1. Responses to greenbug are transformed into resistant = 2, moderately resistant = 1, moderately susceptible = -1, and susceptible = -2. White in the heatmap represents unavailable data. Lineages of accessions are shown on top in light and dark green boxes. **b**, Correlation between the panel’s response to four biotypes**. c, d**, Overlap in the number of *Ae. tauschii* accessions susceptible (**c**) and resistant (**d**) to four biotypes. Values in green represent overlapping accessions in responses to all four biotypes. **e**, Association mapping using the panel’s response to individual biotypes. The significance threshold (gray dashed line) was set at -log_10_(*P*)≥10.

To clone the *Gb* gene from *Ae. tauschii*, we utilized a panel of 260 accessions, previously genotyped^10^, and evaluated for resistance to four greenbug biotypes C, E, I and TX1 (ref. ^38^) (Extended Data Fig. 1a and Supplementary Table 2). Strong correlations (*r* > 0.8) were observed among biotypes, with 19.6% and 48.5% of accessions exhibiting resistance and susceptibility to all four biotypes, respectively (Extended Data Fig. 1b–d). Using these accessions, we performed genome-wide association mapping and delineated the R gene to a 300-kb interval between 602.4 Mbp and 602.7 Mbp on chromosome arm 7DL of the AL8/78 reference genome^39^ (Fig. 1a and Extended Data Fig. 1e). This interval overlapped the previously defined biparental mapping interval of *Gb3* (ref. ^40^), containing five and six annotated genes in the reference genomes AL8/78 and T093, respectively (Fig. 1b and Supplementary Table 3).

**Extended Data Fig. 2:**
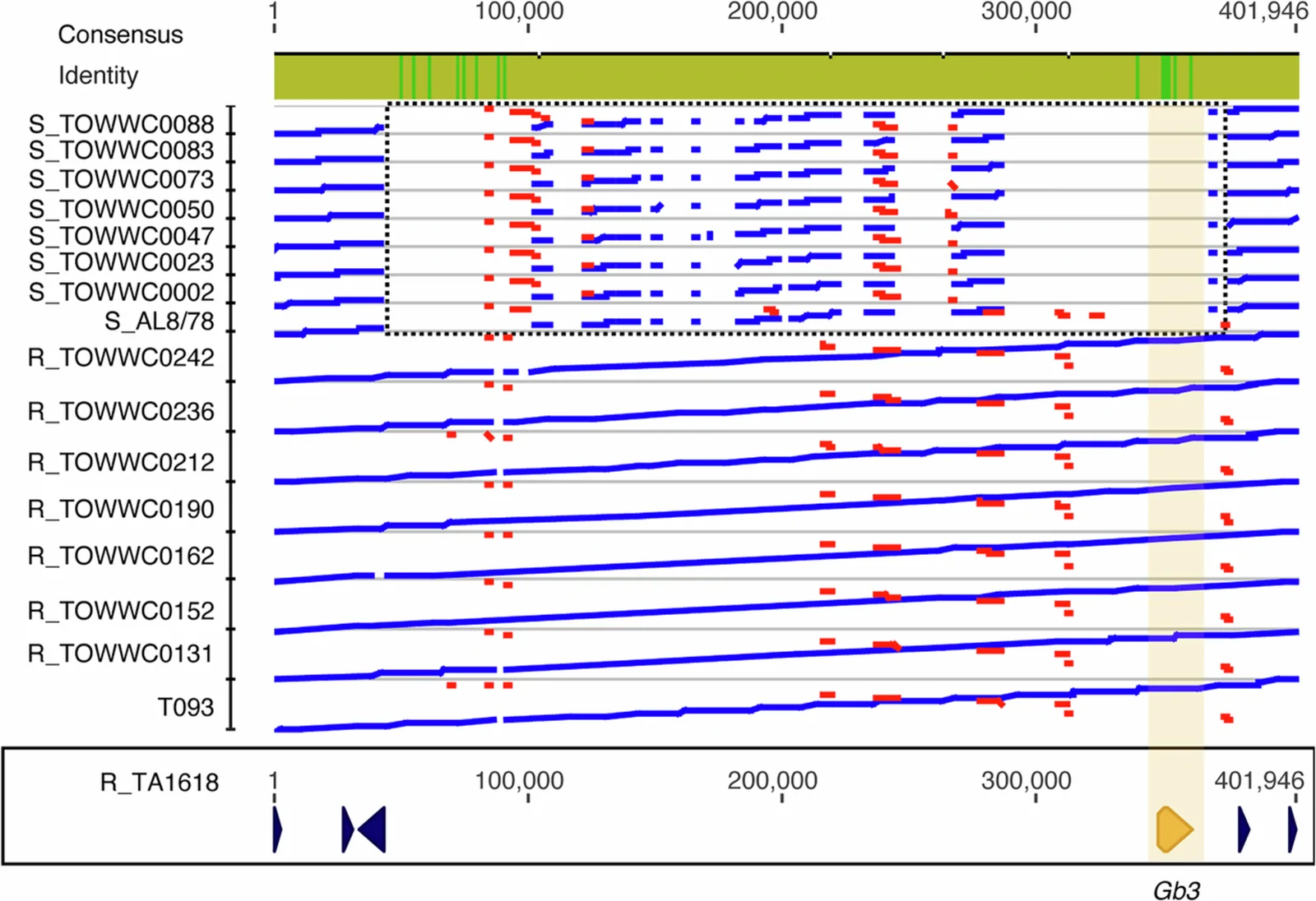
Collinearity of the 300-kb interval among the eight susceptible and resistant *Ae. tauschii* pangenome accessions. Forward (blue line) and reverse (red line) sequences of pangenome accessions are aligned to the resistant *Gb3* donor, TA1618, by the LASTZ alignment. The AL8/78 and T093 reference genomes are also aligned. High-confidence gene annotations are based on T093. ‘R’ before the accession ID denotes resistant, while ‘S’ denotes susceptible. The dashed black box indicates the deletion boundaries in susceptible accessions. The yellow vertical box highlights the presence of *Gb3* in resistant pangenome sequences and its absence in susceptible pangenome sequences.

To identify the leading candidate gene, we conducted a collinearity analysis of the 300-kb interval across eight greenbug-susceptible and greenbug-resistant *Ae. tauschii* pangenome accessions^14^ along with AL8/78 and T093 (Extended Data Fig. 2). This analysis revealed extensive and scattered deletions of 155–180 kb within the interval across all susceptible accessions. By contrast, one annotated gene in T093, *AetT093_7Dv1G1086000*, referred to hereon as *Gb3*, was consistently present in all resistant pangenome accessions and completely absent in all susceptible accessions (Fig. 1b and Extended Data Fig. 2).

### *Gb3* haplotype and domain analyses

Evaluation of *Gb3* in the *Ae. tauschii* panel revealed its presence in 100% of the resistant accessions and absence in 98% of the susceptible accessions (Fig. 1c). Among the accessions containing *Gb3*, eight distinct haplotypes (designated H1–H8) were identified, with H1 being the most prevalent, observed in 91 accessions (Fig. 1d). H1 was present in both lineages, predominating in lineage L1 (59%) as opposed to lineage L2 (17%) (Supplementary Table 4). This haplotype was most frequent in Afghanistan (17%) and Turkmenistan (12%) within lineage L1, and in Iran (15%) within lineage L2, suggesting that these regions may be the origin centers of *Gb3*. Furthermore, the H1 haplotype in lineage L1 was widespread across Asia, while H1 in lineage L2 remained confined to West Asia (Iran and Turkmenistan). Instances of mutations leading to susceptibility were infrequent within the natural population; only two accessions carried nonsynonymous mutations in exon 3 (D129E) and exon 8 (A441T), which resulted in susceptibility to all four biotypes (Fig. 1d–f and Supplementary Table 4).

**Extended Data Fig. 3:**
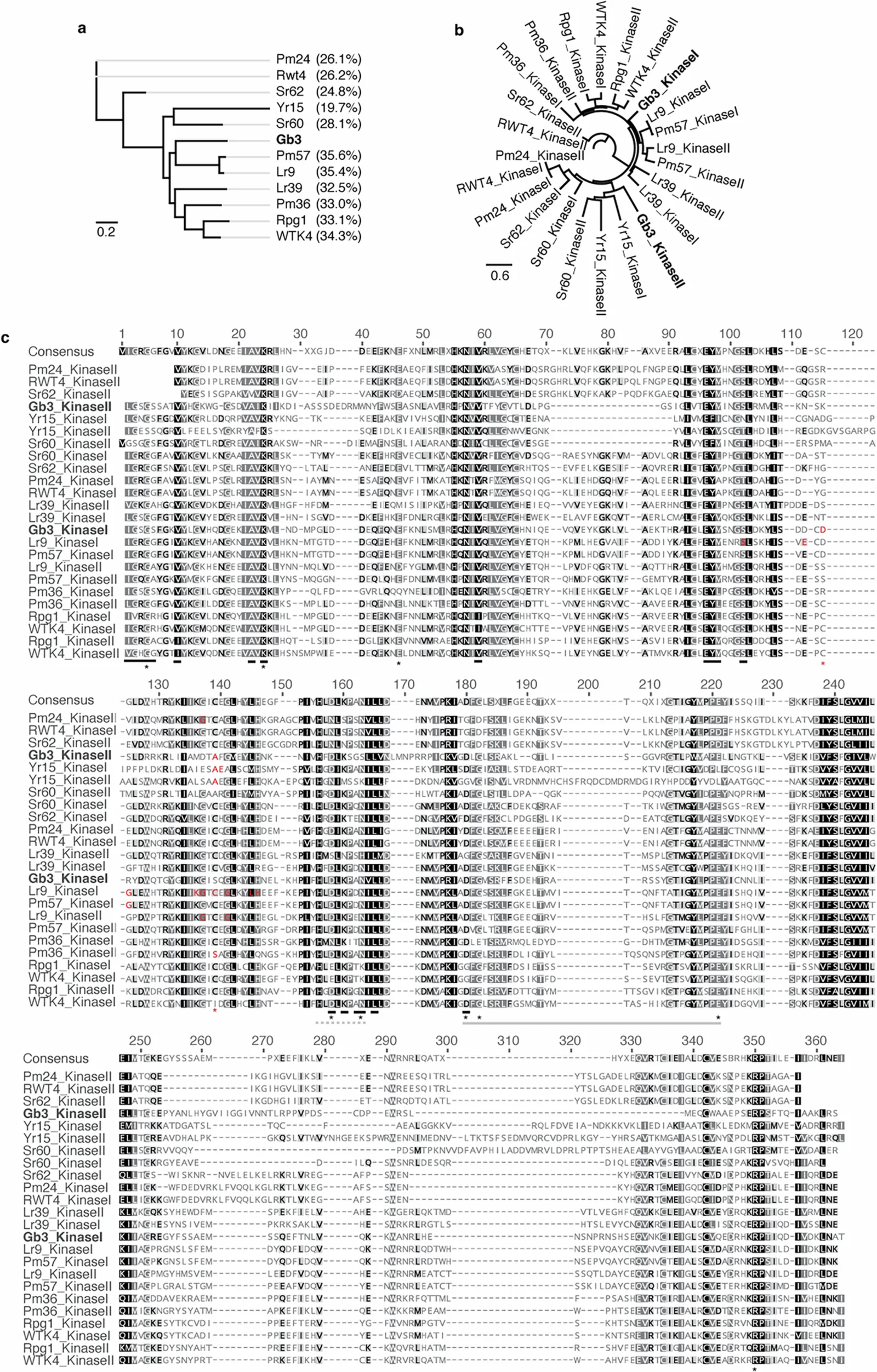
Alignments of protein and domain sequences of Gb3 with 11 cloned tandem kinases. **a**, Phylogenetic relationship of Gb3 with tandem kinases. The percent identities of tandem kinases to Gb3 are presented in brackets. **b** and **c**, Domain analysis of Gb3 and other tandem kinases based on phylogeny (**b**) and conserved amino acid residues (**c**). The phylogenetic trees were generated using the neighbor-joining method with 1000 bootstrap replicates in Geneious Prime. The dual kinase domains were designated Kinase I and Kinase II according to their position in the protein sequence. Nine key conserved kinase residues are marked by black asterisks, ATP binding sites with black underlines, the catalytic loop (non-RD) with gray dashed underlines, and the activation loop with solid gray underlines in (**c**). Mutations resulting in disease susceptibility are presented in red asterisks, and residues marked red in the specified domain.

We analyzed the protein domains of *Gb3* to assess the potential significance of the two natural mutations in *Gb3*-mediated resistance. *Gb3* encodes a 603-amino acid (aa) KFP with two distinct kinase domains: protein kinase (PK)-like and mitogen activated protein kinase kinase kinase (MAP3K)-like (Fig. 1f). Our comparative analysis of the *Gb3* protein sequences against 11 cloned TKs revealed notable divergence, with only 20–36% identity (Extended Data Fig. 3a). The examination of kinase domains indicated that the PK-like domain of *Gb3* clustered with *Lr9* and *Pm57*, while the MAP3K-like domain aligned with *Yr15* and *Sr60* (Extended Data Fig. 3b). We also compared the two mutations (D129E and A441T) of *Ae. tauschii* to ethyl methanesulfonate (EMS)-induced mutations in the cloned TKs around the same region (Extended Data Fig. 3c). We found that the aspartate in D129 of *Gb3* is present in kinase I of *Lr9* and *Pm57*, and the alanine in A441 of *Gb3* is conserved within both kinase domains of *Yr15* and *Sr60*. These two amino acids are situated 15 residues apart, slightly upstream of the catalytic loop. Furthermore, substitutions at amino acids adjacent or near the conserved aspartate in kinase I of *Lr9* and *Pm57*, as well as an analogous alanine-to-threonine substitution in both kinase domains of *Yr15* contributed to disease susceptibility. These findings emphasize the importance of these residues in conferring resistance to both fungal pathogens and insects.

### *Gb3* functional confirmation

**Fig. 2:**
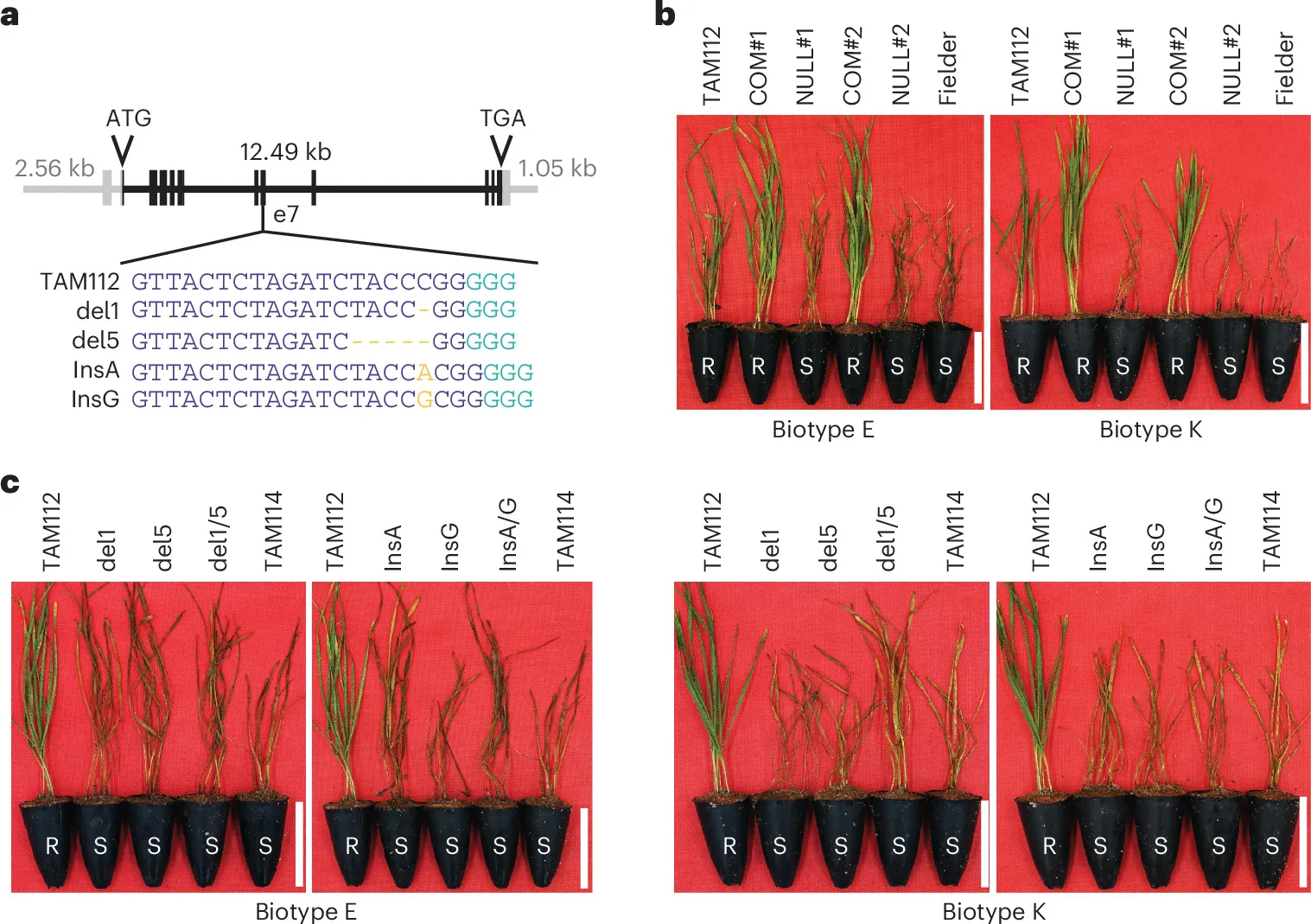
*Gb3* functional confirmation. **a**, Schematic diagrams show the 16.1-kb *Gb3* genomic fragment used for complementation in wheat cv. Fielder (*Gb3*^−^) and the CRISPR-edited alleles generated for *Gb3* knockouts in wheat cv. TAM112 (*Gb3*^+^). The TAM112 sequence of the gRNA on exon 7 (e7) is presented in purple, the protospacer-adjacent motif (PAM) sequence in cyan and the indel edits in yellow. **b**, Representative seedlings of two stable T_2_ homozygous *Gb3*-complemented lines, along with their null and nontransgenic controls, were infested with greenbug biotypes E and K. **c**, T_2_ homozygotic and biallelic indel events of CRISPR–Cas9-mediated *Gb3*-knockout lines, together with their unedited resistant TAM112 and susceptible TAM114 controls, were infested with greenbug biotypes E and K. The indel events were compared against the same controls for the specified biotype. Resistant plants are designated as ‘R’ and susceptible plants as ‘S’. Scale bars, 5 cm (**b**,**c**). Other biotype screenings are shown in Extended Data Figs. 4 and 6.

**Extended Data Fig. 4:**
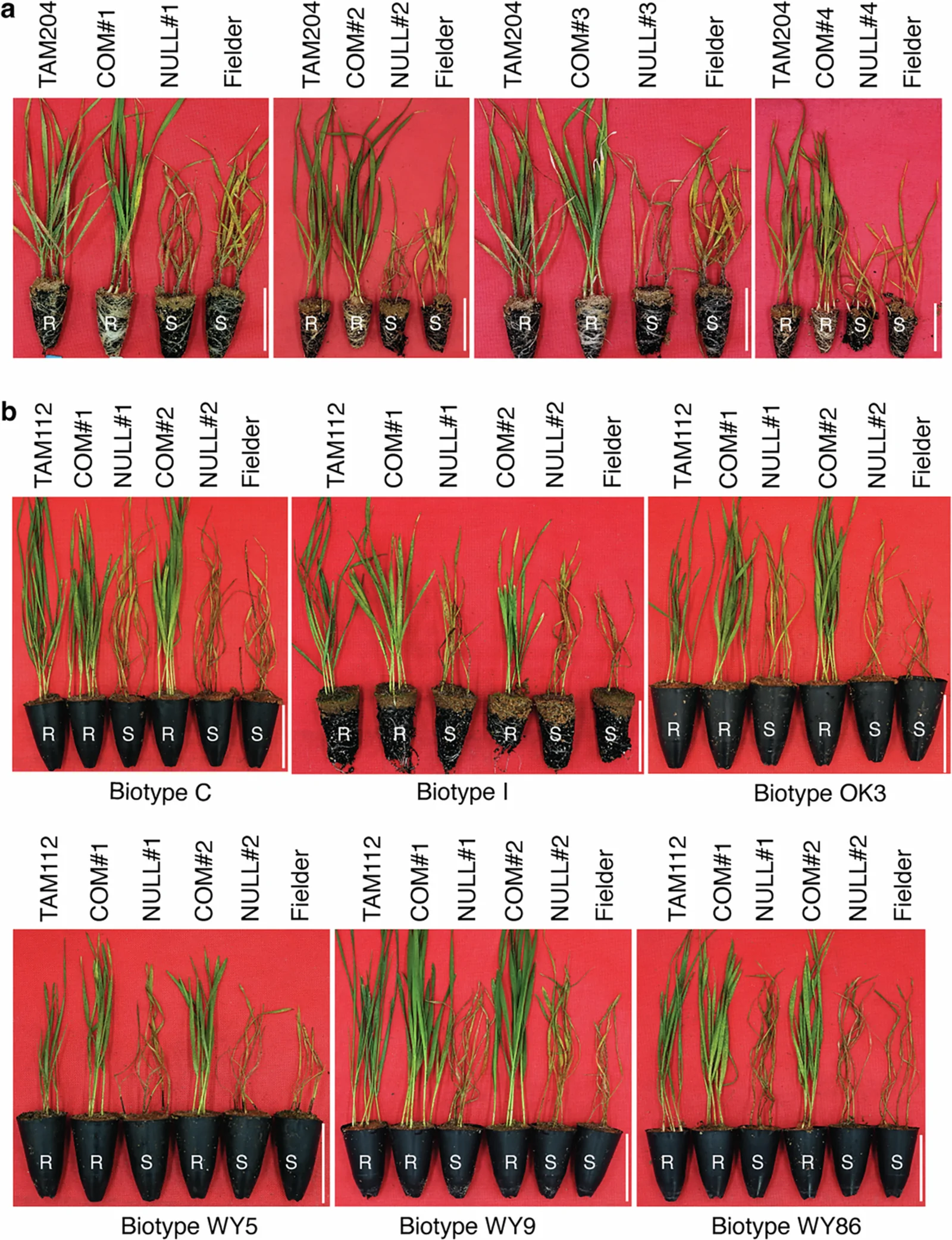
Phenotypic screening of *Gb3-*complemented (COM) lines. **a**, Four independent *Gb3-*complemented T_1_ transgenic lines and their null segregants, along with non-transgenic Fielder (*Gb3*-) and TAM204 (*Gb3*+) controls, were infested with greenbug biotype E. COM#1 and COM#2 have single copy transgene insertions, while COM#3 and COM#4 have 2 and 5 transgene copies, respectively, in the T_0_ generation. The phenotypes for COM#1 and COM#3 were evaluated concurrently with the same set of controls; COM#2 and COM#4 were evaluated in the same manner. **b**, T_2_ COM#1 and COM#2 and their null lines, along with Fielder (*Gb3*-) and TAM112 (*Gb3*+) controls, were screened against six additional biotypes (C, I, OK3, WY5, WY9, and WY86). R for resistant and S for susceptible. Scale bar = 5 cm.

**Extended Data Fig. 5:**
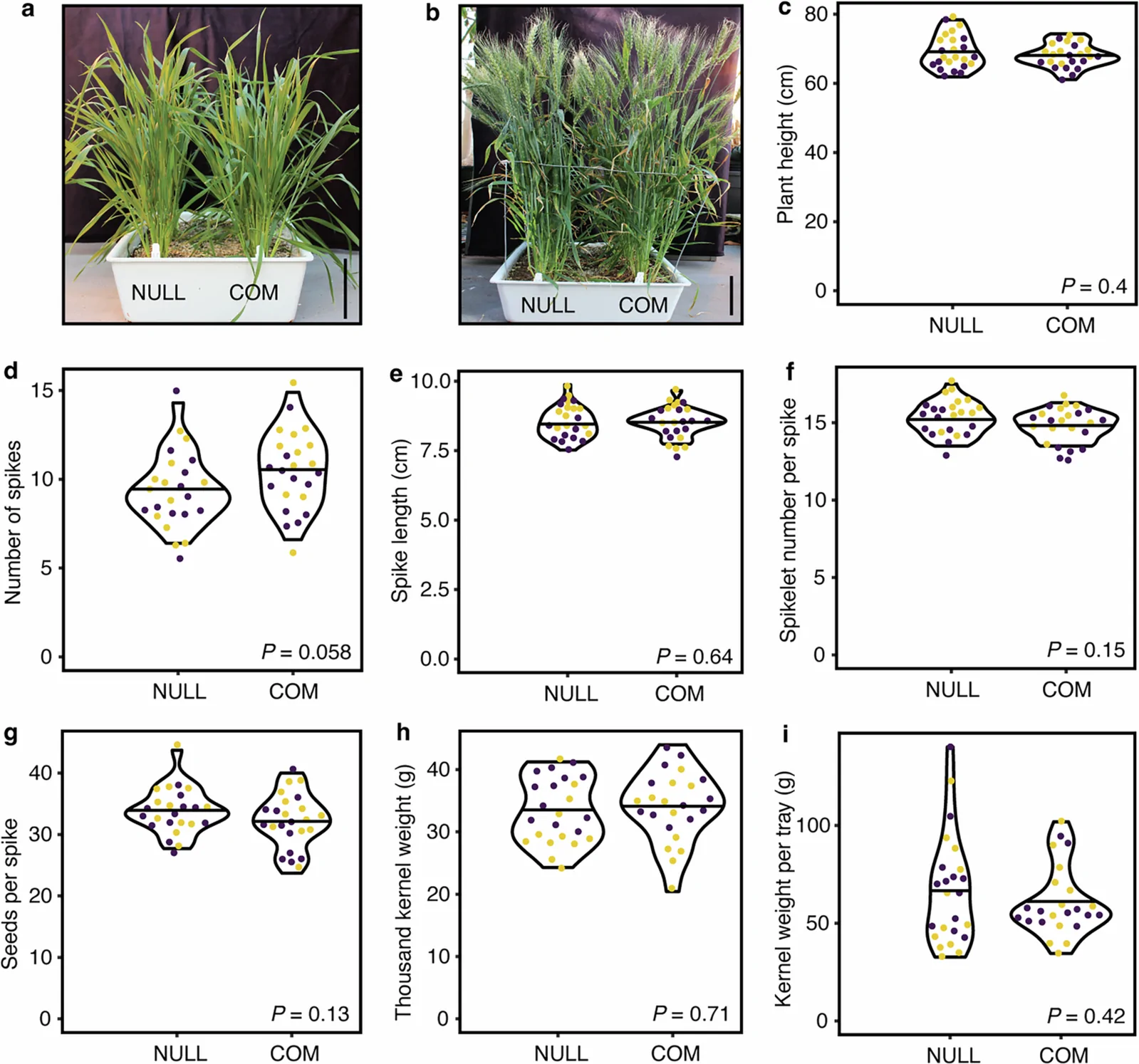
Agronomic traits of null and *Gb3*-complemented (COM) transgenic lines. **a** and **b**, Transgenic plants before (**a**) and after (**b**) heading. Scale bar = 13 cm. **c-i**, Comparison of average plant height (**c**), number of spikes (**d**), spike length (**e**), spikelet number per spike (**f**), seeds per spike (**g**), thousand kernel weight (**h**) and kernel weight per tray (**i**). Each dot represents an average of 8–9 plants per tray with a total of 24 trays. Horizontal lines in the violin plots represent the overall trait means within each genotype across all 24 trays. Statistical significance as indicated by the *P* values were assessed using an unpaired two-tailed *t*-test. Source data

To confirm the role of *Gb3* in greenbug resistance, we cloned the full-length genomic fragment, including its native promoter and terminator, into a binary vector and subsequently introduced the resultant construct into Fielder (Fig. 2a). Four independent T_0_ lines were obtained, with their T_1_ progenies segregating for resistance to the prevalent biotype E (Extended Data Fig. 4a). Two homozygous T_2_-complemented lines were challenged with eight greenbug biotypes collected from diverse hosts and regions across the Great Plains (Supplementary Table 5). As anticipated, Fielder and null transgenic lines remained susceptible, whereas the *Gb3*-complemented lines exhibited resistance to all evaluated biotypes, comparable to the *Gb3* control, TAM112 (Fig. 2b and Extended Data Fig. 4b). In addition, the introduction of *Gb3* in Fielder had no negative impact on the seven evaluated agronomic traits compared to their null controls (Extended Data Fig. 5).

**Extended Data Fig. 6:**
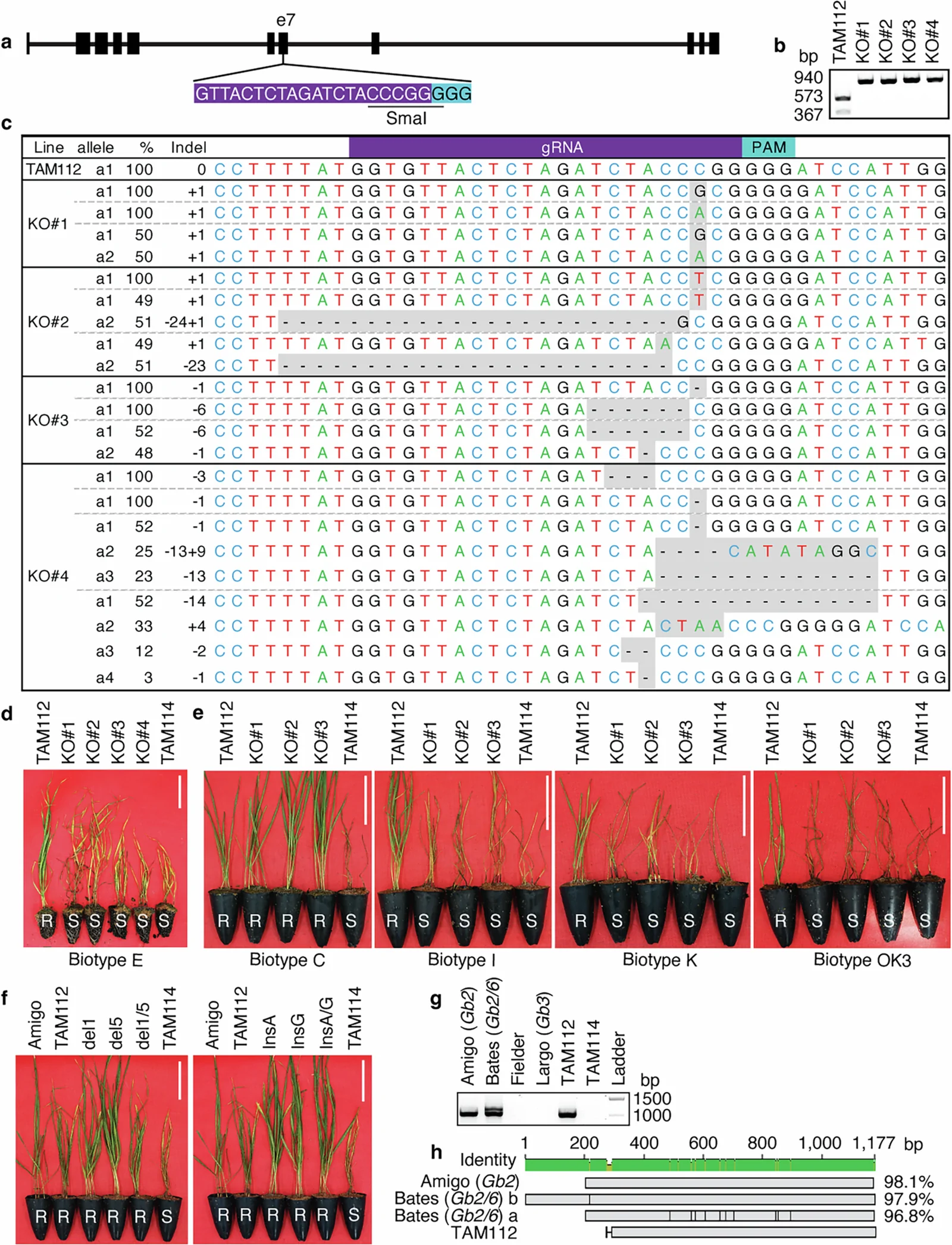
Genotypic and phenotypic evaluation of CRISPR/Cas9-mediated *Gb3*-knockout lines. **a**, Schematic representation of the guide RNA (gRNA, purple highlight) and protospacer-adjacent motif (PAM, cyan highlight) sequences on exon 7 (e7) of the *Gb3* gene. The SmaI recognition site is underlined. **b**, Complete edits of T_0_ knockout lines identified by the Cleaved Amplified Polymorphic Sequences (CAPS) assay using SmaI. **c**, Different CRISPR-edited alleles in the segregating T_1_ population (gray dashed lines) of *Gb3*-knockout lines detected by Sanger sequencing. Indel nucleotides are highlighted in gray. **d**, Initial screening of all four independent T_1_ knockout lines against greenbug biotype E. **e**, Screening of three independent T_1_ knockout lines against four additional biotypes (C, I, K, and OK3). KO#4 was excluded due to seed limitation. **f**, Re-evaluation of biotype C screening in T_2_ knockout lines. The indel events were compared against the same controls. Scale bar = 5 cm. **g, h**, Detection of rye-derived *Gb2* in the TAM112 background using the known EST marker (BE705057) by PCR (**g**) and nanopore sequencing (**h**). Resistant rye cultivar Bates (P1591057) and wheat-rye 1AL·1RS translocation line Amigo were used as *Gb2* positive controls, resistant Largo as the *Gb3* control, and susceptible Fielder and TAM114 as negative controls in (**g**). The gel image was cropped to focus on the expected size of the *Gb2* marker; full-length gel can be found in the Source Data file. The percent identity of TAM112 to the rye *Gb2* marker sequence is presented on the right (**h**). Source data

We also generated *Gb3* knockouts in the TAM112 background using the CRISPR–Cas9 system, producing four independent T_1_ mutant lines (Extended Data Fig. 6a–c). These knockout lines were susceptible to four out of five tested biotypes, in contrast to the unedited *Gb3* in TAM112 (Extended Data Fig. 6d,e). *Gb3* knockout lines remained resistant to biotype C in both T_1_ and T_2_ generations (Extended Data Fig. 6e,f). This residual resistance is explained by the presence of rye-derived *Gb2* in TAM112 (ref. ^35^), which is susceptible to all tested biotypes except for C (Supplementary Table 5). The presence of *Gb2* in TAM112 was confirmed by PCR marker and sequencing (Extended Data Fig. 6g,h). We reconfirmed the greenbug susceptibility of *Gb3*-knockout lines against biotypes E and K in the T_2_ generation harboring different indel events (Fig. 2c). These genetic engineering results confirm the role of the dual kinase in *Gb3*-mediated resistance.

### Gb3 protein structure, localization and kinase activity

**Fig. 3:**
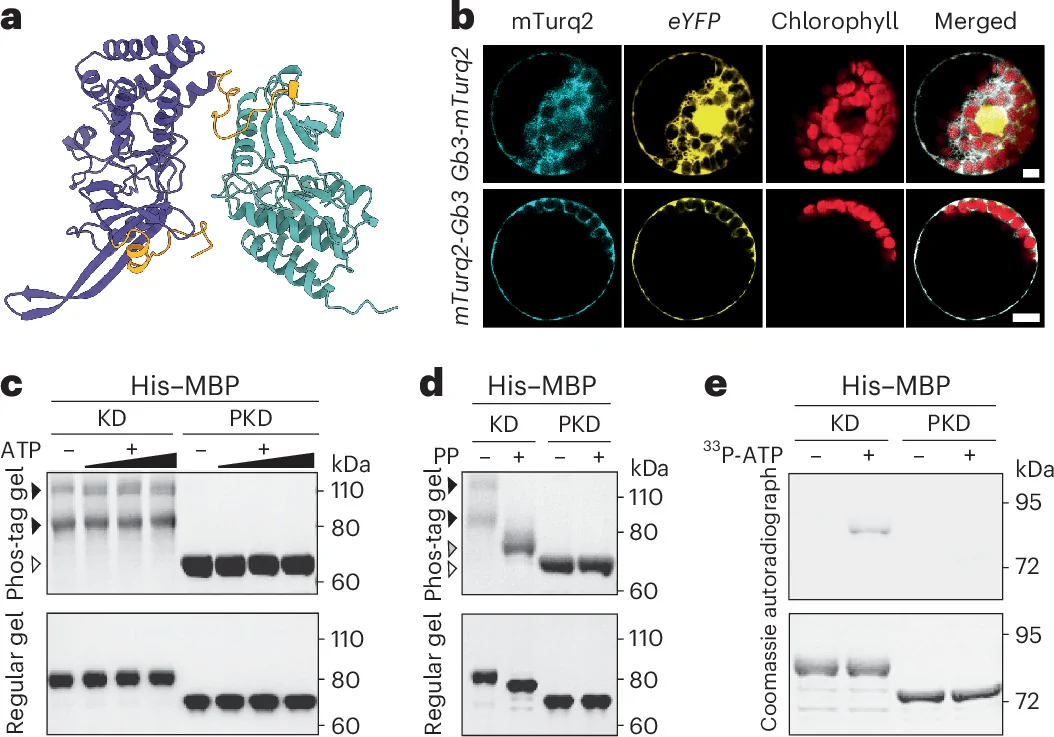
Gb3 protein structure, subcellular localization and kinase activity. **a**, The three-dimensional structure of the protein as modeled by AlphaFold2, where purple and cyan represent two kinase domains, and yellow denotes flexible linkers. **b**, Transient co-expression of a ZmUbi:*Gb3-mTurquoise2* or ZmUbi:*mTurquoise2-Gb3* construct with 35S:*eYFP* (the cytosolic and nuclear marker) in wheat protoplasts. Scale bars, 10 µm. **c**,**d**, Analysis of the autophosphorylation status of the Gb3 kinase domain (His–MBP–KD, 81 kDa) and pseudokinase domain (His–MBP–PKD, 76 kDa) with and without ATP (**c**) and lambda protein phosphatase (PP) (**d**) treatments using Phos-tag and regular SDS–PAGE gels. Recombinant proteins in both gels were detected with an immunoblot using an anti-His antibody. On Phos-tag gels, protein phosphorylation is denoted by a black arrow, dephosphorylation by a gray arrow and lack of phosphorylation by a white arrow. **e**, Assessment of active protein phosphorylation of the Gb3 domains with and without [γ-^33^P]-ATP using SDS–PAGE. Protein loadings were first visualized by Coomassie blue staining, followed by detection of phosphorylated bands via autoradiography. The gel images were cropped to focus on the target proteins. Tick marks on the right of each gel image indicate ladder sizes in kilodaltons (kDa). Source data

The three-dimensional protein structure of Gb3 was predicted using AlphaFold2 (ref. ^41^), revealing a modular configuration consisting of two kinase domains interconnected by a flexible linker (Fig. 3a). An extended β-finger motif was observed within the PK-like domain. To elucidate the functional dynamics of Gb3, we first investigated its subcellular localization within the cellular context. We transiently co-expressed either the N- or C-terminal *mTurquoise2* (mTurq2)-fused Gb3 alongside the cytosolic and nuclear eYFP marker in wheat protoplasts (Fig. 3b). Gb3 is identified as an intracellular protein that operates within the cytosol.

Based on protein sequence analysis, Gb3 exhibits a configuration typical of a kinase–pseudokinase, possessing all nine essential conserved residues within the PK-like domain and eight within the MAP3K-like domain (Extended Data Fig. 3c). To validate this configuration, we expressed and purified recombinant proteins containing either the putative Gb3 kinase domain (His–MBP–KD) or the pseudokinase domain (His–MBP–PKD). Subsequently, an in vitro kinase activity assay using Phos-tag gel was performed to evaluate the phosphorylation status of the Gb3 domains across varying ATP concentrations (Fig. 3c). His–MBP–KD demonstrated autophosphorylation activity, as indicated by an upward shift in phosphoprotein bands, irrespective of the presence or absence of external ATP. By contrast, no phosphorylation was observed for His–MBP–PKD. This finding was further supported by treatment with lambda protein phosphatase, which indicated the dephosphorylation of His–MBP–KD, as reflected by a downward shift in phosphoprotein bands, whereas no shift was noted for His–MBP–PKD (Fig. 3d). Furthermore, an assessment of kinase activity utilizing [γ-^33^P]-ATP confirmed active phosphorylation of His–MBP–KD, while His–MBP–PKD did not display such activity (Fig. 3e). These kinase activity assays support the proposed kinase–pseudokinase architecture of Gb3.

### Histological analysis

**Fig. 4:**
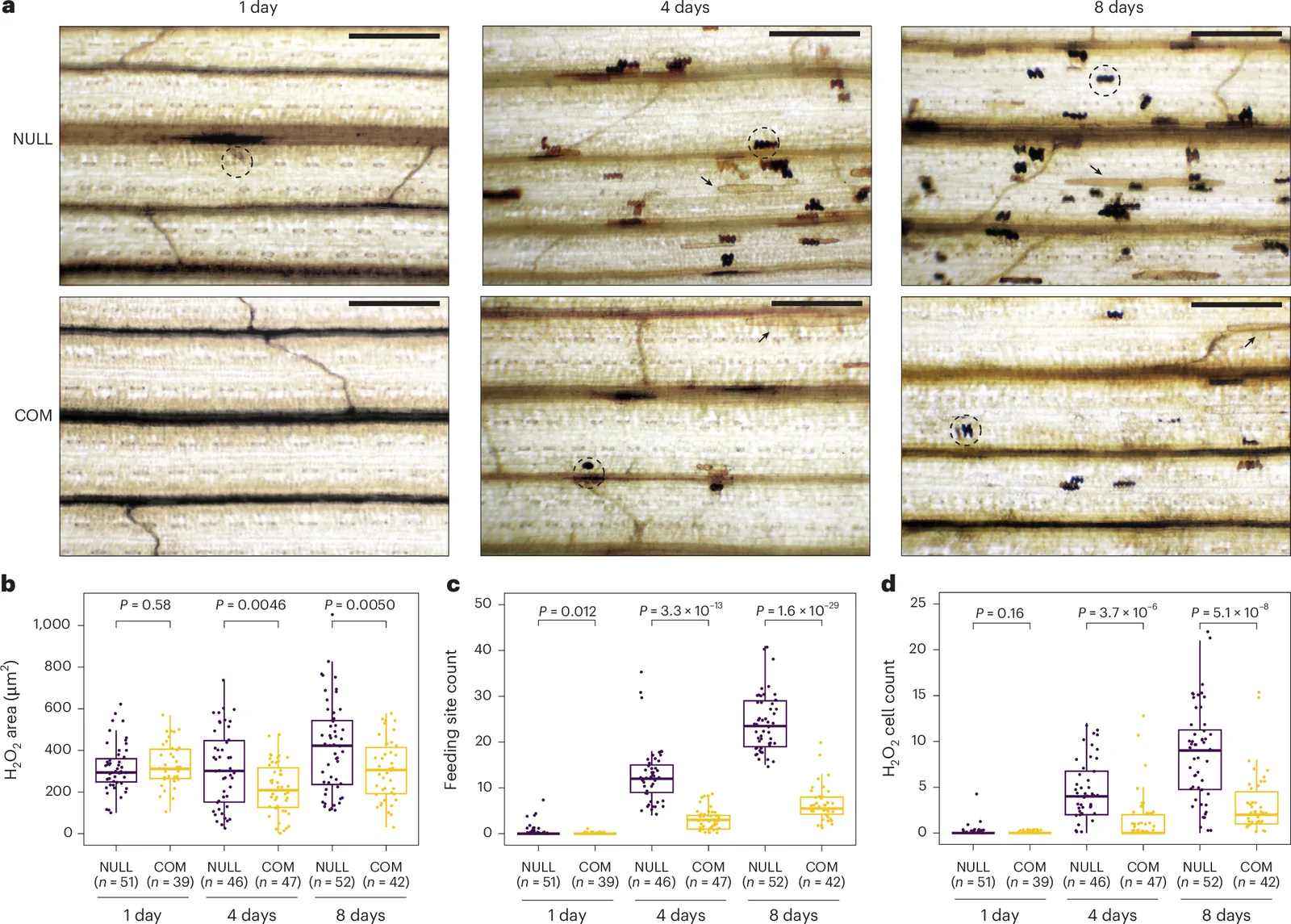
Histological analysis of *Gb3*-mediated cellular response. **a**, Detection of H_2_O_2_ in *Gb3*-complemented and null lines at 1, 4 and 8 days post greenbug infestation with biotype E. Scale bars, 500 µm. **b**, Total area of H_2_O_2_ accumulation on a leaf segment. **c**,**d**, The number of feeding sites (**c**) and adjacent cells (**d**) that accumulated H_2_O_2_. Dashed circles represent feeding sites; arrows highlight adjacent cells exhibiting H_2_O_2_ accumulation in **a**. Box-and-whisker plots show the 25% and 75% percentiles, median, minimum and maximum (**b**–**d**). Statistical significance was assessed using an unpaired, two-tailed *t*-test, with *P* values indicated. COM, complemented. Source data

We investigated the resistance characteristics of *Gb3* in wheat by assessing the host cellular response to greenbug infestation. Reactive oxygen species (ROS) production serves as one of the initial signals that plants activate in response to insect attacks. To evaluate its role in the greenbug response, we used the 3,3′-diaminobenzidine (DAB) staining method to quantify hydrogen peroxide (H_2_O_2_) in both susceptible and resistant lines at 1, 4 and 8 days post infestation with biotype E (Fig. 4). The production of H_2_O_2_ clearly indicates a general response to greenbug attacks in both plant genotypes, accumulating in the leaf vasculature, feeding sites and adjacent cells (Fig. 4a). However, the overall levels of H_2_O_2_ were significantly elevated in susceptible plants, especially at 4 and 8 days post infestation (Fig. 4b). This increase is largely attributed to the significantly higher frequency of feeding sites and surrounding cells that accumulated H_2_O_2_ in susceptible plants (Fig. 4c,d). The reduced number of feeding sites observed in resistant plants strongly highlights the role of *Gb3* in greenbug deterrence.

### Transcriptomic and metabolomic analyses

**Fig. 5:**
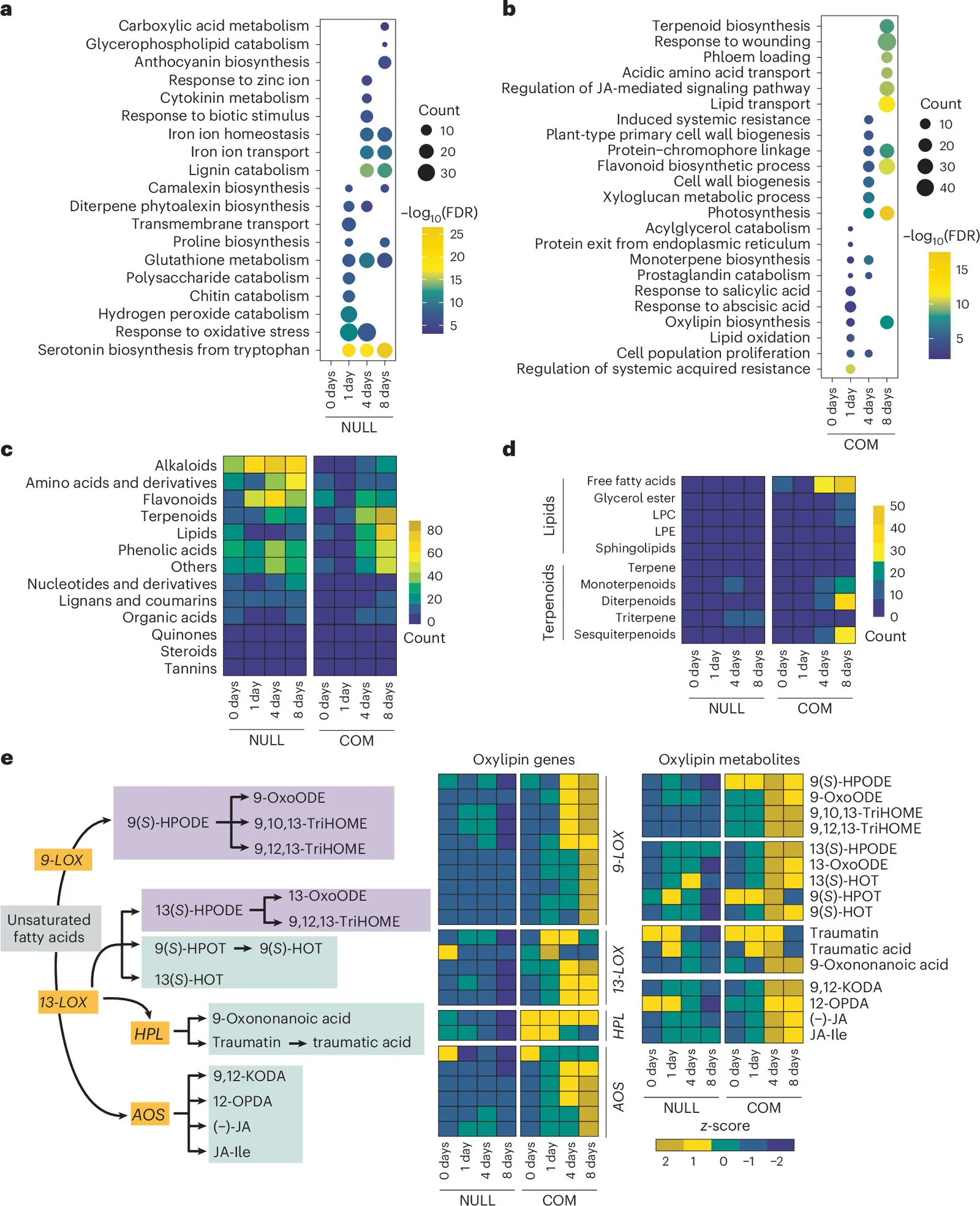
*Gb3*-mediated biological processes and metabolic compositions. **a**,**b**, The top ten biological processes that are enriched in the null lines (**a**) and *Gb3*-complemented lines (**b**) at four time points. **c**,**d**, Changes in the distribution of metabolite classes (**c**) and subclasses (**d**) in null and *Gb3*-complemented lines at four time points. **e**, A schematic representation and heat maps illustrating the biosynthetic genes and metabolites of oxylipins in null and *Gb3*-complemented lines. Gene identifiers are highlighted in yellow. Oxylipins derived from linoleic acid are denoted in purple, while those derived from alpha-linolenic acid are indicated in cyan. The *z*-scores reflect gene expression in fragments per kilobase of transcript per million mapped reads and metabolite ion intensity, scaled within each respective row. LPC, lysophosphatidylcholine; LPE, lysophosphatidylethanolamine; LOX, lipoxygenase; HPL, hydroperoxide lyase; AOS, allene oxidase synthase; 9(*S*)-HPODE, (10*E*,12*Z*)-(9*S*)-9-hydroperoxyoctadeca-10,12-dienoic acid; 9-OxoODE, (10*E*,12*Z*)-9-oxooctadeca-10,12-dienoic acid; 9,10,13-TriHOME, 9,10,13-trihydroxyoctadec-11-enoic acid; 9,12,13-TriHOME, 9(*S*),12(*S*),13(*S*)-trihydroxy-10(*E*)-octadecenoic acid; 13(*S*)-HPODE, (9*Z*,11*E*)-(13*S*)-13-hydroperoxyoctadeca-9,11-dienoic acid; 13-OxoODE, (9*Z*,11*E*)-13-oxooctadeca-9,11-dienoic acid; 9(*S*)-HPOT, (10*E*,12*Z*,15*Z*)-(9*S*)-9-hydroperoxyoctadeca-10,12,15-trienoic acid; 9(*S*)-HOT, (9*S*)-(10*E*,12*Z*,15*Z*)-9-hydroxyoctadecatri-10,12,15-enoic acid; 13(*S*)-HOT, (9*Z*,11*E*,15*Z*)-(13*S*)-13-hydroxyoctadeca-9,11,15-trienoic acid; 9,12-KODA, 9-hydroxy-12-oxo-10(*E*),15(*Z*)-octadecadienoic acid; 12-OPDA, (15*Z*)-12-oxophyto-10,15-dienoic acid; (–)-JA, (-)-jasmonic acid; JA-Ile, (–)-jasmonoyl-l-isoleucine.

**Extended Data Fig. 7:**
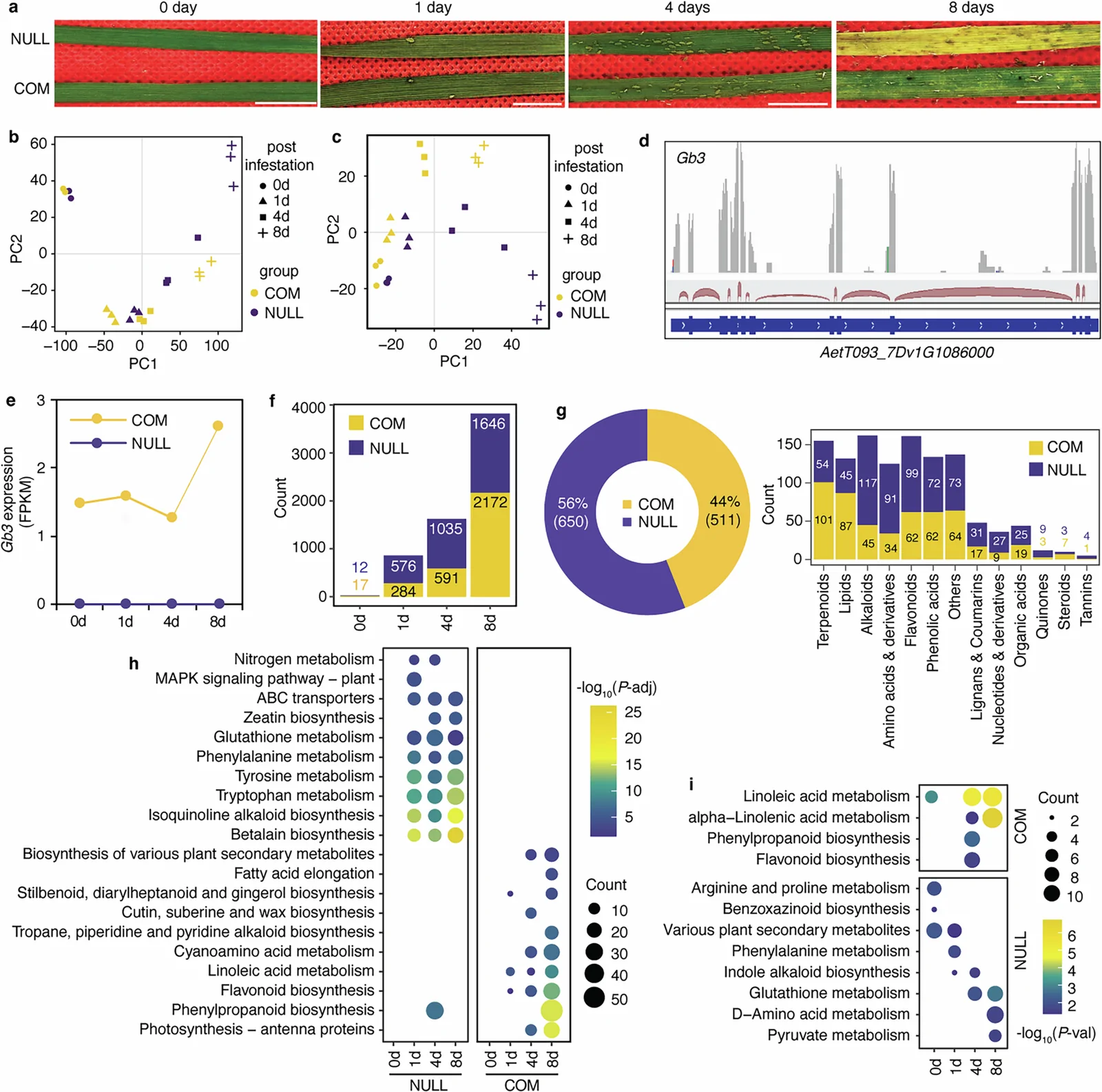
Transcriptomic and metabolomic analyses of *Gb3*-complemented and null lines at four timepoints. **a**, Leaf phenotype. Scale bar = 1 cm. **b, c**, Principal component analyses on the gene expression FPKM values (**b**), and the ion intensity of metabolites (**c**) of all three biological replicates per line at each timepoint. **d**, RNA-seq reads mapped to the *Gb3* locus (*AetT093_7Dv1G1086000*), with splice junctions shown in a Sashimi plot. **e**, *Gb3* expression in transgenics. **f**, Number of differentially upregulated genes. **g**, Total number of differentially upregulated metabolites. **h** and **i**, KEGG enrichment analyses for the up-regulated genes (**h**) and metabolites (**i**). A hypergeometric test was applied to identify significantly enriched pathways, with the Benjamini–Hochberg (BH) method used for multiple testing correction. Pathways with an adjusted *P*-value < 0.05 were considered statistically significant (**h** and **i**).

To understand how greenbugs affect the host wheat plant and the role *Gb3* has in promoting resistance, we conducted transcriptomic and metabolomic analyses at 0, 1, 4 and 8 days post infestation (Fig. 5 and Extended Data Fig. 7). Our transcriptomic analysis indicates that susceptible plants activate genes associated with oxidative stress response, H_2_O_2_ catabolism, glutathione metabolism, serotonin biosynthesis and iron homeostasis upon greenbug infestation (Fig. 5a). By contrast, *Gb3* in resistant plants initiates the activation of transcripts linked to systemic acquired resistance regulation, salicylic acid (SA) and abscisic acid (ABA) responses, and oxylipin and monoterpene biosynthesis one day after greenbug infestation (Fig. 5b). As the infestation progresses, *Gb3* promotes the expression of genes involved in photosynthesis; oxylipin, flavonoid and terpenoid biosynthesis; wounding response; jasmonic acid (JA) signaling; and lipid and amino acid transport (Fig. 5b). Metabolic profiling reveals that susceptible plants significantly accumulate alkaloids, flavonoids and amino acids following greenbug attack (Fig. 5c). By contrast, resistant plants with *Gb3* exhibit a significant increase in terpenoids, lipids and phenolic acids, particularly at 8 days post infestation (Fig. 5c). Among the terpenoids, diterpenoids and sesquiterpenoids are key components (Fig. 5d). Free fatty acids emerge as the dominant lipid component, leading to an array of oxylipins, as supported by the expression of the related biosynthetic genes (Fig. 5d,e). These oxylipins, terpenoids and phenolic acids are well-recognized for their repellent and insecticidal properties^42–47^.

### *Gb3* evolution in Triticeae tribe

**Fig. 6:**
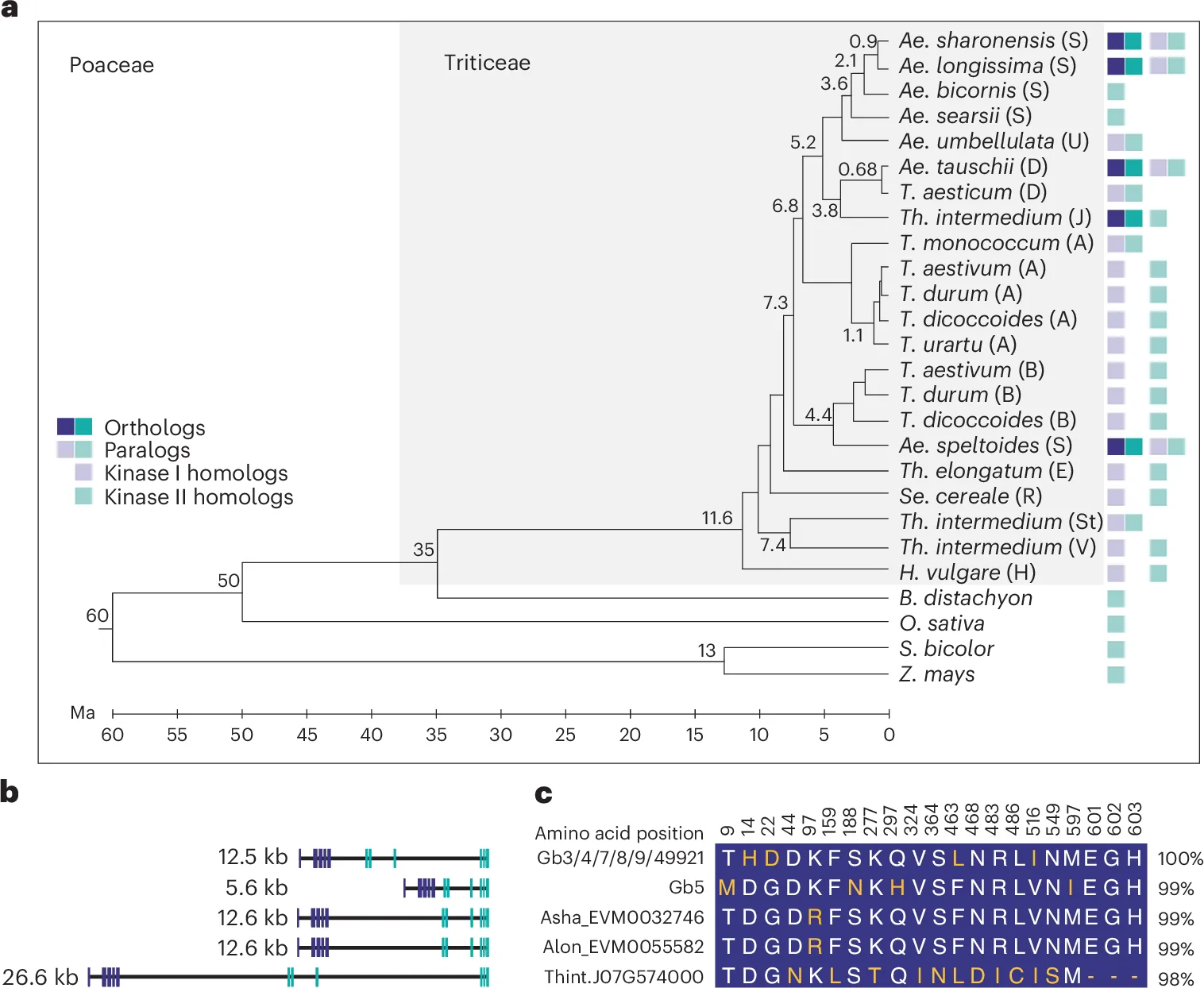
Evolution and distribution of Gb3 orthologs. **a**, Phylogeny showing the Gb3 orthologs, paralogs and domain homologs in the Poaceae family. The Triticeae clade is highlighted in gray. The subgenome of each species is indicated in parentheses. **b**, Comparison of the gene structures of *Gb3* and its orthologous genes. **c**, Gb3 allelic variants based on orthologous protein sequences. Percent identity to Gb3 is presented on the right. Gb3/4/7/8/9/49921 (*Ae. tauschii*), Gb5 (*Ae. speltoides*), Asha_EVM0032746 (*Ae. sharonensis*), Alon_EVM0055582 (*Ae. longissima*) and Thin.J07G574000 (*Th. intermedium*). *Ae.*, *Aegilops*; *B.*, *Brachypodium*; *H.*, *Hordeum*; *O.*, *Oryza*; *Se.*, *Secale*; *S.*, *Sorghum*; *T.*, *Triticum*; *Th.*, *Thinopyrum*; *Z.*, *Zea*.

**Extended Data Fig. 8:**
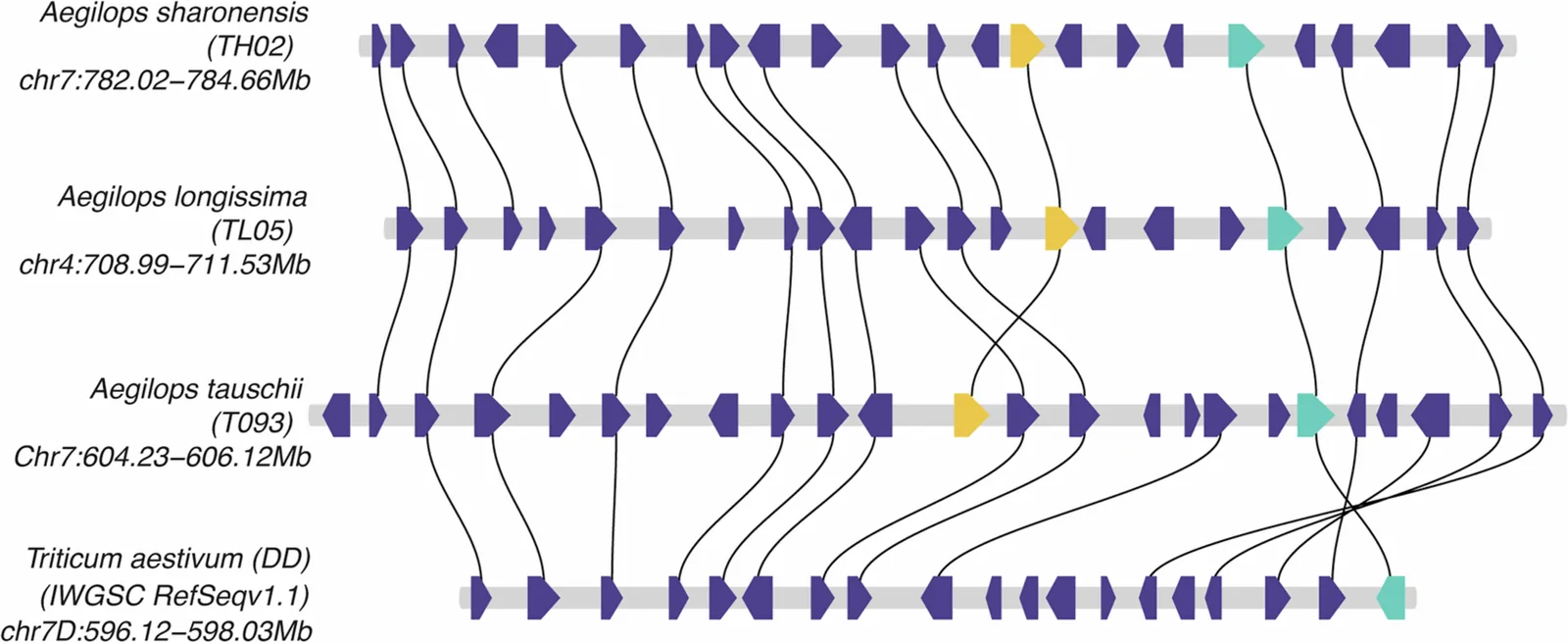
Gb3 orthologs and paralogs in *Aegilops* species. Collinearity of the *Gb3* locus in *Aegilops* species compared to bread wheat. Gb3 orthologs are presented in yellow and paralogs in cyan. Whole genome of *Ae. speltoides* containing *Gb5* has not been sequenced, thus excluded from comparison.

To explore the evolutionary history of *Gb3*, we analyzed 270 publicly available plant genomes representing 52 distinct monocot species (Supplementary Table 6). Gb3 orthologs were identified exclusively in the Triticeae tribe, specifically in these five species: *Thinopyrum intermedium*, *Ae. tauschii*, *Aegilops sharonensis*, *Aegilops longissima* and *Aegilops speltoides* (Fig. 6a). This indicates that Gb3 and its orthologs probably emerged between 5.2 million years ago (Ma) and 7.3 Ma. In *Aegilops* species harboring Gb3 orthologs, we identified paralogs exhibiting approximately 80% identity within a 1-Mbp distance (Extended Data Fig. 8 and Supplementary Table 7). This suggests a prior gene duplication event followed by the subsequent deletion of Gb3 orthologs in numerous accessions of *Aegilops* species. A homolog search further revealed that kinase II is present across all Poaceae species, whereas kinase I was initially detected in *Hordeum vulgare* (Fig. 6a and Supplementary Table 7). These two kinase domains were highly distinct (Extended Data Fig. 3b,c), implying that Gb3 probably originated from the fusion of two protein domains. This fusion event probably occurred between 7.3 Ma and 11.6 Ma in the Triticeae, where the Gb3 paralog was initially detected in the St subgenome of *Th. intermedium* (Fig. 6a).

### *Gb3* marker development and allelic assessment

**Extended Data Fig. 9:**
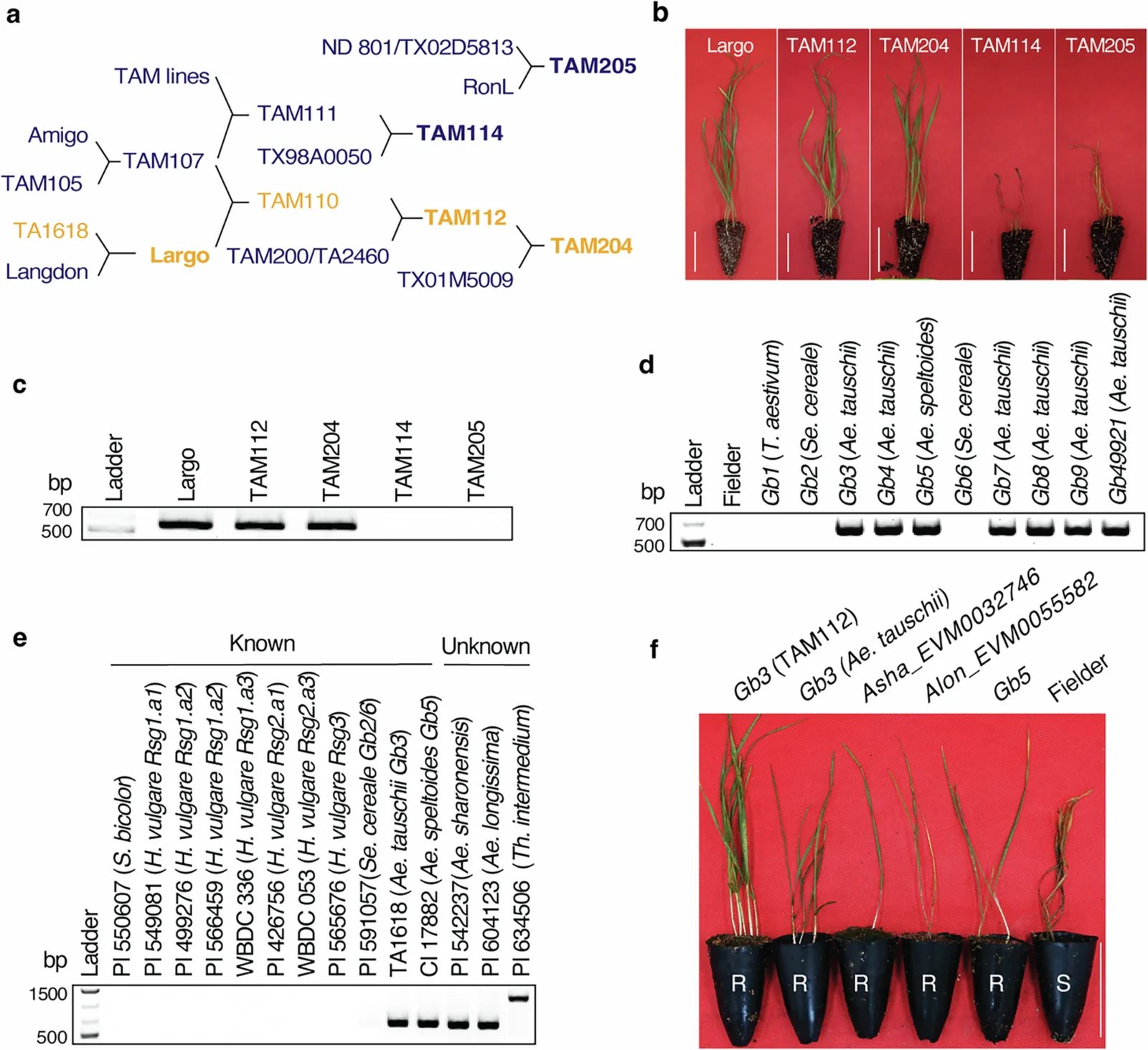
Development and assessment of the *Gb*3 diagnostic marker in different species. **a**, Origins of greenbug resistance (yellow) and susceptibility (purple) in TAM cultivars. **b**, Phenotypic screening of TAM cultivars with and without *Gb3* against greenbug biotype E. Scale bar = 5 cm. **c**, Development of a PCR-based diagnostic marker. **d** and **e**, Detection of the *Gb3* marker in accessions carrying *Gb-*designated genes (**d**) and in different species known and unknown to confer greenbug resistance (**e**). Resistance donor species are indicated in brackets. The gel images were cropped to focus on the expected size of the *Gb3* marker; full-length gels can be found in the Source Data file. **f**, Phenotypic screening of *Aegilops* species carrying Gb3 allelic variants against greenbug biotype E. Species include *Ae. tauschii* (*Gb3*), *Ae. sharonensis* (*Asha_EVM0032746*), *Ae. longissima* (*Alon_EVM0055582*), and *Ae. speltoides* (*Gb5*). TAM112 and Fielder were used as controls. Scale bar = 5 cm. Source data

To support breeding efforts, we developed a diagnostic PCR marker that distinguishes resistant TAM cultivars from susceptible ones by detecting the presence or absence of *Gb3* (Extended Data Fig. 9a–c). Using this marker, a comprehensive evaluation of *Gb3* was enabled in accessions and lines from various Poaceae species, including some with known greenbug resistance and others without (Extended Data Fig. 9d,e). The *Gb3* marker was detected only in the five Triticeae species mentioned above, indicating that resistance in other species is mediated by alternative *Gb* genes. The synthetic hexaploid wheat lines carrying six *Gb* genes from *Ae. tauschii* and one from *Ae. speltoides* all contained the *Gb3* marker (Extended Data Fig. 9d).

A comparative analysis of full-length genomic sequences of *Gb3* orthologous genes revealed the conservation of all exons; however, variable intron sizes at positions 5 and 8 across different species suggest the occurrence of an indel event during evolution (Fig. 6b). Moreover, the gene structures and protein sequences among the six *Gb* genes from *Ae. tauschii* were identical (Fig. 6b,c), strongly suggesting that they represent the same gene (*Gb3*) that has been independently identified in various genetic backgrounds and designated with different names. The differences in their resistance profiles are, therefore, due to distinct genetic backgrounds. In addition, the Gb3 orthologs in *Ae. sharonensis* (Asha_EVM0032746) and *Ae. longissima* (Alon_EVM0055582) shared the same allele with identical gene structures and protein sequences (Fig. 6b,c). The *Aegilops* accessions carrying these alleles show resistance to greenbug biotype E (Extended Data Fig. 9f), which can be attributed to either *Gb3* allelic variants or other novel R genes present in those species. Furthermore, different *Aegilops* species may display varying resistance profiles, as indicated by the distinct responses of *Gb3* in *Ae. tauschii* and *Gb5* in *Ae. speltoides* (Supplementary Table 1). These differences can result from diverse genetic compositions that encompass additional genes for biotype-specific resistance.

## Discussion

R gene cloning for insects has fallen far behind that for pathogens, with ten genes identified in plants. These genes include *Mi-1* in wild tomato and *Vat* in melon for aphid resistance^22,48^, *Sm1* in wheat for midge resistance^20^, and *BPH3*, *BPH6*, *BPH9*, *BPH14*, *BPH29*, *BPH30* and *BPH32* in rice for resistance against the brown planthopper^23–29,49,50^. Among these, *Mi-1*, *Vat*, *BPH9*, *BPH14* and *Sm1* encode NLR proteins, with *BPH9* featuring two NBS domains and *Sm1* additionally containing kinase and MSP domains^20,22–24,48^. *BPH3* includes a cluster of three plasma membrane (PM)-localized lectin receptor kinases^29^, while the remaining *BPH* genes do not encode typical R proteins. These include *BPH6*, which encodes an uncharacterized exocyst-localized protein^28^, *BPH30*, an endomembrane protein with two leucine-rich domains^27^, *BPH32*, a PM-localized protein with a short consensus repeat domain^25^, and *BPH29*, a nuclear-localized protein containing a B3 domain^26^.

In this study, we cloned *Gb3*, a greenbug R gene identified in *Ae. tauschii*, which confers resistance to numerous greenbug biotypes in wheat. Notably, *Gb3* encodes a KFP with a PK-like and a distinct MAP3K-like domain to confer insect resistance. Although 11 TKs have been cloned, the classification of their domains as kinases or pseudokinases was confirmed only for Pm36^12^ and RWT4^19^, while the remaining were inferred from conserved residues. We conducted two kinase activity assays and validated the *Gb3* kinase–pseudokinase architecture. Similar to most TKs, Gb3 also evolved to function as an intracellular protein specifically within the Triticeae tribe^4,51^. Recent studies revealed that TKs, including Sr62^TK^, RWT4/WTK3 and Lr41, operate as intracellular sensors that recognize effector proteins via their kinase or pseudokinase domains harboring extended β-finger motifs^4,16–18^. This recognition initiates TK interaction with a downstream NLR to activate plant immunity. These findings suggest that the Gb3 kinase domain, which carries the extended β-finger motif, may play a role in effector recognition. The Gb3 pseudokinase domain may function as a decoy to facilitate effector binding and subsequently activate a downstream target related to greenbug resistance.

Plant resistance to fungal pathogens involves oxidative bursts and cell death, aimed at restraining pathogen spread^3,4^. By contrast, greenbugs, which are phloem-feeding aphids, can migrate to establish new feeding sites. Host resistance to aphids can be categorized into three mechanisms: antixenosis (deterrent effect), antibiosis (toxic effect) and tolerance (ability to withstand damage)^52^. These mechanisms are underpinned by recognition processes, phytohormonal signaling and secondary metabolites^53^. Aphid R genes such as *Mi-1* and *Vat*, as well as brown planthopper R genes such as *BPH6* and *BPH9*, confer both antixenosis and antibiosis^22,24,28,54,55^, while *Sm1*, *BPH14* and *BPH32* operate via antibiosis^23,25,47^. Importantly, *Mi-1* and *BPH14* have been shown to trigger oxidative bursts and SA signaling without inducing cell death, a response typically associated with pathogen resistance^50,55^. *Gb3*, akin to other aphid R genes, provides both antixenosis and antibiosis resistance^56^. Greenbug biotype E exhibits a preference for feeding on the stems of susceptible plants but favors leaves over stems in resistant varieties^56^. This preference probably results from the induction of *Gb3* expression in the stem during infestation, while expression is reduced in the leaves (Supplementary Fig. 1). Histological analysis suggests that *Gb3* can still deter greenbug feeding on leaves, probably through systemic signaling.

Omics analyses offer insights into greenbug susceptibility and *Gb3*-mediated resistance mechanisms in host plants. Plants produce secondary metabolites—such as terpenoids, phenolic compounds, alkaloids and flavonoids—as integral components of their defenses against insect threats, acting as deterrents, antifeedants or toxins^42,47,52,53,57–61^. Susceptible plants may initially deter greenbugs using alkaloids and flavonoids. However, greenbugs, similar to other aphids, may have evolved mechanisms to detoxify, sequester and/or excrete such defense metabolites^59,61–63^, allowing them to feed on susceptible plants. Their feeding triggers the secretion of effector proteins that can suppress plant immunity^64,65^. In response, susceptible plants generate an oxidative burst to activate a basal defense. To manage oxidative stress, they also express genes involved in H_2_O_2_ degradation, detoxification of iron-dependent ROS via glutathione metabolism and iron homeostasis, and synthesis of defensive and antioxidant compounds such as alkaloids (serotonin and camalexin) and anthocyanins^53,61,66–69^. Conversely, greenbugs appear to possess ROS-detoxifying mechanisms, which they may exploit to induce early leaf senescence (Extended Data Fig. 7a) to remobilize nutrients for their benefit^53,61,70^. Consequently, susceptible plants become nutrient-depleted and ill-equipped to withstand oxidative stress and greenbug damage, accelerating their decline.

By contrast, resistant plants expressing *Gb3* utilize limited stores of flavonoids, oxylipins and monoterpenes for baseline defenses, which are probably released upon greenbug contact^71,72^. In addition, the emission of volatile organic compounds such as monoterpenes can trigger systemic signals that prime plants for defense^73^. When greenbugs feed, they may release an effector recognized by *Gb3*, undermining the insect’s ability to bypass the plant’s immune response. This recognition potentially stimulates a downstream target to initiate transcriptional reprogramming that repairs cell walls and produces various defensive compounds, including oxylipins, terpenoids and phenolic acids. Remarkably, *Gb3* induces terpenoid production at a rate twice that of susceptible plants (Extended Data Fig. 7g). Volatile organic compounds such as monoterpenes and sesquiterpenes function as repellents and toxins, while diterpenes can disrupt insect cell membranes^42–44,57,60,61^. In addition, the oxylipin biosynthesis pathway remains continuously activated by *Gb3* during greenbug infestation, highlighting its critical role in plant resistance. Oxylipins such as JA, OPDA and KODA enhance resistance by impacting insect growth^46,53,74,75^, with JA application effectively deterring greenbug infestations in sorghum^45^. *Gb3*, as an unconventional R gene, distinguishes itself from typical NLRs by using JA rather than SA for hormonal signaling related to aphid resistance^24,50,53,55^.

The resistance response attributed to *Gb3* is conserved across various *Aegilops* species. We have identified functional allelic variants of Gb3 in *Ae. speltoides* (Gb5), *Ae. sharonensis* (Asha_EVM0032746) and *Ae. longissima* (Alon_EVM0055582). In addition, we consolidated six *Gb* genes located on the 7DL chromosome of *Ae. tauschii* into a single genetic locus (*Gb3/4/7/8/9/49921*). Similar trends are observed among other TK genes; for instance, *Yr15*, *YrG303* and *YrH52* from wild emmer wheat all encode the same WTK1 protein^76^, while *Lr9* and *Lr58* encode the same *WTK6-vWA* from *Ae. umbellulata*^13^. *Pm24 (WTK3)* from hexaploid wheat^9^ and *RWT4* from *Ae. tauschii*^17^ are recognized as allelic variants. These genes were previously regarded as distinct owing to differing resistance profiles arising from variations in alleles or genetic backgrounds. For instance, while *Gb3* alone does not confer resistance to biotype TX1 in wheat, association mapping has identified it as the major peak associated with TX1. However, a minor peak on chromosome 3DL was specifically linked to TX1 but not to other biotypes (Extended Data Fig. 1e). The novel gene at that locus seems to represent a rare allele segregating in the *Ae. tauschii* panel for TX1-specific resistance (Supplementary Table 4), implying that the resistance allele is probably absent or nonfunctional in wheat backgrounds. This 3DL-locus gene may operate downstream of *Gb3* to provide TX1-specific resistance or function independently by recognizing TX1-specific effectors. Such biotype-specific resistance could point to additional *Gb* genes.

**Extended Data Fig. 10:**
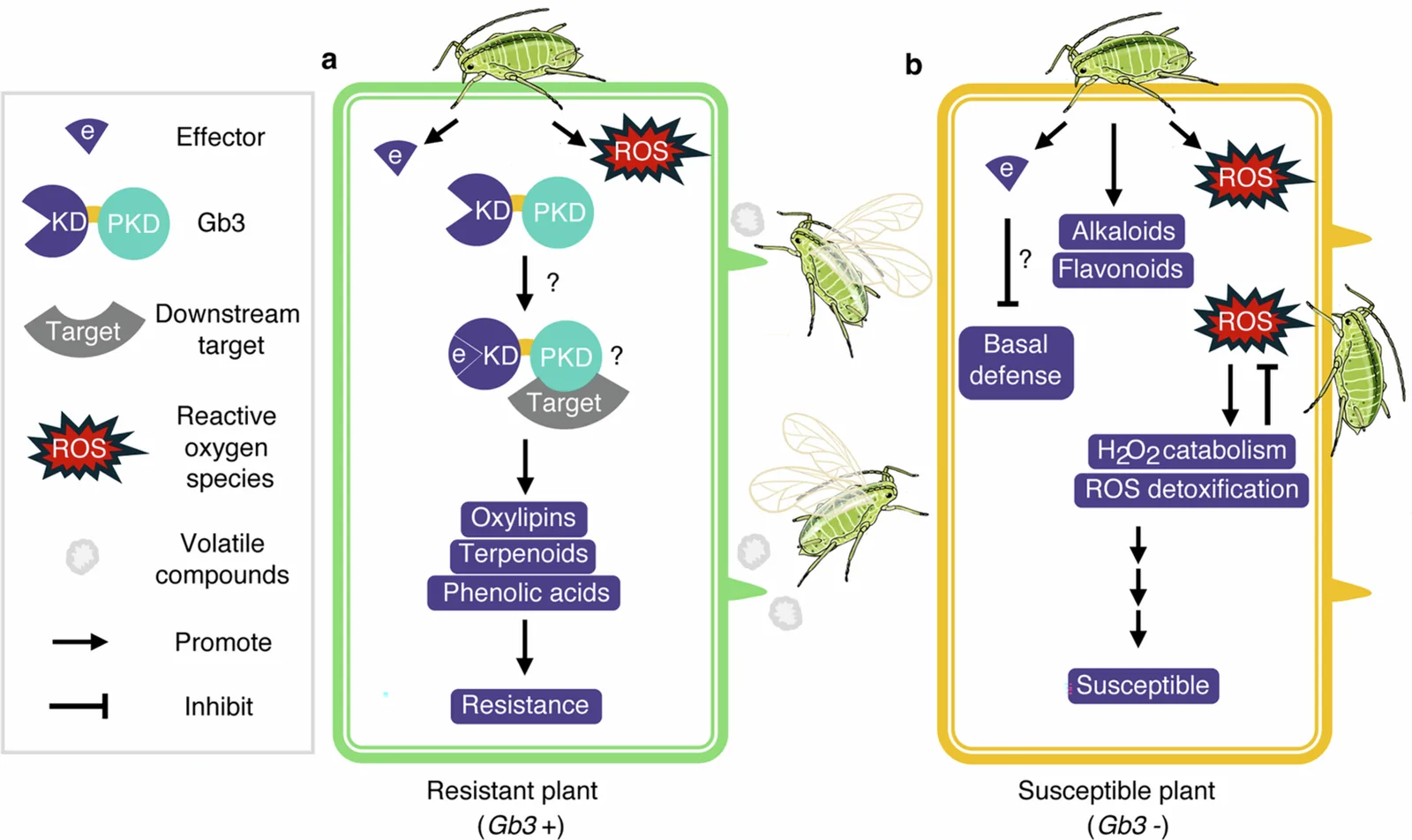
Proposed models of plant resistance (*Gb3*+) and susceptibility (*Gb3*-) to greenbug infestation. **a**, When greenbugs contact a plant surface, they trigger the release of volatile compounds, which can generate systemic signals that prime resistant plants for defense. Upon feeding, Gb3, an intracellular receptor in resistant plants, recognizes and binds to an effector released by greenbugs. This activates a downstream target that triggers the accumulation of defense metabolites such as oxylipins, terpenoids, and phenolic acids, ultimately promoting greenbug resistance. **b**, When effector recognition fails, greenbugs may suppress basal defense, neutralize defense compounds and exploit oxidative stress to release nutrients, resulting in increased susceptibility.

In summary, we propose that the *Gb3*-encoding kinase–pseudokinase protein serves as an intracellular receptor, recognizing specific greenbug effectors (Extended Data Fig. 10). This interaction may activate downstream pathways related to transcriptional and metabolic reprogramming, leading to the production of defense compounds that confer greenbug resistance. If the recognition of these effectors fails, greenbugs may bypass basal plant immunity and exploit oxidative stress to redirect nutrients for their advantage. Further research is essential to fully understand the sensor functions of *Gb3* and to identify its interacting effectors and downstream targets, which are critical for initiating resistance signaling. Moreover, identifying specific effectors across various greenbug biotypes and elucidating how these effectors induce susceptibility will be invaluable. This knowledge can inform the development of innovative strategies to counteract greenbug invasions and assist in breeding cultivars with broad-spectrum resistance.

## Methods

### Genome-wide association

The phenotypic responses of the *Ae. tauschii* panel consisting of 260 accessions to greenbug biotypes C, E, I and TX1 were evaluated in our earlier study^38^. The responses were transformed into numerical values for association mapping, with scores ranging from −2 (susceptible) to 2 (resistant) (Supplementary Table 2). The panel was previously genotyped^10^, and single-nucleotide polymorphisms (SNPs) in VCF format were retrieved from Zenodo at https://doi.org/10.5281/zenodo.4317950, and filtered using the same parameters and code for the 260 accessions (https://github.com/wheatgenetics/owwc). This process retained about 15 million SNPs across seven chromosomes of the *Ae. tauschii* reference genome AL8/78 (ref. ^39^). Genome-wide association between greenbug responses and SNP markers was performed using the *vcf2gwas* pipeline (https://github.com/frankvogt/vcf2gwas)^77^ and the Manhattan plot was generated using the R package CMplot (https://github.com/YinLiLin/CMplot).

### Candidate gene identification

Micro-collinearity among pangenome sequences of greenbug-resistant and susceptible *Ae. tauschii* accessions on the chromosome 7DL segment was assessed using LASTZ alignment^78^ in Geneious Prime 2024.0.7. Pangenome sequences were retrieved from a previous study^14^. High-confidence gene annotations were derived from the *Ae. tauschii* T093 reference genome^79^.

### Haplotype, domain and protein structure analyses

The de novo-assembled (MEGAHIT) sequences for the *Ae. tauschii* panel were retrieved^10^ and anchored to the T093 reference genome to assign genomic coordinates using the codes provided in the kGWAS pipeline (https://github.com/wheatgenetics/owwc). The candidate gene sequences for the panel were extracted via nucleotide blast against the anchored de novo assemblies. Domain searches for *Gb3*, its orthologs, and homologs were performed using InterPro version 101.0^80^ and National Center for Biotechnology Information’s (NCBI) Conserved Domain databases (CD-Search)^81^. The three-dimensional protein model of *Gb3* was predicted using AlphaFold2 (ref. ^41^).

### Engineering and genotyping *Gb3*-complemented transgenic lines

The predicted full-length *Gb3* gene, spanning 16,108 base pairs (bp) was cloned, including 2,564-bp upstream and 1,053-bp downstream regulatory regions. In brief, three overlapping fragments of *Gb3* were PCR-amplified from TA1618 (*Gb3* donor) genomic DNA using PrimeSTAR GXL DNA Polymerase (TaKaRa, R050A). The PCR products were purified using the QIAquick PCR Purification Kit (QIAGEN, 28106), primarily subcloned and assembled into the pJET1.2 cloning vector (Thermo Fisher scientific, K1232) using restriction enzymes. The assembled sequence was validated by nanopore sequencing. The verified full-length *Gb3* gene was then re-amplified and subsequently cloned into the linearized binary vector JD561 (ref. ^82^), which was digested using HindIII and *Spe*I. The finalized binary construct was sequence-validated through full plasmid sequencing, and the transfer DNA (T-DNA) was introduced into the susceptible wheat cultivar (cv.) Fielder using Growth-Regulating Factor 4 (GRF)–GRF Interacting Factor 1 (GRF–GIF)-assisted *Agrobacterium*-mediated (AGL1 strain) transformation at the Plant Transformation Facility of University of California (Davis, CA, USA) (https://ptf.ucdavis.edu/). *Gb3*-specific and hygromycin-specific primer sets were used to genotype T_0_-, T_1_- and T_2_-complemented and null plants. The T-DNA (*hygromycin phosphotransferase* II, *hpt*II) copy number in T_0_ and T_1_ plants was quantified by digital droplet PCR using wheat *PINb* (*PUROINDOLINE-b*) as the internal reference gene control^83^. All primers used to generate binary constructs and for genotyping transgenics are listed in Supplementary Table 8.

### Engineering and genotyping *Gb3* knockout transgenic lines

Three guide RNAs (gRNAs) targeting the *Gb3* gene were designed using the CRISPR MultiTargeter platform^84^. The gRNA scaffold and transfer RNA sequences were amplified from the pGTR construct^85^ and assembled using NEBuilder HiFi DNA Assembly (NEB, E5520S). The gRNA–transfer RNA amplicons were digested with BsaI and ligated into an AarI-linearized binary vector, JD633 (ref. ^82^). The integrity of the construct was confirmed through full plasmid sequencing, followed by transformation into the *Agrobacterium* strain AGL1. The resulting T-DNA construct was introduced into *Gb3*-containing wheat cv. TAM112 to generate transgenic knockout lines by the Plant Transformation Facility of University of California (Davis, CA, USA). The T_0_ transformants and their T_1_ progenies were genotyped using a cleaved amplified polymorphic sequences assay targeting the SmaI recognition site at gRNA3, followed by amplicon Sanger sequencing of edited lines. Different alleles of the edited lines were decoded using DECODR version 3.0^86^. Primers used to generate the editing construct for *Gb3* and for genotyping putative edits are listed in Supplementary Table 8.

### Evaluation of transgenic lines and *Aegilops* species for greenbug resistance

Phenotypic screenings were carried out in a greenhouse under controlled conditions at the US Department of Agriculture-Agriculture Research Service (USDA-ARS), Peanut and Small Grains Research Unit in Stillwater, OK, USA. Eight greenbug biotypes (C, E, I, K, OK3, WY5, WY9 and WY86) were increased on barley cv. Eight-Twelve (PI 537437). About 50–60 seeds were planted in each of the 15-cm pots, which were enclosed in a cylindrical plastic cage fit with muslin cloth ventilation at the top and sides. Seedlings at the two-leaf stage were infested by putting aphids on barley leaves. Barley plants covered with abundant aphids after 3 weeks were used to evaluate responses of transgenics and *Aegilops* species to each biotype.

For *Gb3*-complemented lines, 50–100 segregating T_1_ progenies from four independent transgenic lines were initially screened for resistance to biotype E in a greenhouse maintained at 22 ± 2 °C with a photoperiod of 16 h of light and 8 h of dark. A randomized complete block design with two replicates was used. In each replicate, 25–50 seeds from each line were planted in 5–10 consecutive cells of a 73-cell growing tray filled with BM2 Berger Plug Soil (Berger), with 5 seeds in each cell. Plantlets were infested by placing culture plants covered with greenbugs between the rows 2 days after emergence. TAM112 or TAM204 (*Gb3*^+^) and Fielder (*Gb3*^−^) were planted in each tray as controls. Phenotypes were evaluated approximately 2 weeks after infestation, when the susceptible control, Fielder, exhibited complete susceptibility. Plants that grew normally without damage symptoms were rated as resistant, whereas those showing yellowness or withering or were dead were rated as susceptible.

For the T_2_ generation, 20 plantlets per line were screened against eight greenbug biotypes (C, E, I, K, OK3, WY5, WY9 and WY86), using two independent *Gb3*-complemented and null lines, each with three biological replicates. Nontransgenic TAM112 (*Gb3*^+^) and Fielder (*Gb3*^−^) were used as resistant and susceptible controls, respectively. For CRISPR-edited lines, 20 segregating T_1_ progenies from three to four independent *Gb3*-knockout transgenics, along with nontransgenic TAM112 (*Gb3*^+^) and TAM114 (*Gb3*^−^) controls, were screened against five biotypes (C, E, I, K and OK3). In addition, ten seedlings from T_2_ knockout lines with six different indel events were rescreened against biotypes C, E and K, with the assay repeated twice. For different *Aegilops* species, about five seedlings were screened twice against biotypes E.

### Agronomic and yield evaluation of transgenic lines

The experimental design followed a randomized complete block design, comprising a total of 12 flow trays (68 cm × 38.5 cm × 13 cm; Stuewe and Sons). The experiment included two independent *Gb3*-complemented lines and their corresponding null lines, with three biological replicates and two technical replicates per biological replicate (that is, two trays per replicate). Trays were filled with professional growing mix (Sun Gro Horticulture) uniformly premixed with 80 g of slow-release fertilizer (15–9–12 N–P–K). To ensure uniform germination, seeds were pretreated at 4 °C in sterilized water for 1 week. In each tray, nine seeds from a *Gb3*-complemented line and nine seeds from its corresponding null line were sown adjacently in two rows, with 20-cm spacing between rows and 8-cm spacing between seeds within each row. Plants were grown in a greenhouse at 21 °C under a 16-h photoperiod for 3.5 months, with uniform watering applied as needed. The entire experiment was conducted twice. Seven agronomic and yield traits were assessed, and the combined data from the two experiments are presented. Plant height and the number of spikes were recorded for each plant, with the average calculated per tray. For spike length, spikelet number per spike and seeds per spike, measurements were taken from ten representative spikes selected from each line within a tray, and averages were calculated per tray. For thousand kernel weight, seeds from each line in a tray were pooled, counted and weighed, and kernel weight per line in a tray was quantified accordingly.

### Subcellular localization

The coding sequence (CDS) of *Gb3* was fused to mTurq2 (ref. ^87^) at either the N- or C-terminus, and expressed under the maize ubiquitin-1 promoter (ZmUbi) and NOS terminator. The expression vector backbone was PCR-amplified (Supplementary Table 8) from ZmUbi*:mTurquoise2* and assembled with the *Gb3* CDS using NEBuilder HiFi DNA Assembly (NEB, E5520S). The eYFP construct was derived from the organelle marker excluding the transit peptide^88^, which was expressed under a 35S promoter with dual enhancer elements and NOS terminator. Protoplasts were isolated from 7–8-day-old Fielder seedlings grown under an 8-h light/16-h dark photoperiod. ZmUbi:*Gb3-mTurquoise2* or ZmUbi:*mTurquoise2-Gb3* was co-transfected with the cytosolic and nuclear marker 35S:*eYFP* into protoplasts according to ref. ^89^. Fluorescence was captured 16–20 h post transfection using a Leica TCS SP8 confocal microscope with a 63× oil-immersion objective. The fluorescent signals were detected at the following wavelengths: mTurq2 (excitation (Ex) 458 nm, emission (Em) 460–490 nm), eYFP (Ex 514 nm, Em 514–550 nm) and chlorophyll autofluorescence (Ex 561 nm, Em 660–690 nm).

### Protein expression and purification from *E. coli*

The CDS of two kinase domains of *Gb3* were codon-optimized, synthesized and cloned into *E. coli* expression vector pET28a-His-MBP (ref. ^19^) commercially (GenScript). In brief, the recombinant proteins, His–MBP–KD (GenBank: PZ366436) and His–MBP–PKD (GenBank: PZ366435), were expressed in the *E. coli* strain BL21 Star (DE3) by growing the bacterial culture at 37 °C to an optical density at 600 nm of 1.2. The expression of recombinant proteins was induced by adding 0.5 mM isopropyl β-d-thiogalactopyranoside (IPTG) at 15 °C for 16 h. The recombinant proteins were purified using nickel magnetic beads, and stored in 50 mM Tris–HCl, 500 mM NaCl and 10% glycerol (pH 8.0) buffer.

### Kinase activity assays

For the Phos-tag gel, 50 µl of kinase reactions were formulated by combining 9 µg of recombinant proteins with a kinase buffer containing 25 mM Tris–HCl (pH 8.0), 12 mM MgCl_2_ and 1 mM dithiothreitol (DTT), with varying concentrations of ATP (0, 1, 2.5 and 5 mM) (NEB, P0756S). Phosphatase reactions were prepared in accordance with the manufacturer’s instructions, utilizing a 50-µl reaction that included 9 µg of recombinant protein and 600 U of lambda protein phosphatase (NEB, P0753S). Both kinase and phosphatase reactions were incubated at 30 °C for 30 min and subsequently terminated by incubating with 4× Laemmli sample buffer at 95 °C for 5 min. Samples of 15 µl, containing 2 µg of recombinant proteins, were loaded onto a 50 µmol l^−1^ SuperSep Phos-tag gel (7.5%, FUJIFILM Wako Pure Chemical Corporation, 192-18001) and 5 µl (0.7 µg) onto NuPAGE Bis-Tris Mini Protein Gels (4–12%; Invitrogen, NP0329BOX). The Phos-tag gels were washed three times for 10 min with Tris-buffered saline with Tween 20 buffer containing 20 mM EDTA, concluding with a final wash without EDTA. The proteins were subsequently transferred to a nitrocellulose membrane using the tank method (wet) overnight at 30 V and blocked with 5% BSA. The recombinant proteins were visualized through immunoblotting using anti-6X His tag antibody, horseradish peroxiade-conjugated (Abcam, ab1187) at a 1:8,000 dilution in 5% BSA for 2 h at room temperature. Protein signals were detected using SuperSignal West Pico PLUS Chemiluminescent Substrate (Thermo Fisher Scientific, 34577) and visualized utilizing the Bio-Rad ChemiDoc MP Imaging System (Bio-Rad, 170-8280).

Autoradiography to assess kinase activity was performed commercially (Reaction Biology, Europe GmbH). For the radioactive PK assay, 50-µl kinase reactions comprising 7 µg of recombinant proteins were mixed with the kinase buffer, in the presence or absence of [γ-^33^P]-ATP. The final concentrations for the kinase reaction included 70 mM HEPES–NaOH (pH 7.5), 3 mM MgCl2, 3 mM MnCl_2_, 3 µM Na-orthovanadate, 1.2 mM DTT and 5 μM ATP/[γ-33P]-ATP (1 × 10^7 ^cpm per sample). The kinase reaction solutions were incubated at 30 °C for 1 h and subsequently halted by incubating with 20 µl lithium dodecyl sulfate sample buffer at 95 °C for 3 min. Reaction solutions (20 µl) containing 2 µg of proteins were loaded onto an SDS–polyacrylamide gel electrophoresis (PAGE) gel, initially visualized through Coomassie blue staining, and subsequently subjected to autoradiography.

### H_2_O_2_ detection

Four seedlings of the *Gb3*-complemented lines and their null lines, derived from two independent events, were infested at the two-leaf stage. The tissues from the first and second leaves were pooled and collected at 1, 4 and 8 days following infestation with greenbug biotype E. Leaf tissues were then sectioned into three to four segments and subjected to DAB staining. In brief, the sectioned leaves were stained in a DAB solution (1 mg ml^−1^) (Sigma-Aldrich, D8001), placed under vacuum for 30 min in a desiccator and then incubated overnight in the dark. Subsequently, the tissues were destained in a fixative solution at room temperature for 3 days before being transferred to a lactoglycerol solution. The stained leaf tissues were analyzed under a compound microscope (Vabiooth Biological Microscope, model VB-M002-G-US) using a 4× objective lens, with a primary focus on the areas of the leaves exhibiting notable feeding damage. The images were processed and quantified in ImageJ version 1.53 using Colour Deconvolution 2 plugin^90^. Although numerous feeding sites were distinct and easy to count, clustered sites on vulnerable plants were recorded as a single unit. The experiment was conducted twice.

### RNA sampling, library preparation and sequencing analysis

Seedlings of null and *Gb3*-complemented transgenic lines were infested at the two-leaf stage. The first and second leaf tissues were collected at 0, 1, 4 and 8 days post infestation with greenbug biotype E. Each line consisted of three biological replicates, with each replicate containing four pooled plants. Total RNA was extracted and purified using the RNeasy Plant Mini Kit (Qiagen, 74904), followed by DNase treatment using the DNA-free DNA Removal Kit (Invitrogen, AM1906). RNA quantification and quality were assessed using the Agilent RNA 6000 Nano assay (part no. 5067-1511) on the Agilent 2100 Bioanalyzer system. Illumina libraries were constructed and sequenced using the NovaSeq X Plus Series with 150-bp paired-end reads by Novogene (https://www.novogene.com/us-en/).

Quality control (QC) of raw sequenced reads was performed to remove reads with adapter contamination or low quality (*N* > 10% or *Q*-score with over 50% bases below 5). Clean reads were aligned to the Chinese Spring IWGSC version 2.1 reference genome using HISAT2 (2.2.1)^91^ and assembled using StringTie (2.2.3)^92^. Mapped reads were counted using featureCounts (2.0.6)^93^, and differential gene expression was analyzed using DESeq2 (1.42.0)^94^. Genes with |log_2_(fold change (FC))| ≥2 and adjusted *P* ≤ 0.05 were considered as differentially expressed genes. Kyoto Encyclopedia of Genes and Genomes pathway enrichment analysis was performed using the R package clusterProfiler (4.8.1)^95^. Gene Ontology enrichment analysis was performed using Triticeae-Gene Tribe^96^.

### *Gb3* expression analysis across various tissues

Seedlings from two independent *Gb3*-complemented lines were infested at the two-leaf stage with greenbug biotypes E. Leaf, stem (about 1 inch from the root base) and root tissues were individually collected and pooled from four seedlings, both from noninfested controls (day 0) and infested plants (days 1, 4 and 8). Purified RNA concentrations were quantified using a fluorescent RNA quantification kit (DeNovix, SKU: K-30000). Reverse-transcription quantitative PCR was performed using the iTaq Universal SYBR Green One-Step Kit (Bio-Rad, 8006877), incorporating 40 ng of RNA in a 10-µl reaction mixture for each sample. Quantification was carried out using an Applied Biosystems Quantstudio 3 Realtime PCR System (A28567). The expression levels of the *Gb3* gene were normalized against the housekeeping actin gene using the ΔCt method and adjusted in relation to the noninfested control of each individual tissue. In addition, the *Gb3* expression across various tissues was normalized using noninfested controls as interplate calibrators and represented in relation to the noninfested stem control. Each sample was analyzed in triplicate from two independently derived transgenic lines. The primers utilized are detailed in Supplementary Table 8.

### Metabolomic sample preparation and data analysis

The same leaf tissue samples were used for both transcriptomics and metabolomics. Lyophilized and grounded leaf tissues (50 mg) were mixed with 70% methanol with internal standards, followed by brief vortexing and centrifugation (13,500*g* for 3 min at 4 °C). The supernatant was collected and filtered through a 0.22-µm filter membrane. QC samples were prepared by pooling and homogenizing all sample extracts and run in triplicate. Sample extracts were analyzed by Metware Biotechnology (https://www.metwarebio.com/) using their standard ultraperformance liquid chromatography–tandem mass spectrometry protocols. Ultraperformance liquid chromatography was performed on an Agilent SB-C18 column (1.8 µm, 2.1 mm × 100 mm; Agilent Technologies) with a mobile phase of water containing 0.1% formic acid and acetonitrile containing 0.1% formic acid, at 0.35 ml min^−1^ and 40 °C, with a 2-µl injection. The system was coupled to an ESI-QTRAP AB6500 for multiple reaction monitoring-based targeted quantification and a TripleTOF 6600 for data-dependent tandem mass spectrometry to enable broad metabolite identification.

Data were processed in Analyst 1.6.3, with qualitative analysis against MetwareBio’s in-house database and quantitative analysis via multiple reaction monitoring on the QTRAP. Metabolite differences among sample groups were evaluated by principal component analysis using the prcomp function in R^97^. Orthogonal partial least squares-discriminant analysis (OPLS-DA) was implemented to identify differential metabolites using the OPLSR. Anal function in the R package MetaboAnalystR 1.0.1^98^. Differential metabolites were screened using two criteria. On the basis of the results of OPLS-DA (biological repetition ≥3), multivariate analysis of variable importance in projection from OPLS-DA modeling was used to preliminarily select differential metabolites from different samples. Metabolites with variable importance in projection >1 were considered significantly different between groups. Differential metabolites were further screened by combining the *P* value (when ≥2 biological replicates) or FC values from univariate analysis. Metabolites with FC ≥2 and FC ≤0.5 were considered upregulated and downregulated metabolites, respectively. The Kyoto Encyclopedia of Genes and Genomes database^99^ was utilized for the annotation and pathway enrichment analysis of differential metabolites.

### Evolutionary analysis

Protein sequences of Gb3 and its two kinase domains were queried against the protein databases in NCBI^100^ (https://blast.ncbi.nlm.nih.gov/Blast.cgi), Phytozome^101^ (https://phytozome-next.jgi.doe.gov/blast-search) and GrainGenes^102^ (https://graingenes.org/blast/) to search for orthologs (100% coverage and >95% identity) and paralogs and domain homologs (≥80% coverage and ≥70% identity). Using a PCR diagnostic marker (section below), the presence of Gb3 orthologs was also examined in Poaceae species without whole-genome sequences but known to carry greenbug R genes. Divergence times were retrieved from other studies^103–105^. Collinearity analysis was performed in Triticeae-Gene Tribe (http://wheat.cau.edu.cn/TGT/)^96^.

### Diagnostic marker development and assessment

Genomic DNA was extracted using the cetyltrimethylammonium bromide method, and primers were designed on the conserved regions to amplify *Gb3* and its orthologous genes. The marker was initially examined and validated in TAM cultivars with and without *Gb3* and subsequently applied to detect Gb3 orthologs in other species. Validation was further confirmed by PCR amplification and nanopore sequencing of full-length *Gb3* ortholog genes (Supplementary Table 8). Their genomic and protein sequences were aligned using MAFFT^106^ to detect gene structure and allelic variations. The seed sources and accession numbers of different species used in this study are listed in Supplementary Table 9.

### Statistics and reproducibility

The kinase activity assays using Phos-tag gels were repeated independently three times with similar results and revalidated once using a radioactive assay method. Gel electrophoresis confirming the presence of *Gb2* in TAM112 using its associated marker was performed independently three times with similar outcomes. Gel electrophoresis analyses of *Gb3* across different TAM lines, accessions with other *Gb*-designated genes, and other species were also repeated independently three times with similar results. All micrographs shown are representative of three independent experiments.

### Reporting summary

Further information on research design is available in the Nature Portfolio Reporting Summary linked to this article.

## Online content

Any methods, additional references, Nature Portfolio reporting summaries, source data, extended data, supplementary information, acknowledgements, peer review information; details of author contributions and competing interests; and statements of data and code availability are available at https://doi.org/10.1038/s41588-026-02754-0.

## Supplementary information

Supplementary Information Supplementary Fig. 1.

Reporting Summary

Peer Review File

Supplementary Tables 1–9 Supplementary Tables 1–9.

Supplementary Data Source data for Supplementary Fig. 1.

## Source data

Source Data Fig. 3 Unprocessed blots/gels/pictures for Fig. 3.

Source Data Fig. 4 Statistical source data for Fig. 4.

Source Data Extended Data Fig. 5 Statistical source data for Extended Data Fig. 5.

Source Data Extended Data Fig. 6 Unprocessed blots/gels/pictures for Extended Data Fig. 6.

Source Data Extended Data Fig. 9 Unprocessed blots/gels/pictures for Extended Data Fig. 9.

## Data Availability

Genomic sequences of the *Gb3* natural variations in the *Ae. tauschii* panel are available via Zenodo at https://doi.org/10.5281/zenodo.17148531(ref. ^107^). The genomic sequences of the *Gb* genes have been submitted to the NCBI GenBank and can be retrieved using the following accession nos.: PX410268 (*Gb3*), PX410269 (*Gb4*), PX410270 (*Gb7*), PX410271 (*Gb8*), PX410272 (*Gb9*), PX410273 (*Gb49921*) and PX396327 (*Gb5*). Genomic and protein sequences for Thin.J07G574000 were extracted from the *Th. intermedium* reference genome version 3.1 (https://phytozome-next.jgi.doe.gov/info/Tintermedium_v3_1), and Asha_EVM0032746 and Alon_EVM0055582 from the respective *Ae. sharonensis* (*TH02*) and *Ae. longissima* (*TL05*) reference genomes^103^. The construct sequences for Gb3 protein localization are available in NCBI GenBank under accession nos. PX389630 (ZmUbi:*mTurquoise2-Gb3*) and PX389631 (ZmUbi:*Gb3-mTurquoise2*). Constructs related to the expression for Gb3 protein domains have been deposited in NCBI GenBank with accession nos. PZ366436 (pET28a-His-MBP-Gb3-KD) and PZ366435 (pET28a-His-MBP-Gb3-PKD). The raw sequence reads for RNA sequencing are accessible in the Sequence Read Archive of NCBI (https://submit.ncbi.nlm.nih.gov/subs/sra/) under BioProject accession no. PRJNA1329330. The metabolomic profiling data are available via Zenodo at https://doi.org/10.5281/zenodo.17144600 (ref. ^108^). Source data are provided with this paper.

## Code availability

All software and codes used in this study are publicly available as described in Methods.
