# Publisher Correction: *Gb3* encodes a unique kinase fusion protein conferring greenbug resistance in wheat

**DOI:** 10.1038/s41588-026-02835-0

**Published:** 2026-10-16

**Authors:** Dhondup Lhamo, Jianqiang Shen, Genqiao Li, Toni Mohr, Roger Thilmony, Prakitchai Chotewutmontri, Han-Chang Chang, Honglian Ye, Mingcheng Luo, Shuyu Liu, Guihua Bai, Jackie Rudd, Brett F. Carver, Yong Q. Gu, Xiangyang Xu, Steven S. Xu

**Affiliations:** 1 US Department of Agriculture—Agricultural Research Service, Crop Improvement and Genetics Research Unit, Western Regional Research Center, Albany, CA USA; 2 Department of Plant and Microbial Biology, University of California, Berkeley, CA USA; 3 US Department of Agriculture—Agricultural Research Service, Peanut and Small Grains Research Unit, Stillwater, OK USA; 4 Department of Plant Sciences, University of California, Davis, CA USA; 5 Department of Soil and Crop Science, Texas A&M University, College Station, TX USA; 6 Department of Agronomy, Kansas State University, Manhattan, KS USA; 7 Texas A&M AgriLife Research, High Plains Research and Extension Center, Canyon, TX USA; 8 Department of Plant and Soil Sciences, Oklahoma State University, Stillwater, OK USA

**Keywords:** Gene expression, Genome-wide association studies, Mutagenesis, Metabolomics, Transcriptomics

Correction to: *Nature Genetics* https://doi.org/10.1038/s41588-026-02754-0, published online 23 September 2026.

In the version of the article initially published, in the “Oxylipin genes” panel of Fig. 5e, there were two “4 days” labels in the “COM” section. The right label has now been corrected to “8 days” in the HTML and PDF versions of the article.

