# Supplementary material for: Gb3 encodes a unique kinase fusion protein conferring greenbug resistance in wheat
Source: Nat Genet. 2026 Sep 23;58(10):2696–705. doi: 10.1038/s41588-026-02754-0 (PMC13649570; doi:10.1038/s41588-026-02754-0)
Supplement: Supplementary file 2 [file 41588_2026_2754_MOESM1_ESM.pdf]

nature genetics

Supplementary information https://doi.org/10.1038/s41588-026-02754-0

Gb3 encodes a unique kinase fusion protein conferring greenbug resistance in wheat

In the format provided by the authors and unedited

Supplementary Information

Supplementary Figures

Table of Contents

Supplementary Fig. 1. Quantitative RT-PCR analysis of *Gb3* expression in non-infested and infested tissues from two independent *Gb3*-complemented lines --Page 2

Additional Supplementary Files

Supplementary Tables
Supplementary_Tables_1_9.xlsx

Table of Contents

Supplementary Table 1. Resistance profiles of *Gb* genes in Poaceae family

Supplementary Table 2. Greenbug resistance screening and *Gb3* presence or absence in the *Ae. tauschii* panel

Supplementary Table 3. Candidate gene annotations within the 300-kb interval on chromosome arm 7DL of the *Ae. tauschii* reference genomes AL8/78 and T093

Supplementary Table 4. Summary of the *Gb3* haplotypes and their geographic distributions in the *Ae. tauschii* panel

Supplementary Table 5. Greenbug biotypes used for screening *Gb3*-complemented and knockout lines

Supplementary Table 6. *Gb3* ortholog search in 270 genomes of 52 different plant species of monocot

Supplementary Table 7. *Gb3* paralogs and its domain homologs in Poaceae family

Supplementary Table 8. Primers used in this study

Supplementary Table 9. Source information of the plant materials used in this study

Supplementary Data Files
Supplementary_Data.pdf

Source Data Files
Source_Data_Figure_3.pdf
Source_Data_Figure_4.xlsx
Source_Data_Extended_Data_Figure_5.xlsx
Source_Data_Extended_Data_Figure_6.pdf
Source_Data_Extended_Data_Figure_9.pdf

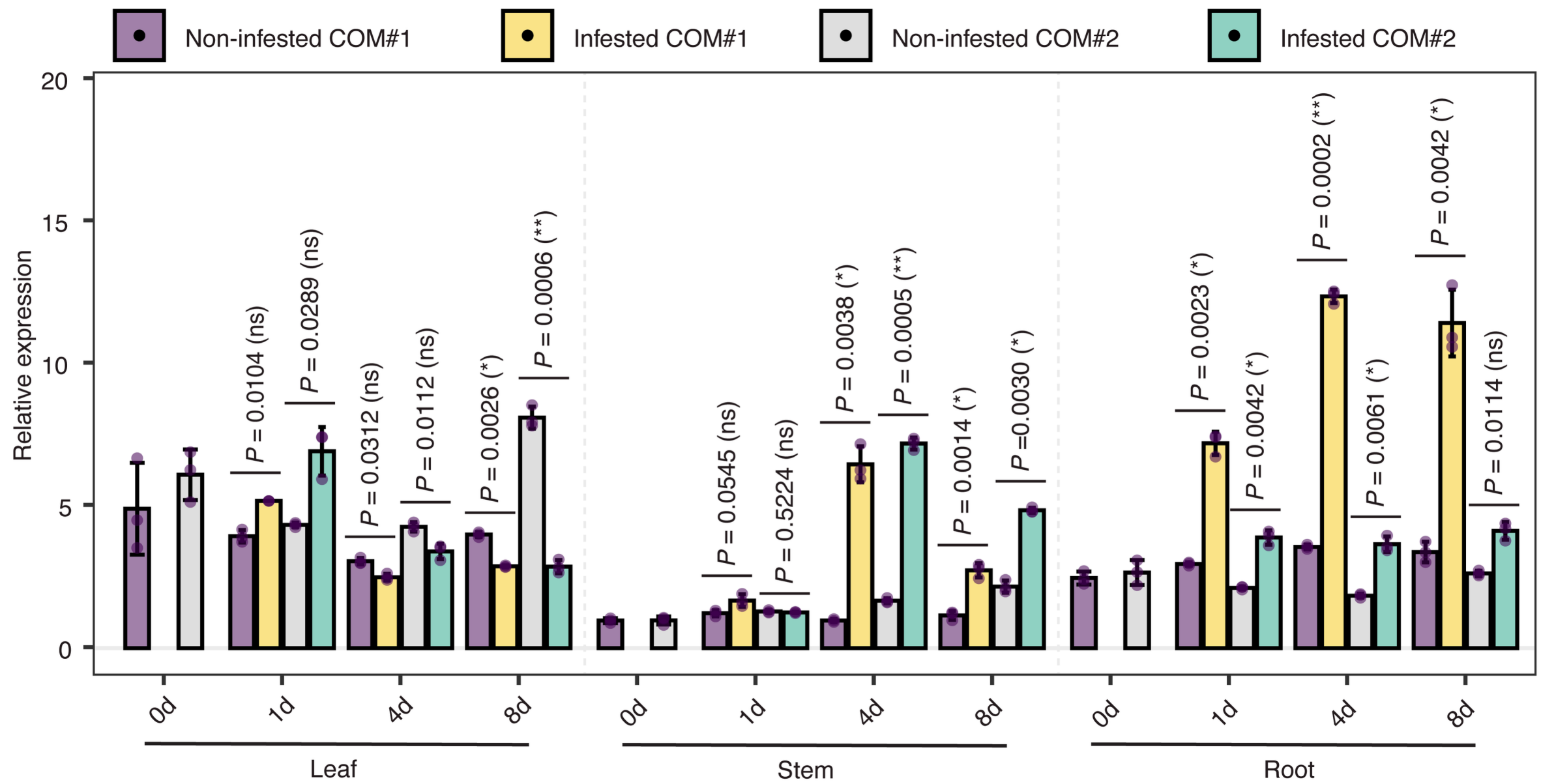

Supplementary Fig. 1 | Quantitative RT-PCR analysis of *Gb3* expression in non-infested and infested tissues from two independent *Gb3*-complemented lines. Leaf, stem, and root tissues were collected at 0, 1, 4, and 8 days post-infestation by greenbug biotype E. Gene expression levels in the various tissues were normalized and compared to the non-infested stem control. Data are presented as the mean ± standard deviation (n=3). A paired two-tailed *t*-test was performed for statistical significance, with exact *P*-values indicated. Asterisks denote significance levels: $^{*}P < 0.01$, $^{**}P < 0.001$, and $^{***}P < 0.0001$; ns = not significant.
