# Supplementary material for: Gb3 encodes a unique kinase fusion protein conferring greenbug resistance in wheat
Source: Nat Genet. 2026 Sep 23;58(10):2696–705. doi: 10.1038/s41588-026-02754-0 (PMC13649570; doi:10.1038/s41588-026-02754-0)
Supplement: Supplementary file 3 [file 41588_2026_2754_MOESM2_ESM.pdf]

Corresponding author(s): Yong-Qiang Gu, Xiangyang Xu, Steven S. Xu

Last updated by author(s): Jul 21, 2026

Reporting Summary

Nature Portfolio wishes to improve the reproducibility of the work that we publish. This form provides structure for consistency and transparency in reporting. For further information on Nature Portfolio policies, see our Editorial Policies and the Editorial Policy Checklist.

Statistics

For all statistical analyses, confirm that the following items are present in the figure legend, table legend, main text, or Methods section.

n/a	Confirmed	
☐	☒	The exact sample size (*n*) for each experimental group/condition, given as a discrete number and unit of measurement
☐	☒	A statement on whether measurements were taken from distinct samples or whether the same sample was measured repeatedly
☐	☒	The statistical test(s) used AND whether they are one- or two-sided<br>*Only common tests should be described solely by name; describe more complex techniques in the Methods section.*
☒	☐	A description of all covariates tested
☒	☐	A description of any assumptions or corrections, such as tests of normality and adjustment for multiple comparisons
☐	☒	A full description of the statistical parameters including central tendency (e.g. means) or other basic estimates (e.g. regression coefficient) AND variation (e.g. standard deviation) or associated estimates of uncertainty (e.g. confidence intervals)
☐	☒	For null hypothesis testing, the test statistic (e.g. *F*, *t*, *r*) with confidence intervals, effect sizes, degrees of freedom and *P* value noted<br>*Give P values as exact values whenever suitable.*
☒	☐	For Bayesian analysis, information on the choice of priors and Markov chain Monte Carlo settings
☒	☐	For hierarchical and complex designs, identification of the appropriate level for tests and full reporting of outcomes
☒	☐	Estimates of effect sizes (e.g. Cohen's *d*, Pearson's *r*), indicating how they were calculated

Our web collection on statistics for biologists contains articles on many of the points above.

Software and code

Policy information about availability of computer code

Data collection

Gel images for western blots were collected using SuperSignal™ West Pico PLUS Chemiluminescent Substrate (Thermo Fisher Scientific catalog #34577) and visualized utilizing the Bio-Red ChemiDocTM MP Imaging System (Bio-Rad, catalog #170-8280).

The subcellular localization of Gb3 was visualized by fluorescence imaging using a Leica TCS SP8 confocal microscope.

Hydrogen peroxide was detected via DAB staining (Sigma-Aldrich, catalog # D8001) using a Vabiooth Biological Microscope (model VB-M002-G-US).

The sequencing data for RNA-seq was performed using NovaSeq X Plus Series by Novogene (https://www.novogene.com/us-en/).

qRT-PCR for Gb3 expression was performed using the iTaq Universal SYBR Green One-Step Kit (Bio-Rad, catalog #1725150), and quantification was carried out using an Applied Biosystems QuantStudio 3 Real-Time PCR System (Thermo Fisher Scientific, catalog #A28567).

Metabolomic data were collected using high-resolution ultra-performance liquid chromatography and tandem mass spectrometry (UPLC-MS/MS) by Metware Biotechnology Inc. (Woburn, MA, https://www.metwarebio.com/).

Data analysis

SNP filtering:
Published script: https://github.com/wheatgenetics/owwc

GWAS mapping:

vcf2gwas pipeline: https://github.com/frankvogt/vcf2gwas
Manhattan plot: R package CMplot: https://github.com/YinLiLin/CMplot

Micro-collinearity:
LASTZ alignment in Geneious Prime 2024.0.7
Triticeae-Gene Tribe: http://wheat.cau.edu.cn/TGT/

Haplotype analysis:
Anchoring de novo assembled sequences: https://github.com/wheatgenetics/owwc
NCBI's BLAST+

Protein domain analysis:
InterPro v.101.0
NCBI's Conserved Domain database (CD-Search)

3D protein structure modeling:
AlphaFold v2.0

CRISPR/Cas9 editing:
gRNA design: CRISPR MultiTargeter platform
Decoding edited alleles: DECODR v3.0

Alignment of genomic and protein sequences:
MAFFT in Geneious Prime 2024.0.7

Phylogenetic analysis:
Geneious Prime 2024.0.7

RNA-seq analysis:
HISAT2 v.2.2.1
StringTie v.2.2.3
featureCounts v.2.0.6
DESeq2 v.1.42.0w

Metabolomic analysis:
Analyst v.1.6.3
R package MetaboAnalystR v.1.0.1

Gene enrichment analyses:
KEGG pathway: R package clusterProfiler v.4.8.1
GO enrichment: Triticeae-Gene Tribe: http://wheat.cau.edu.cn/TGT/

The images for hydrogen peroxide detection were processed in Image J v.1.53 using Colour Deconvulation 2 plugin.

Scatter plots, box plots, heatmaps, dot plots, violin plots, bar graphs, and statistical analyses were generated in R v.4.1.3.

The greenbug illustrations for Extended Data Fig. 10 were drawn in Adobe Fresco and compiled in PowerPoint.

For manuscripts utilizing custom algorithms or software that are central to the research but not yet described in published literature, software must be made available to editors and reviewers. We strongly encourage code deposition in a community repository (e.g. GitHub). See the Nature Portfolio guidelines for submitting code & software for further information.

Data

Policy information about availability of data

All manuscripts must include a data availability statement. This statement should provide the following information, where applicable:

- Accession codes, unique identifiers, or web links for publicly available datasets
- A description of any restrictions on data availability
- For clinical datasets or third party data, please ensure that the statement adheres to our policy

The datasets for this study are publicly available as follows:
Genomic sequence of Gb3 natural variations in the Ae. tauschii panel is available at Zenodo (https://doi.org/10.5281/zenodo.17148531).

The genomic sequences of the Gb genes have been deposited in the NCBI GenBank and are available under the accession numbers: PX410268 (Gb3), PX410269 (Gb4), PX410270 (Gb7), PX410271 (Gb8), PX410272 (Gb9), PX410273 (Gb49921) and PX396327 (Gb5).

The Gb3 subcellular localization construct sequences are accessible via GenBank (accession numbers: PX389630 and PX389631).

The Gb3 domain expression construct sequences are available in GenBank (accession numbers: PZ366436 and PZ366435).

The raw sequence reads for RNA sequencing were deposited to Sequence Read Archive (SRA) in NCBI (https://submit.ncbi.nlm.nih.gov/subs/sra/) under BioProject accession number PRJNA1329330.

The metabolomic profiling data is available at Zenodo (https://doi.org/10.5281/zenodo.17144600).

The following public databases were used for Gb3 ortholog and homolog searches:
Phytozome (https://phytozome-next.jgi.doe.gov/info/Tintermedium_v3_1)
Triticeae-Gene Tribe (TGT, http://wheat.cau.edu.cn/TGT/)
NCBI protein database (https://blast.ncbi.nlm.nih.gov/Blast.cgi)
GrainGenes (https://graingenes.org/blast/)
Triticeae-Gene Tribe (http://wheat.cau.edu.cn/TGT/)

Research involving human participants, their data, or biological material

Policy information about studies with human participants or human data. See also policy information about sex, gender (identity/presentation), and sexual orientation and race, ethnicity and racism.

Reporting on sex and gender	N/A
Reporting on race, ethnicity, or other socially relevant groupings	N/A
Population characteristics	N/A
Recruitment	N/A
Ethics oversight	N/A

Note that full information on the approval of the study protocol must also be provided in the manuscript.

Field-specific reporting

Please select the one below that is the best fit for your research. If you are not sure, read the appropriate sections before making your selection.

☒ Life sciences ☐ Behavioural & social sciences ☐ Ecological, evolutionary & environmental sciences

For a reference copy of the document with all sections, see nature.com/documents/nr-reporting-summary-flat.pdf

Life sciences study design

All studies must disclose on these points even when the disclosure is negative.

Sample size

The sample size for greenbug screenings was based on the availability of seed stock and germination rates, as well as the number of greenbug biotypes tested.

Gb3-complemented transgenics were evaluated twice at the T1 generation, using four independent lines (n=50-100 each) against greenbug biotype E. Complemented lines were tested again at the T2 generation using two stable homozygous lines with three biological replicates (n=20 each) against eight different greenbug biotypes. All tests included wheat cultivar 'TAM112' as the resistant control and Fielder as the susceptible control.

For the CRISPR/Cas9 Gb3 knockout, four independent T1 transgenic lines (n=20 each) were screened against biotype E, while three independent lines against four additional biotypes (one line had limited seed availability). For the T2 generation, six different indel events (n=10 each) were evaluated twice against three biotypes. All tests included TAM112 as the resistant control and TAM114 as the susceptible control.

The Gb3 diagnostic marker was evaluated three times in TAM lines as well as in different Poaceae species.

Aegilops species containing Gb3 allelic variants (n=5 each) were screened twice against biotype E. The tests included TAM112 as the resistant control and Fielder as the susceptible control.

The subcellular localization of Gb3 was repeated twice with two different constructs.

DAB staining was performed on leaf segments collected from 4 pooled seedlings of two independent Gb3-complemented lines (n=39-47) and their corresponding nulls (n=46-52) at three different time points. The experiment was performed twice.

Kinase activity assays using Phos-tag gels with and without ATP or phosphatase were repeated at least three times. The result was reconfirmed with radioactive-labeled ATP.

Agronomic traits were evaluated for two Gb3-complemented lines (n=108) and their corresponding nulls (n=108), using three biological replicates and two technical replicates. The experiment was repeated twice.

qRT-PCR was performed on leaf, stem, and root tissues collected from 4 pooled seedlings of two independent Gb3-complemented lines and their corresponding nulls at four different time points under infested and non-infested conditions. Three technical replicates were used for

	quantification.
Data exclusions	No data were excluded.
Replication	As indicated above, phenotypic screenings of Gb3-complemented and Gb3-knockout lines were repeated at least three different times using multiple independent lines (T1 and T2 generations) and biological replicates. Phenotypic screenings of different Aegilops species and subcellular localization were repeated twice. Genotypic analyses using the Gb3 diagnostic marker were evaluated three times. DAB staining was performed twice. Kinase activity assays using phos-tag gels were repeated three times and reconfirmed once with a radioactive assay. Agronomic trait evaluations were performed in two sets. qRT-PCR included two independent lines with three technical replicates. All repeated experiments resulted in the same conclusions.
Randomization	Greenbug infested plants were allocated in a randomized complete block design.
Blinding	Blinding was not executed during assessment because the visual differences between the resistant and susceptible groups are self-evident at both the tissue and cellular levels, precluding subjective bias.

Reporting for specific materials, systems and methods

We require information from authors about some types of materials, experimental systems and methods used in many studies. Here, indicate whether each material, system or method listed is relevant to your study. If you are not sure if a list item applies to your research, read the appropriate section before selecting a response.

Materials & experimental systems

n/a	Involved in the study	
☐	☒	Antibodies
☒	☐	Eukaryotic cell lines
☒	☐	Palaeontology and archaeology
☒	☐	Animals and other organisms
☒	☐	Clinical data
☒	☐	Dual use research of concern
☐	☒	Plants

Methods

n/a	Involved in the study	
☒	☐	ChIP-seq
☒	☐	Flow cytometry
☒	☐	MRI-based neuroimaging

Antibodies

Antibodies used	Anti-6X His tag® antibody - HRP conjugated (Abcam, ab1187)
Validation	Anti-6X His tag® antibody - HRP conjugated (Abcam, ab1187) was used in immunoblotting to detect the recombinant proteins.

Dual use research of concern

Policy information about dual use research of concern

Hazards

Could the accidental, deliberate or reckless misuse of agents or technologies generated in the work, or the application of information presented in the manuscript, pose a threat to:

No	Yes	
☒	☐	Public health
☒	☐	National security
☒	☐	Crops and/or livestock
☒	☐	Ecosystems
☒	☐	Any other significant area

Experiments of concern

Does the work involve any of these experiments of concern:

No	Yes	
☒	☐	Demonstrate how to render a vaccine ineffective
☒	☐	Confer resistance to therapeutically useful antibiotics or antiviral agents
☒	☐	Enhance the virulence of a pathogen or render a nonpathogen virulent
☒	☐	Increase transmissibility of a pathogen
☒	☐	Alter the host range of a pathogen
☒	☐	Enable evasion of diagnostic/detection modalities
☒	☐	Enable the weaponization of a biological agent or toxin
☒	☐	Any other potentially harmful combination of experiments and agents

Plants

Seed stocks	Details on seed sources for each accession or line used can be found in Supplementary Table 11. The seed stocks of Ae. tauschii panel were provided by Dr. Brande B. H. Wulff and Liz Sayers, John Innes Centre (Norwich, UK), John Raupp, Wheat Genetics Resource Center (Manhattan, KS, USA), and Dr. Dr. Harold E Bockelman from the USDA-ARS National Small Grain Collection (Aberdeen, ID, USA). Seed stocks for TAM cultivars were provided by two coauthors (Shuyu Liu and Jack Rudd); Seed stocks for other Poaceae species with and without Gb3 were obtained from USDA-ARS National Plant Germplasm System (NPGS) in Aberdeen, ID, and Pullman, WA, USA as well as from two coauthors (Guihua Bai and Xiangyang Xu).
Novel plant genotypes	The Gb3-complemented transgenics were generated in the common wheat cultivar Fielder using Agrobacterium-mediated (AGL1 strain) transformation. Four independent T1 generation and two independent stable homozygous T2 generation complemented lines were screened against greenbugs. For CRISPR/Cas9-mediated Gb3 knockout, the gRNA (GTTACTCTAGATCTACCCGG) targets exon 7 of the Gb3 gene in the common wheat cultivar TAM112 harboring Gb3. Four independent knockout lines in the T1 generation and six different indel events in the T2 generation were screened against multiple greenbug biotypes. Primers used to generate constructs for transgenics are listed in Supplementary Table 10.
Authentication	The accessions of Ae. tauschii and other Poaceae species were examined for their identity based on the botanical and plant morphological characteristics. Gb3-specific and hygromycin-specific primer sets were used to genotype TAM cultivars and transgenic lines. In addition, a PCR-based Cleaved Amplified Polymorphic Sequences (CAPS) assay targeting the SmaI recognition site, followed by amplicon Sanger sequencing of CRISPR-edited lines, was used to identify mutations at the targeted site.
