# Supplementary material for: Gb3 encodes a unique kinase fusion protein conferring greenbug resistance in wheat
Source: Nat Genet. 2026 Sep 23;58(10):2696–705. doi: 10.1038/s41588-026-02754-0 (PMC13649570; doi:10.1038/s41588-026-02754-0)
Supplement: Supplementary file 4 [file 41588_2026_2754_MOESM3_ESM.pdf]

nature portfolio

Peer Review File

Gb3 encodes a unique kinase fusion protein conferring greenbug resistance in wheat

Corresponding Author: Dr Steven Xu

Version 0:

Decision Letter:

24th Nov 2025

Dear Dr Xu,

Your Letter, "*Gb3* encodes a unique kinase fusion protein to confer broad-spectrum greenbug resistance" has now been seen by 3 referees. You will see from their comments copied below that while they find your work of considerable potential interest, they have raised quite substantial concerns that must be addressed. In light of these comments, we cannot accept the manuscript for publication, but would be interested in considering a substantially revised version that addresses these serious concerns.

We hope you will find the referees' comments useful as you decide how to proceed. If you wish to submit a substantially revised manuscript, please bear in mind that we will be reluctant to approach the referees again in the absence of major revisions.

To guide the scope of the revisions, the editors discuss the referee reports in detail within the team with a view to identifying key priorities that should be addressed in revision. In this case, all three referees note that some of the conclusions are not sufficiently supported at this stage. We particularly ask that you perform additional experiments to strengthen the conclusions and address all referee comments as thoroughly as possible with appropriate revisions. Please do not hesitate to get in touch if you would like to discuss these issues further.

If you choose to revise your manuscript taking into account all reviewer and editor comments, please highlight all changes in the manuscript text file. At this stage we will need you to upload a copy of the manuscript in MS Word .docx or similar editable format.

We are committed to providing a fair and constructive peer-review process. Do not hesitate to contact us if there are specific requests from the reviewers that you believe are technically impossible or unlikely to yield a meaningful outcome.

If revising your manuscript:

*1) Include a “Response to referees” document detailing, point-by-point, how you addressed each referee comment. If no action was taken to address a point, you must provide a compelling argument. This response will be sent back to the referees along with the revised manuscript.

*2) If you have not done so already please begin to revise your manuscript so that it conforms to our Letter format instructions, available here.
Refer also to any guidelines provided in this letter.

*3) Include a revised version of any required Reporting Summary: https://www.nature.com/documents/nr-reporting-summary.pdf
It will be available to referees (and, potentially, statisticians) to aid in their evaluation if the manuscript goes back for peer review.
A revised checklist is essential for re-review of the paper.

Please be aware of our guidelines on digital image standards.

EXTENDED DATA FIGURES

When re-submitting your manuscript, please ensure that any supplementary figures and tables that are crucial to the manuscript's conclusions are converted into Extended Data figures and tables to increase visibility of these data. Extended Data figures and tables are online-only (present in the online PDF and full-text HTML versions of the paper), peer-reviewed display items that provide essential background to the article but are not included in the main article due to space constraints. A maximum of ten Extended Data display items (figures and tables) is permitted.

You may use the link below to submit your revised manuscript and related files:

Link Redacted

Note: This URL links to your confidential home page and associated information about manuscripts you may have submitted, or that you are reviewing for us. If you wish to forward this email to co-authors, please delete the link to your homepage.

If you wish to submit a suitably revised manuscript we would hope to receive it within 6 months. If you cannot send it within this time, please let us know. We will be happy to consider your revision so long as nothing similar has been accepted for publication at Nature Genetics or published elsewhere. Should your manuscript be substantially delayed without notifying us in advance and your article is eventually published, the received date would be that of the revised, not the original, version.

Please do not hesitate to contact me if you have any questions or would like to discuss the required revisions further.

Nature Genetics is committed to improving transparency in authorship. As part of our efforts in this direction, we are now requesting that all authors identified as 'corresponding author' on published papers create and link their Open Researcher and Contributor Identifier (ORCID) with their account on the Manuscript Tracking System (MTS), prior to acceptance. ORCID helps the scientific community achieve unambiguous attribution of all scholarly contributions. You can create and link your ORCID from the home page of the MTS by clicking on 'Modify my Springer Nature account'. For more information please visit please visit www.springernature.com/orcid.

Thank you for the opportunity to review your work.

Sincerely,

Wei Li, PhD
Senior Editor
Nature Genetics
New York, NY, USA
www.nature.com/ng

Reviewers' Comments:

Reviewer #1 (Remarks to the Author):

This study represents a landmark contribution to wheat insect resistance research, addressing a long-standing gap in cloned greenbug resistance genes. The successful cloning of Gb3, the first greenbug resistance gene in wheat and one of the very few cloned insect resistance genes in cereals, is a major achievement. The work is methodologically rigorous, integrating genome-wide association mapping, pangenome analysis, functional validation (transgenic complementation and CRISPR/Cas9 knockout), evolutionary analysis, and multi-omics (transcriptomics/metabolomics) to unravel the gene function and mechanism. The identification of a unique kinase fusion protein (Gb3) and its role in oxylipin-mediated defense provides novel insights into plant-insect interactions. Additionally, the development of a diagnostic PCR marker enhances the practical utility for breeding programs, bridging basic research and applied agriculture.

Comments and Suggestions for Improving the manuscript:

1. The Gb3 encoded PK-MAP3K fusion protein is head-to-head with a NLR protein RGA5. Recent finding on wheat tandem kinase WTK3 and Sr62 revealed the indispensable functions of a genetically linked nearby NLR proteins (WTN1 and Sr62-NLR) for the fungal resistance. The allelic variation of the RGA5 need to be analyzed and validated for its possible function in Gb3 resistance.
2. For the MAP3K-like domain, test if Gb3 has kinase activity in vitro (e.g., kinase assay with recombinant protein). This validates the functional relevance of the kinase-pseudokinase configuration, which is proposed but not experimentally confirmed.
3. The omics data is rich, but the connection to Gb3's function as a kinase is still indirect.
4. The consolidation of six previously designated Gb genes (Gb3, Gb4, Gb7, Gb8, Gb9, Gb49921) into a single genetic locus

is a very important finding. However, the reasoning could be clearer for the general reader. It would be better to add a sentence to clarify this point. For example, in the results section (line 128-129): "Moreover, the gene structures and protein sequences among the six Gb genes from Ae. tauschii were identical (Fig. 3b,c), strongly suggesting they are the same gene (Gb3) that was independently identified in different genetic backgrounds and given different names. The differences in their resistance profiles are therefore likely attributed to these distinct genetic backgrounds.
5. The paper identifies 8 Gb3 haplotypes (H1-H8) with two nonsynonymous mutations (D129E, A441T) causing susceptibility. Add a summary table of haplotypes, including mutation positions (exon/domain), frequency in the Ae. tauschii panel, and resistance/susceptible phenotypes. This simplifies comparison and highlights key functional residues. Explain whether the susceptible/resistance haplotypes are rare in natural populations or associated with specific geographic/ecological contexts. This contextualizes the evolutionary significance of Gb3 conservation.
6. The agronomic traits (e.g., yield components, plant height) of Gb3-transgenic lines need to be reported to ensure resistance does not incur fitness costs—a key consideration for adoption.
7. Figure 1b, the annotated proteins for the Gb3 genomic interval could be provided to illustrate what kind of proteins are located in this genomic region.
8. Figure 2a and Extended Data Fig. 4, change WT to TAM112.
9. Extended Data Fig. 2: add labels for deletion boundaries in susceptible accessions and highlight Gb3's position relative to flanking genes. This reduces ambiguity for readers unfamiliar with the Ae. tauschii genome.
10. Extended Data Fig. 8d, include exact numbers of up/downregulated DEGs/metabolites in the figure legend or a supplementary table. This avoids forcing readers to infer values from bar graphs.

Reviewer #2 (Remarks to the Author):

This manuscript reports the cloning of Gb3, the first greenbug resistance gene cloned from wheat, which encodes a tandem kinase protein consisting of PK-like and MAP3K-like domains. The authors demonstrated that other greenbug resistance genes, including Gb4, Gb5, Gb7, Gb8, Gb9, and Gb49921, are likely to be the same as, allelic to, or orthologous with Gb3. Solid genetic evidence has been provided to validate that Gb3 confers resistance to several greenbug biotypes. The authors also explored the evolutionary history of Gb3 in Triticeae species and proposed that it likely originated from a fusion of two kinase-encoding genes, followed by gene duplication and deletion. Through transcriptomic and metabolomic analyses, the authors speculate that Gb3 confers resistance by modulating hormones and oxylipin production, thereby inducing the expression of defense genes and the accumulation of metabolites that deter greenbugs. Overall, this is a crucial study that reports the cloning of the first greenbug resistance gene from wheat. Importantly, it demonstrates that Gb3 and its allelic/orthologous Gb4/Gb5/Gb7/Gb8/Gb9/Gb49921 encode unconventional tandem kinase proteins. I have one major concern and a few minor ones. Please find them below.

Major concern:
Although transcriptomic and metabolomic analyses led the authors to conclude that Gb3-mediated aphid resistance is conferred by changes in metabolites and the expression of downstream defense genes, it is hard to judge whether these changes are the cause or the consequence of the resistance mediated by Gb3. And there is no genetic evidence to support this conclusion. Recent mechanistic studies on tandem kinases have revealed that they function in partnership with NLRs as sensor-executor pairs. In this model, the tandem kinase perceives pathogen effectors via direct binding and subsequently activates the NLR. This activation triggers cell death through NLR oligomerization and calcium influx. Greenbugs are typical piercing-sucking pests. They more closely resemble plant biotrophic pathogens and have intensive interactions with the plant, in contrast to chewing pests that primarily cause mechanical damage.In Supplementary Table 1, all listed greenbug resistance genes confer biotype-specific resistance, which implies that they may recognize different effectors from different greenbug biotypes. I suggest reconsidering the conclusions drawn from the transcriptomic and metabolomic analyses.

Minor concerns:
1. The immune response mediated by NLRs or TKPs is often accompanied by ROS production and cell death. I suggest that the authors perform DAB and trypan blue staining after greenbug infestation to determine whether Gb3 could result in ROS accumulation and cell death.
2. L58, according to the data in Supplementary Table 1, Gb3 provides "biotype-specific" resistance, rather than "broad-spectrum" resistance.
3. L61, based on the data in Supplementary Table 1, the greenbug biotype TX1 is virulent to Gb3, whereas your study found that Gb3 is effective to this biotype, how to explain it ?
4. L68, in addition to the KFP-encoding gene, what other genes are annotated within the 300-kb Gb3 interval?
5. L71, Extended Data Fig. 3a, what do the blue and red bars represent? The figure could be improved for clarity to help readers understand.
6. L82, the authors use the term "kinase fusion protein (KFP)" instead of "tandem kinase," which is acceptable. However, please state clearly that KFP consists of two kinase domains, for example use the full term "protein kinase (PK)" rather than the abbreviated "PK".
7. L116-117, the descriptions in the text and table are not clear, please clarify by adding more specific details.
8. L130-131, you cannot exclude the possibility that these Aegilops species possess other greenbug resistance genes.
9. L132-133, in Supplementary Table 8, Pm13 is not a tandem kinase but a kinase fused with an MLKL-NTD. Additionally, Lr39, which encodes a tandem kinase fused to an MSP and WD40 domain, is missing from the list. It would be good to add more discussion that compares Gb3 with published KFPs and properly cite the publications in the main text.
10. L136, in the Extended Data Fig. 6b, the authors only compared the two kinase domains of Gb3. I suggest performing a more comprehensive analysis that includes the kinase domains of all cloned KFPs.

11. L142-146, the two sentences lack a logical connection. The fact that the MAP3K-like domain of Gb3 is a pseudokinase suggests that it may function as a decoy to perceive greenbug effectors.
12. L152-172, as I have commented above, it is hard to judge whether the transcriptomic and metabolomic changes are the cause or the consequence of the resistance mediated by Gb3.
13. Methods, please describe the methods for rearing the greenbugs, infesting them onto the plants, and evaluating the subsequent plant phenotypes.

Reviewer #3 (Remarks to the Author):

The manuscript by Dhondup Lhamo et al. reports the isolation of the first greenbug resistance gene, Gb3, in wheat. Gb3 encodes a unique kinase fusion protein featuring a MAP3K-like domain. The authors demonstrate that this gene likely originated through a fusion event, followed by duplication and indel events within Triticeae. Transgenic expression of Gb3 in the susceptible wheat cultivar ‘Fielder’ conferred resistance to multiple biotypes, whereas CRISPR/Cas9 knockout of Gb3 in ‘TAM112’ rendered the plants susceptible. It is interesting to find that tandem kinases can confer insect resistance. Isolation of Gb3 helps to understand the insect resistance mechanism and provides valuable genetic resources for insect resistance breeding in wheat.
This is the first report showing that tandem kinases confer resistance to insects. I suggest that the authors provide additional data to help readers understand how Gb3 functions in greenbug resistance. The authors conclude that Gb3 likely transmits signals from greenbugs to activate defense genes (Abstract, line 30), but more experimental evidence is needed to support this conclusion.
Accessions with mutations in exon 3 (D129E) and exon 8 (A441T) became susceptible. It would be useful to clarify whether these amino acid substitutions (D129E and A441T) affect Gb3 function. It is possible that Gb3 induces a hypersensitive response.
In the proposed model of the Gb3-mediated resistance mechanism, plant hormones play a crucial role. Since SA was down-regulated here, the authors could provide more data to clarify how Gb3, plant hormones, and hormone-related genes respond to greenbug infestation.
I would also like to know in which tissues Gb3 is expressed, and whether its expression is induced by greenbug attack. Furthermore, it would be valuable to determine the type of resistance mechanism conferred by Gb3—whether it is antibiosis, antixenosis, or tolerance.
Finally, in line 23, the authors cite a review published 14 years ago. There has been considerable progress in this field since then, and more recent references should be included.

Version 1:

Decision Letter:

20th May 2026

Dear Dr Xu,

Your Letter, "*Gb3* encodes a unique kinase fusion protein to confer greenbug resistance" has now been seen by 3 referees. You will see from their comments below that while they find your work of interest, some important points are raised. We are interested in the possibility of publishing your study in Nature Genetics, but would like to consider your response to these concerns in the form of a revised manuscript before we make a final decision on publication.

We therefore invite you to revise your manuscript taking into account all reviewer and editor comments. Please highlight all changes in the manuscript text file. At this stage we will need you to upload a copy of the manuscript in MS Word .docx or similar editable format.

We are committed to providing a fair and constructive peer-review process. Do not hesitate to contact us if there are specific requests from the reviewers that you believe are technically impossible or unlikely to yield a meaningful outcome.

When revising your manuscript:

*1) Include a “Response to referees” document detailing, point-by-point, how you addressed each referee comment. If no action was taken to address a point, you must provide a compelling argument. This response will be sent back to the referees along with the revised manuscript.

*2) If you have not done so already please begin to revise your manuscript so that it conforms to our Letter format instructions, available
here.
Refer also to any guidelines provided in this letter.

*3) Include a revised version of any required Reporting Summary: https://www.nature.com/documents/nr-reporting-summary.pdf
It will be available to referees (and, potentially, statisticians) to aid in their evaluation if the manuscript goes back for peer review.

A revised checklist is essential for re-review of the paper.

Please be aware of our guidelines on digital image standards.

EXTENDED DATA FIGURES

When re-submitting your manuscript, please ensure that any supplementary figures and tables that are crucial to the manuscript's conclusions are converted into Extended Data figures and tables to increase visibility of these data. Extended Data figures and tables are online-only (present in the online PDF and full-text HTML versions of the paper), peer-reviewed display items that provide essential background to the article but are not included in the main article due to space constraints. A maximum of ten Extended Data display items (figures and tables) is permitted.

Please use the link below to submit your revised manuscript and related files:

Link Redacted

Note: This URL links to your confidential home page and associated information about manuscripts you may have submitted, or that you are reviewing for us. If you wish to forward this email to co-authors, please delete the link to your homepage.

We hope to receive your revised manuscript within four to eight weeks. If you cannot send it within this time, please let us know.

Please do not hesitate to contact me if you have any questions or would like to discuss these revisions further.

Nature Genetics is committed to improving transparency in authorship. As part of our efforts in this direction, we are now requesting that all authors identified as 'corresponding author' on published papers create and link their Open Researcher and Contributor Identifier (ORCID) with their account on the Manuscript Tracking System (MTS), prior to acceptance. ORCID helps the scientific community achieve unambiguous attribution of all scholarly contributions. You can create and link your ORCID from the home page of the MTS by clicking on 'Modify my Springer Nature account'. For more information please visit please visit www.springernature.com/orcid.

We look forward to seeing the revised manuscript and thank you for the opportunity to review your work.

Sincerely,

Wei Li, PhD
Senior Editor
Nature Genetics
www.nature.com/ng

Reviewers' Comments:

Reviewer #1 (Remarks to the Author):

The revised manuscript made significant revision based on the comments of the three reviewers. The authors performed additional experiments and provided strong new information to support their findings. I was satisfied with the scientific meaning and content of the revision. I have no further comments for the revised manuscript.

Reviewer #2 (Remarks to the Author):

The authors have addressed most of my comments from my first round of review. The overall quality of the manuscript has been significantly improved. I have only a few minor comments.
L48, Lr39 is just one WTK, the author did not name it as WTK8, then the WTK8-MSP-WD40 should be WTK-MSP-WD40.
L120, based on the Extended Data Fig. 3c, the aspartate in D129 of Gb3 is not conserved.
L233-234, "Gb3 variants across all Aegilops species confer resistance to greenbug biotype E (Extended Data Fig. 9e)", as I have commented last time. This statement is not accurate, as you cannot exclude the possibility that these Aegilops species carry other greenbug resistance genes.
L243-244, the statement "only four insect resistance genes were identified in plants", is not correct. As I know, there are quite a few brown planthopper resistance genes cloned from rice, such as Bph3, Bph6, Bph9, Bph14, Bph15, Bph29, and so on.
L272, Supplementary Table 8, it is better to present qRT-PCR results in bar graphs. All expression data should use the same control group, so that you can compare the expression levels of Gb3 in different tissues, which is more relevant with the above statement.

L307, “Furthermore, Lr9 from Ae. umbellulata and Lr58 from Ae. triuncialis are linked to”, Lr58 is not from Ae. triuncialis, but also from Ae. umbellulate. Please refer to the Lr9 cloning paper.

Reviewer #3 (Remarks to the Author):

Dhondup Lhamo's manuscript has been substantially improved. New data has been added and many sections have been rewritten.
Upon reading the revised manuscript, I realised that the author had reached a new conclusion: that Gb3 mediates antixenosis-type resistance. This conclusion is stated in the Abstract (line 32), the Summary (line 321) and the proposed model (Extended Data Fig. 10). However, I have a question regarding this conclusion. I could not find any experimental evidence in the manuscript that directly supports it.
Currently, the data are insufficient to distinguish antixenosis from antibiosis. The authors may therefore need to provide additional supporting data.

Version 2:

Decision Letter:

Our ref: NG-A70514R1

23rd Jun 2026

Dear Dr. Xu,

Thank you for submitting your revised manuscript "*Gb3* encodes a unique kinase fusion protein to confer greenbug resistance" (NG-A70514R1). It has now been seen by the original referees and their comments are below. The reviewers find that the paper has improved in revision, and therefore we'll be happy in principle to publish it in Nature Genetics, pending minor revisions to satisfy the referees' final requests and to comply with our editorial and formatting guidelines.

We are now performing detailed checks on your paper and will send you a checklist detailing our editorial and formatting requirements soon. Please do not upload the final materials and make any revisions until you receive this additional information from us.

Thank you again for your interest in Nature Genetics Please do not hesitate to contact me if you have any questions.

Sincerely,

Wei Li, PhD
Senior Editor
Nature Genetics
www.nature.com/ng

Reviewer #2 (Remarks to the Author):

The authors have addressed the previous concerns and revised the manuscript properly. I recommend accepting it for publication in Nature Genetics.

Reviewer #3 (Remarks to the Author):

The authors have addressed all my concerns. I am satisfied with the findings and quality of the manuscript.

Open Access This Peer Review File is licensed under a Creative Commons Attribution 4.0 International License, which permits use, sharing, adaptation, distribution and reproduction in any medium or format, as long as you give appropriate credit to the original author(s) and the source, provide a link to the Creative Commons license, and indicate if changes were made.
In cases where reviewers are anonymous, credit should be given to 'Anonymous Referee' and the source.
The images or other third party material in this Peer Review File are included in the article's Creative Commons license, unless indicated otherwise in a credit line to the material. If material is not included in the article's Creative Commons license and your intended use is not permitted by statutory regulation or exceeds the permitted use, you will need to obtain permission directly from the copyright holder.
To view a copy of this license, visit https://creativecommons.org/licenses/by/4.0/

Dear Dr. Li,

We appreciate your consideration of our revised manuscript, "*Gb3 encodes a unique kinase fusion protein to confer greenbug resistance*" [NG-LE70514] for publication in *Nature Genetics*. We express our gratitude to the three reviewers for their thoroughness and constructive suggestions, which have significantly enhanced our manuscript. In this revised version, we include new experiments that directly address their concerns while reinforcing our previous conclusions regarding the *Gb3*-mediated resistance mechanism.

We would like to emphasize the significance and innovations of our study:

1. *Gb3* is the first cloned and functionally validated greenbug/aphid resistance gene in wheat, filling a critical gap in crop genetics and improvement.
2. *Gb3* encodes a novel non-canonical Kinase Fusion Protein (KFP) with a unique MAP3K-like domain that confers insect resistance, setting it apart from the eleven tandem kinases (TKs) linked to resistances to various fungal diseases.
3. Introducing *Gb3* into winter and spring wheat incurs no associated fitness costs.
4. *Gb3* mediates an antixenosis-type resistance; our findings indicate that ROS and cell death are not specifically linked to this resistance, unlike typical responses to fungal pathogens.
5. Transcriptomic and metabolomic analyses elucidate the downstream mechanisms of greenbug susceptibility and *Gb3*-mediated resistance, providing insights that have not been previously reported in other resistance gene cloning studies.
6. We demonstrate that six additional known *Gb* genes (*Gb4, Gb5, Gb7, Gb8, Gb9*, and *Gb49921*) encode the same kinase fusion protein as *Gb3*.

Given these significant discoveries and comprehensive analyses, we assert that our work meets the requirements of *Nature Genetics*, especially when compared to other recently published disease resistance gene cloning papers:

DOI: https://doi.org/10.1038/s41588-023-01401-2
DOI: https://doi.org/10.1038/s41588-023-01402-1
DOI: https://doi.org/10.1038/s41588-025-02203-4
DOI: https://doi.org/10.1038/s41588-025-02208-z

Below is a summary of new experiments and results addressing the reviewers' inquiries:

1. *Fitness Cost of Gb3 Introduction*: We evaluated seven agronomic and yield traits of *Gb3*-transgenic lines versus nulls in a greenhouse setting and found no significant differences between the genotypes (Extended Data Fig. 5).

2. *Validation of Kinase-Pseudokinase Architecture*: We expressed and purified the kinase and pseudokinase domains of *Gb3* from *E. coli* and conducted two *in vitro* kinase activity assays. Both assays detected phosphorylation in the kinase domain, while the pseudokinase domain exhibited no such activity (Fig. 3).

3. *Role of NLR at the Gb3 Locus:* Because the NLR in the *Gb3* region is one of the primary candidate genes, we previously constructed triple knockouts of the *NLR* and its homologs on chromosomes 7A and 7B in the wheat variety TAM112 (*Gb3*+) background and tested their resistance against two greenbug biotypes. The resistant phenotype persisted, indicating that the *NLR* is not required for *Gb3*-mediated resistance; thus, we did not include these results in the revised manuscript.

4. *Gb3 and Hypersensitive Response:* Through DAB and trypan blue staining, we assessed ROS and cell death at multiple time points during greenbug infestation. DAB staining confirmed H_2O_2 accumulation as a

general response, not specific to resistance, while cell death was minimally observed in both resistant and susceptible lines. We report DAB staining results in Figure 4 and exclude the trypan blue findings due to a lack of significant data.

5. *Gb3 Tissue Expression Post-Infestation*: qRT-PCR analysis from leaf, stem, and root tissues showed low *Gb3* expression without infestation; however, it was induced in stems upon greenbug attack, aligning with the feeding behavior of greenbugs (Supplementary Table 8).

6. *Connecting Omics Data to Gb3-Mediated Resistance*: *Gb3*-mediated resistance primarily involves antixenosis, linked to the accumulation of oxylipins and defense metabolites. Transcriptomics indicated susceptible plants focused on managing oxidative stress rather than a targeted defense (Fig. 5).

Additionally, we performed analyses regarding *Gb3*'s geographical origin (Supplementary Table 3) and compared *Gb3* domains with other TK kinase domains (Extended Data Fig. 3) to explore the implications of natural mutations associated with susceptibility. A new discussion section expands on the mechanisms of insect/aphid and TK-mediated resistance, providing connections with *Gb3* and addressing biotype-specific resistance. Based on all these findings, we propose our current understanding of how *Gb3* mediates resistance as a non-canonical R gene (Extended Data Fig. 10).

Responses to Reviewers' Comments

We have highlighted all changes made in the manuscript, figures, and supplementary materials in yellow.

Reviewer #1 (Remarks to the Author):

This study represents a landmark contribution to wheat insect resistance research, addressing a long-standing gap in cloned greenbug resistance genes. The successful cloning of Gb3, the first greenbug resistance gene in wheat and one of the very few cloned insect resistance genes in cereals, is a major achievement. The work is methodologically rigorous, integrating genome-wide association mapping, pangenome analysis, functional validation (transgenic complementation and CRISPR/Cas9 knockout), evolutionary analysis, and multi-omics (transcriptomics/ metabolomics) to unravel the gene function and mechanism. The identification of a unique kinase fusion protein (Gb3) and its role in oxylipin-mediated defense provides novel insights into plant-insect interactions. Additionally, the development of a diagnostic PCR marker enhances the practical utility for breeding programs, bridging basic research and applied agriculture.

We are grateful to the reviewer for recognizing our study as a "landmark contribution" and a "major achievement" in the field of wheat insect resistance research. This acknowledgment is especially significant as it highlights our efforts to address a “long-standing gap” in cloned greenbug resistance genes through rigorous methodologies, effectively linking basic research with applied agricultural practices.

Comments and Suggestions for Improving the manuscript:

1. The Gb3 encoded PK-MAP3K fusion protein is head-to-head with a NLR protein RGA5. Recent finding on wheat tandem kinase WTK3 and Sr62 revealed the indispensable functions of a genetically linked

nearby NLR proteins (WTN1 and Sr62-NLR) for the fungal resistance. The allelic variation of the RGA5 need to be analyzed and validated for its possible function in Gb3 resistance.

We strongly agree with the reviewer regarding the possible involvement of the adjacent *NLR* at the *Gb3* locus in *Gb3*-mediated greenbug resistance. This *NLR* was indeed one of our candidates, and preliminary haplotype analysis supported its potential role. Chen et al. (2025) previously identified an *NLR* on chromosome 1D of Fielder with 94.5% identity with its ortholog *Sr62*^{NLR} from *Ae. sharonensis*, which supported *Sr62*^{TK} resistance (DOI: 10.1126/science.adp5034), thereby indicating functional conservation between the two *NLR* orthologs. In our study, the introduction of *Gb3* into the susceptible Fielder background conferred greenbug resistance, raising the possibility of functional conservation of the *NLR* on chromosome 7D of wheat (exhibiting 97.6% identity) with its ortholog in *Ae. tauschii*, should they be involved in *Gb3*-mediated resistance. Furthermore, *NLR* homologs located on chromosomes 7B (96.3% identity) and 7A (94.9% identity) exhibit significant protein sequence similarities to the 7D *NLR* from *Ae. tauschii*, implying potential functional redundancies among these three homologs. Consequently, before we submitted the manuscript in September 2025, we had already generated triple knockouts of these *NLR* homologs in the TAM112, which carries both *Gb3* and the 7D-*NLR* from *Ae. tauschii*, using the CRISPR/Cas9 system. T_0 transgenic plants were growing in the greenhouse at that time.

We obtained 19 T_0 transgenic plants, including four with complete knockouts across all three *NLR* homologs (designated KO#1, KO#2, KO#3, and KO#4). The different CRISPR-edited alleles from these transgenic events are detailed in the table below. We subsequently screened T_1 seeds from these four independent events against greenbug biotype E, and all triple NLR knockout lines displayed resistance levels comparable to TAM112. We retested these T_1 lines, along with three independent T_2 lines derived from KO#4 (referred to as KO#5, KO#6, and KO#7), against two distinct biotypes (E and H) as shown in the photographs below. In both instances, all T_1 and T_2 triple NLR knockouts remained resistant, suggesting that the *NLR* at the *Gb3* region and its homologs on chromosomes 7A and 7B are not required for *Gb3*-mediated resistance. Due to the negative nature of these results, we have opted not to include this data within the current manuscript.

CRISPR-edited alleles of triple NLR knockouts in T_0 and T_1 generations

Generation	NLR KO	7A	7B	7D
T_0	KO#1	del6-84, ins1/5/12	del2, ins1	del15
T_0	KO#2	del1/7	del1/4	del2/4
T_0	KO#3	del2/6	del1/2	del2-129
T_0	KO#4	del1-40, ins5/7/13	del1/302	del1
T_1	KO#5	del1	del1	del1
T_1	KO#6	del1/40	del302	del1
T_1	KO#7	del1/40	del302	del1

T_1 knockouts were derived from KO#4.

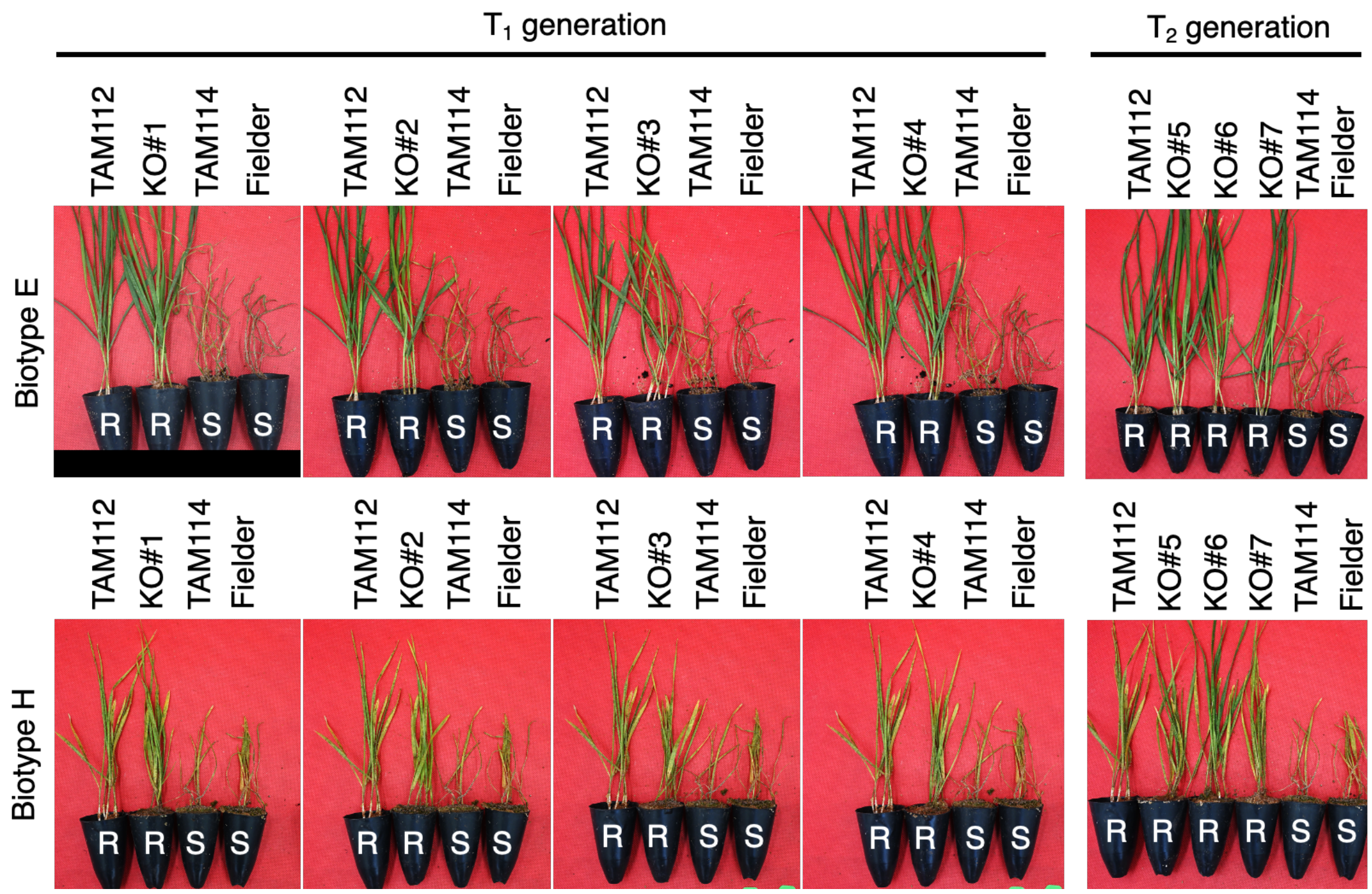

Note – The independent events were compared against the same controls for the specified biotype. Resistant control includes TAM112 (*Gb3+*), and susceptible controls include TAM114 (*Gb3-*) and Fielder (*Gb3-*).

2. For the MAP3K-like domain, test if Gb3 has kinase activity in vitro (e.g., kinase assay with recombinant protein). This validates the functional relevance of the kinase-pseudokinase configuration, which is proposed but not experimentally confirmed.

Like nearly all cloned tandem kinases (TKs), we utilized the protein sequence of Gb3 to identify critical conserved residues to propose a kinase-pseudokinase configuration. This structural hypothesis was only recently validated for RWT4, which culminated in a subsequent publication in *Nature Genetics* (Sung et al., 2025; https://doi.org/10.1038/s41588-025-02162-w), notwithstanding the earlier publications regarding the cloning of *RWT4* (Arora et al., 2023; DOI: https://doi.org/10.1038/s41477-023-01357-5) and its mechanism (Lu et al., DOI: 10.1126/science.adp5469).

In response to the reviewer's suggestion, we have successfully expressed and purified recombinant proteins comprising individual putative kinase or pseudokinase domains, each tagged with His-MBP tags in *Escherichia coli.* We investigated their kinase activities through two *in vitro* assays employing phos-tag gels with and without ATP and lambda protein phosphatase treatments, as well as autoradiography utilizing radiolabeled ATP ([γ-^{33}P]-ATP). As illustrated in **Figures 3c-e**, these kinase assays confirmed the autophosphorylation of the putative kinase domain (PK-like) utilizing phos-tag gels both with and without ATP, alongside the dephosphorylation of auto-phosphorylated proteins via phosphatase treatment, as well as active phosphorylation detected using radiolabeled ATP. In contrast, no autophosphorylation or active

phosphorylation was observed for the putative pseudokinase domain (MAP3K-like) in these two assays. In relation to **Figure 3**, we have provided a more detailed account of these results in **Lines 156–169**.

3. The omics data is rich, but the connection to Gb3's function as a kinase is still indirect.

We appreciate the reviewer for finding our omics data "rich". Our recent histological analysis of ROS (**Fig. 4**) significantly enhances our understanding of greenbug susceptibility and resistance mechanisms in plant hosts. We established a link between H_2O_2 accumulation in susceptible plants and its transcriptomic response in mitigating oxidative stress (**Figs. 4 and 5a**). H_2O_2 accumulation at greenbug feeding sites provides a means to quantify feeding sites, clearly demonstrating the deterrent effect of *Gb3* (**Fig. 4a,c**). This aligns with our earlier conclusions derived from omics data, showing that *Gb3* promotes the production of oxylipins and defense compounds exhibiting repelling and toxic effects (**Fig. 5b-e**). We have introduced a new results section for omics analyses in **Lines 184–200**. Additionally, we included a new discussion section that delineates the distinction between greenbug susceptibility and *Gb3*-mediated resistance by connecting *Gb3*'s potential role as a kinase-pseudokinase protein with cellular and phenotypic responses, as well as transcriptomic and metabolomic data in **Lines 243–301**. Based on these findings, we propose a new model of *Gb3*-mediated resistance in **Extended Data Figure 10** and summarize what additional experiments are necessary to elucidate the direct mechanism of *Gb3*-mediated resistance in **Lines 318–328**. Furthermore, we want to emphasize that elucidating the direct *Gb3*-mediated resistance is a long-term ongoing project, as evidenced by our efforts to characterize the involvement of *NLR* at the *Gb3* locus, as previously discussed.

4. The consolidation of six previously designated Gb genes (Gb3, Gb4, Gb7, Gb8, Gb9, Gb49921) into a single genetic locus is a very important finding. However, the reasoning could be clearer for the general reader. It would be better to add a sentence to clarify this point. For example, in the results section (line 128-129): "Moreover, the gene structures and protein sequences among the six Gb genes from Ae. tauschii were identical (Fig. 3b,c), strongly suggesting they are the same gene (Gb3) that was independently identified in different genetic backgrounds and given different names. The differences in their resistance profiles are therefore likely attributed to these distinct genetic backgrounds.

We thank the reviewer for acknowledging this as an "important finding," and we have incorporated your suggestion into the manuscript as indicated below or in **Lines 227-231**.

"Moreover, the gene structures and protein sequences among the six *Gb* genes from *Ae. tauschii* were identical (Figs. 6b,c), strongly suggesting that they represent the same gene (*Gb3*) that has been independently identified in various genetic backgrounds and designated with different names. The differences in their resistance profiles are, therefore, due to distinct genetic backgrounds."

5. The paper identifies 8 Gb3 haplotypes (H1-H8) with two nonsynonymous mutations (D129E, A441T) causing susceptibility. Add a summary table of haplotypes, including mutation positions (exon/domain), frequency in the Ae. tauschii panel, and resistance/susceptible phenotypes. This simplifies comparison and highlights key functional residues. Explain whether the susceptible/resistance haplotypes are rare in natural populations or associated with specific geographic/ecological contexts. This contextualizes the evolutionary significance of Gb3 conservation.

Figure 1d summarizes various haplotypes, their corresponding nucleotide and amino acid changes, and the number of accessions exhibiting these haplotypes. In response to the reviewer's suggestion, we have provided a detailed summary of haplotypes, their associated domains and phenotypes, along with their

geographic distribution in **Supplementary Table 3**. Our new analyses enable us to contextualize the evolutionary origin, conservation, and geographic spread of the functional *Gb3* haplotype (H1). We have added a new result section for *Gb3* haplotype analysis in **Lines 102-111**.

6. The agronomic traits (e.g., yield components, plant height) of Gb3-transgenic lines need to be reported to ensure resistance does not incur fitness costs—a key consideration for adoption.

Gb3 was originally identified in synthetic wheat line Largo (Joppa and Williams 1982: https://doi.org/10.2135/cropsci1982.0011183X002200040052x) in the early 1980s. It has been effectively deployed in the winter wheat cultivars across the Great Plains of the United States for over 30 years without incurring fitness costs. The first winter cultivar 'TAM110' (Lazar et al. 1997: https://doi.org/10.2135/cropsci1997.0011183X003700060055x) carrying *Gb3* from Largo was developed released at Texas in 1996. Grain yield of TAM110 was similar to that of TAM107 and the best check cultivars in rainfed trials in the western Great Plains. Its 2-year average yield in the Western Plains Regional Performance Nursery (1994 and 1995) was 6% greater than that of 'Siouxland' and 10% greater than that of 'Larned'. Its quality characteristics have been generally similar to those of check cultivars such as 'TAM101' and TAM107 (Lazar et al. 1997).

TAM112 carrying *Gb3* from TAM110, developed in Amarillo, TX in 2005, was specifically released for its exceptional grain yield potential, especially in the dryland conditions of the southern Great Plains (Rudd et al. 2014; https://doi.org/10.3198/jpr2014.03.0016crc). Furthermore, TAM204, which originated from TAM112 in 2014, consistently outperforms or matches the grain yield of popular Texas cultivars like TAM111, TAM401, and Weathermaster 135 across a variety of environments (Rudd et al. 2019; https://doi.org/10.3198/jpr2018.12.0080crc). Hence, the integration of *Gb3* into commercial wheat cultivars is expected to have no adverse effects on agronomic traits. In the introduction, we have included no associated fitness costs found in TAM (*Gb3+*) lines **in Line 68**.

As suggested, we assessed seven agronomic and yield traits of two *Gb3*-transgenic lines in comparison to their nulls, which include plant height, number of spikes per plant, spike length, spikelets per spike, seeds per spike, thousand kernel weight, and kernel weight per tray. As illustrated in **Extended Data Figure 5**, there are no significant differences in these traits between the null and *Gb3*-complemented transgenic lines. We indicated that in **Lines 135-137**.

7. Figure 1b, the annotated proteins for the Gb3 genomic interval could be provided to illustrate what kind of proteins are located in this genomic region.

We have added annotations of nearby genes located at the *Gb3* genomic interval **in Figure 1b**. Additionally, their gene locations in AL8/78 and T093, along with their annotation descriptions, are provided in **Supplementary Table 4**.

8. Figure 2a and Extended Data Fig. 4, change WT to TAM112.

As suggested, we changed WT to TAM112 in **Figure 2a and Extended Data Fig. 6c** (previously Extended Data Fig. 4).

9. Extended Data Fig. 2: add labels for deletion boundaries in susceptible accessions and highlight Gb3's

position relative to flanking genes. This reduces ambiguity for readers unfamiliar with the Ae. tauschii genome.

As suggested, deletion boundaries are added to susceptible accessions as the dashed black box and the *Gb3*'s position is highlighted in yellow in **Extended Data Figure 2**.

10. Extended Data Fig. 8d, include exact numbers of up/downregulated DEGs/metabolites in the figure legend or a supplementary table. This avoids forcing readers to infer values from bar graphs.

The exact numbers of upregulated DEGs and metabolites in null and *Gb3*-complemented lines are added in **Extended Data Figures 7f and 7g** (previously Extended Data Fig. 8d).

Reviewer #2 (Remarks to the Author):

This manuscript reports the cloning of Gb3, the first greenbug resistance gene cloned from wheat, which encodes a tandem kinase protein consisting of PK-like and MAP3K-like domains. The authors demonstrated that other greenbug resistance genes, including Gb4, Gb5, Gb7, Gb8, Gb9, and Gb49921, are likely to be the same as, allelic to, or orthologous with Gb3. Solid genetic evidence has been provided to validate that Gb3 confers resistance to several greenbug biotypes. The authors also explored the evolutionary history of Gb3 in Triticeae species and proposed that it likely originated from a fusion of two kinase-encoding genes, followed by gene duplication and deletion. Through transcriptomic and metabolomic analyses, the authors speculate that Gb3 confers resistance by modulating hormones and oxylipin production, thereby inducing the expression of defense genes and the accumulation of metabolites that deter greenbugs. Overall, this is a crucial study that reports the cloning of the first greenbug resistance gene from wheat. Importantly, it demonstrates that Gb3 and its allelic/orthologous Gb4/Gb5/Gb7/Gb8/Gb9/Gb49921 encode unconventional tandem kinase proteins. I have one major concern and a few minor ones. Please find them below.

We appreciate the reviewer's recognition of our *Gb3* functional confirmations as “solid genetic evidence” and acknowledgment of our work as a “crucial study” that identified the first non-conventional TK and its orthologs responsible for greenbug resistance.

Major concern:

1. Although transcriptomic and metabolomic analyses led the authors to conclude that Gb3-mediated aphid resistance is conferred by changes in metabolites and the expression of downstream defense genes, it is hard to judge whether these changes are the cause or the consequence of the resistance mediated by Gb3. And there is no genetic evidence to support this conclusion. Recent mechanistic studies on tandem kinases have revealed that they function in partnership with NLRs as sensor-executor pairs. In this model, the tandem kinase perceives pathogen effectors via direct binding and subsequently activates the NLR. This activation triggers cell death through NLR oligomerization and calcium influx. Greenbugs are typical piercing-sucking pests. They more closely resemble plant biotrophic pathogens and have intensive interactions with the plant, in contrast to chewing pests that primarily cause mechanical damage.

We have addressed your inquiry about transcriptomics and metabolomics in one of your minor concerns. Please refer to **section 12 below** for details. Additionally, we have provided a comprehensive explanation highlighting that *NLR* at the *Gb3* locus and its homologs on chromosomes 7A and 7B are not involved in *Gb3*-mediated resistance, as specified in our response to **comment 1 from the first reviewer**.

2. In Supplementary Table 1, all listed greenbug resistance genes confer biotype-specific resistance, which implies that they may recognize different effectors from different greenbug biotypes. I suggest reconsidering the conclusions drawn from the transcriptomic and metabolomic analyses.

We appreciate the reviewer's observation regarding the potential for different *Gb* genes to provide biotype-specific resistance. In the revised manuscript, we assert that the variations in resistance profiles among these genes arise from differences in resistance alleles or the genetic backgrounds in which the *Gb* genes are expressed. We have cited numerous other TKs with different gene designations that were subsequently validated to encode the same resistance proteins or allelic variants. In instances where differently designated genes encode proteins with identical sequences, we linked them to distinct genetic backgrounds that likely harbor additional resistance genes contributing to biotype-specific resistance. Furthermore, we acknowledge the presence of TX1-specific resistance, which cannot be solely attributed to *Gb3*. An additional resistance gene is necessary, either functioning downstream of *Gb3* or acting independently by recognizing TX1-specific effector. The specific wordings in the revised manuscript appear in **Lines 227-237 of the results section and Lines 302-317 of the discussion section.**

Minor concerns:

1. The immune response mediated by NLRs or TKPs is often accompanied by ROS production and cell death. I suggest that the authors perform DAB and trypan blue staining after greenbug infestation to determine whether Gb3 could result in ROS accumulation and cell death.

We appreciate the reviewer's suggestion to conduct DAB and trypan blue staining to investigate whether Gb3 shares a similar resistance mechanism involving ROS production and cell death, as seen with TKs conferring resistance to fungal pathogens. This experiment clearly demonstrates the distinct responses of plants to mobile insects that create new feeding sites, compared to fungal pathogens with localized infection sites. The histological analysis revealing H_2O_2 accumulation is presented in **Figure 4** and has been included in the new results section on **Lines 171-182**. These results indicate that ROS production is a general response to greenbug feeding, observed in both susceptible and resistant plants, rather than being specifically linked to resistance. In fact, susceptible plants accumulate more ROS due to the greenbug's feeding preference for them, while *Gb3* effectively deters greenbug feeding.

We also conducted trypan blue staining of *Gb3*-complemented and null lines at different timepoints on three occasions using two different protocols. Initially, we employed a gentler method that stained leaf tissues for three days, resulting in no observable cell death. Subsequently, we applied a harsher, more common method involving boiling leaf tissues in trypan blue for a few minutes and destaining with 100% ethanol until no stain remained. In contrast to DAB staining, which clearly highlights feeding sites and adjacent cells, we did not observe staining of feeding sites nor did we record a significant number of cell deaths compared to those observed for ROS. This method was repeated twice, yielding only a few cell deaths, likely artifacts from the boiling procedure. Importantly, the limited cell deaths detected were present in both resistant and susceptible tissues (as shown below), indicating that this is not a resistance-specific response mediated by *Gb3*. Furthermore, two of the four cloned insect resistance genes identified, *Mi-1* and *BPH14*, were shown to induce ROS without causing cell death (Li et al., 2006; DOI: https://doi.org/10.1094/MPMI-19-0655; and

Hu et al., 2017; https://doi.org/10.1105/tpc.17.00263). Consequently, we did not include the cell death data in the manuscript due to its lack of significance.

In the revised version, we addressed the following regarding cell death in **Line 264-268:**

"Specific aphid resistance genes, such as *Mi-1* and *Vat*, primarily confer resistance through antixenosis, while *Sm1* and *BPH14* operate via antibiosis[22,23,42,43]. Importantly, *Mi-1* and *BPH14* have been shown to trigger oxidative bursts and SA signaling without inducing cell death, a response typically associated with pathogen resistance[44,45]."

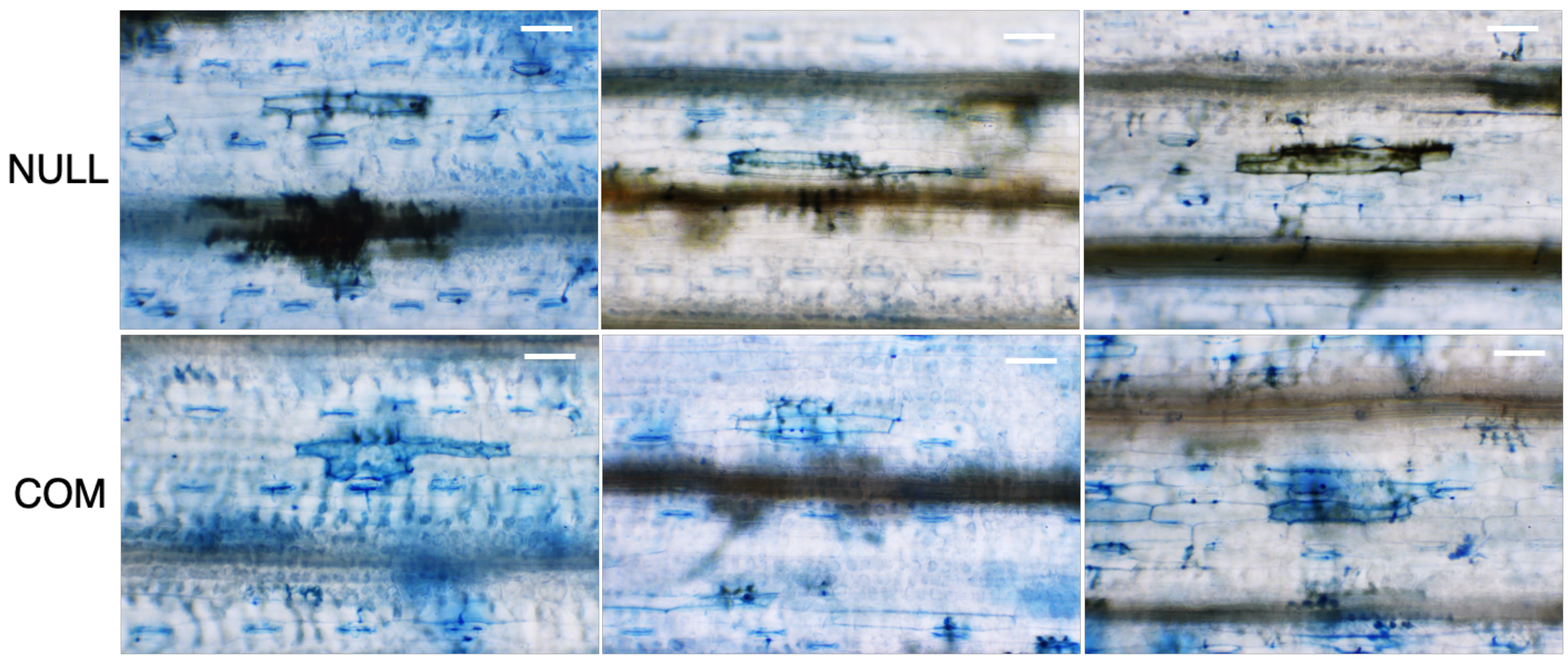

These cell deaths were observed only in leaf tissues collected form 8 days post-infestation.

2. L58, according to the data in Supplementary Table 1, Gb3 provides "biotype-specific" resistance, rather than "broad-spectrum" resistance.

In this line, we wanted to emphasize that *Gb3* provides resistance to multiple biotypes. The term "biotype-specific" does not adequately convey this, so we revised it to read:

Lines 68-69: "Remarkably, *Gb3* provides resistance to 26 of the 47 previously tested greenbug biotypes (Supplementary Table 1)."

In addition, we have removed "broad-spectrum" from both the title and the main texts.

3. L61, based on the data in Supplementary Table 1, the greenbug biotype TX1 is virulent to Gb3, whereas your study found that Gb3 is effective to this biotype, how to explain it？

We have previously addressed this inquiry in response to your 2nd major concern regarding biotype-specific resistance. To reiterate, we have identified two GWAS peaks associated with resistance to TX1, **as illustrated below and in Extended Data Figure 1e**. The predominant peak has been linked to the gene *Gb3*, while a minor peak located on chromosome 3DL may represent a novel resistance gene specific to and essential for TX1-specific resistance. Given that this locus is already exhibiting variation within the natural population of *Ae. tauschii* (**Supplemental Table 3**), it is highly probable that the same gene is absent or nonfunctional in wheat. Consequently, we hypothesize that the novel 3DL-locus gene may be

essential for *Gb3* in providing TX1-specific resistance, or it could operate independently by detecting TX1-specific effectors. This is addressed in the discussion section in **Lines 309-317**.

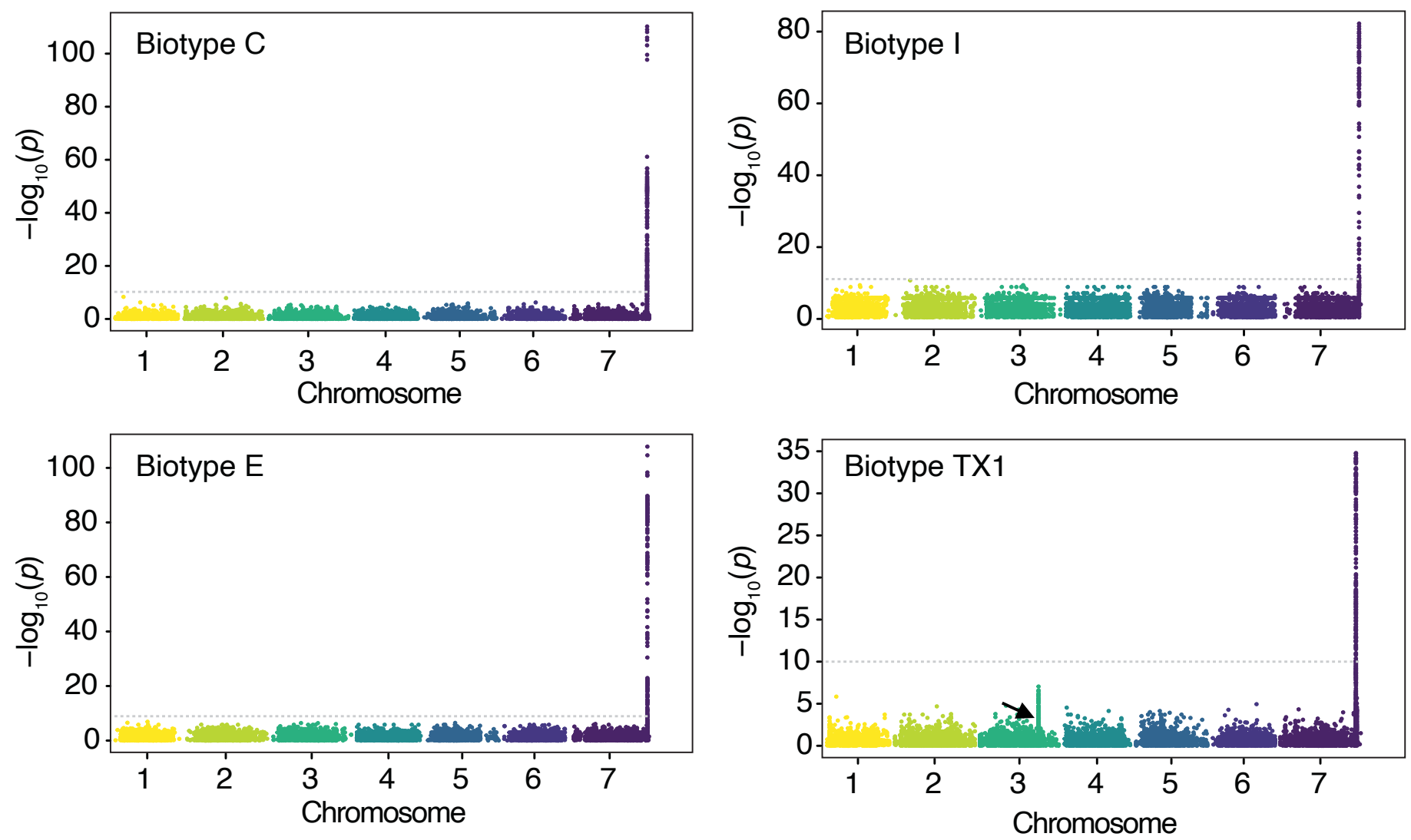

Note: A small peak specific to TX1 biotype can imply a rare resistant allele present only in a few *Ae. tauschii* accessions.

4. L68, in addition to the KFP-encoding gene, what other genes are annotated within the 300-kb Gb3 interval?

We have added annotations of nearby genes located at the *Gb3* genomic interval **in Figure 1b**. Additionally, their gene locations in *Ae. tauschii* AL8/78 and T093 genome, along with their annotation descriptions, are provided in **Supplementary Table 4**.

5. L71, Extended Data Fig. 2, what do the blue and red bars represent? The figure could be improved for clarity to help readers understand.

Extended Data Fig. 2 has been improved to include the deletion region represented by the dashed black box and the *Gb3* region highlighted in yellow. Furthermore, more details are added to the legend, including:

“Forward (blue line) and reverse (red line) sequences of *Ae. tauschii* pangenome accessions are aligned to the resistant *Gb3* donor, TA1618, by the LASTZ alignment."

“The dashed black box indicates the deletion boundaries in susceptible accessions. The yellow vertical box highlights the presence of *Gb3* in resistant pangenome sequences and its absence in susceptible pangenome sequences.”

6. L82, the authors use the term "kinase fusion protein (KFP)" instead of "tandem kinase," which is acceptable. However, please state clearly that KFP consists of two kinase domains, for example use the full term "protein kinase (PK)" rather than the abbreviated "PK".

As suggested, we modified L82 to:

Line 113-115: "*Gb3* encodes a 603-amino acid KFP with two distinct kinase domains: protein kinase(PK)-like and Mitogen Activated Protein Kinase Kinase Kinase(MAP3K)-like (**Fig. 1f**)."

7. L116-117, the descriptions in the text and table are not clear, please clarify by adding more specific details.

We apologize for any lack of clarity. Upon reviewing the previous statement (**shown below**) and the accompanying supplementary table, we have determined that the significance of this analysis is limited. It does not pertain directly to the evolution of *Gb3*, but rather to other proteins possessing a similar kinase II domain (≥80% coverage and ≥70% identity). Consequently, we have removed the statement below and its corresponding supplementary table from the revised version.

"In species lacking the kinase I domain, kinase II is fused to other protein kinase domain with distinct domains."

8. L130-131, you cannot exclude the possibility that these *Aegilops* species possess other greenbug resistance genes.

We acknowledge the reviewer's point regarding the potential presence of additional genes in various *Aegilops* species and have incorporated the following statement into the revised version in **Lines 234-237**:

"Nevertheless, different *Aegilops* species may display varying resistance profiles, as evidenced by the distinct responses of *Gb3* in *Ae. tauschii* and *Gb5* in *Ae. speltoides* (Supplementary Table 1), which can be attributed to differing genetic compositions that include additional genes for biotype-specific resistance."

9. L132-133, in Supplementary Table 8, Pm13 is not a tandem kinase but a kinase fused with an MLKL-NTD. Additionally, Lr39, which encodes a tandem kinase fused to an MSP and WD40 domain, is missing from the list. It would be good to add more discussion that compares Gb3 with published KFPs and properly cite the publications in the main text.

We are grateful to the reviewer for the correction. As all disease resistance tandem kinases are detailed in recent reviews (Li et al., 2025; doi:10.1038/s41588-026-02515-z; and Powell et al., 2026; doi:10.1038/s41588-026-02515-z), we have removed the previous Supplementary Table 8 and referenced these two review papers accordingly. Furthermore, we have included and cited the eleven cloned tandem kinases in the introduction in **Lines 41-50**, conducted a comparative analysis of their proteins and kinase domains with *Gb3* in **Lines 112-125** of the results section, and discussed the potential functional roles of *Gb3* based on the recently elucidated mechanisms for other tandem kinases as described in **Lines 248-259**.

10. L136, in the Extended Data Fig. 6b, the authors only compared the two kinase domains of Gb3. I suggest performing a more comprehensive analysis that includes the kinase domains of all cloned KFPs.

Given that *Gb3* possesses dual kinase domains, it is anticipated to exhibit greater structural and functional similarity to the eleven cloned TKs in mediating resistance compared to other KFPs, which incorporate other domains with distinct functions. Consequently, we have included eleven cloned TKs for comparison

with *Gb3*, as illustrated in **Extended Data Figure 3**, which is already comprehensive. The analysis is described in **Lines 115-125** of the results section.

11. L142-146, the two sentences lack a logical connection. The fact that the MAP3K-like domain of Gb3 is a pseudokinase suggests that it may function as a decoy to perceive greenbug effectors.

We apologize that our previous version of manuscript was observing MAP3K-like in the light of insect response, while it functionally likely resembles more like TKs. Therefore, as suggested, we have made changes to incorporate the potential role of *Gb3* as kinase-pseudokinase protein as a putative intracellular immunoreceptor in **Lines 248-259**.

12. L152-172, as I have commented above, it is hard to judge whether the transcriptomic and metabolomic changes are the cause or the consequence of the resistance mediated by Gb3.

We recognize the reviewer's feedback regarding the initial ambiguity surrounding the proposed mechanism. In response, our recent histological analysis of ROS (**Fig. 4**) significantly enhances our understanding of greenbug susceptibility and resistance mechanisms in plant hosts. We established a link between H_2O_2 accumulation in susceptible plants and its transcriptomic response in mitigating oxidative stress (**Fig. 5a**). H_2O_2 accumulation at greenbug feeding sites provides a means to quantify feeding sites, clearly demonstrating the deterrent effect of *Gb3* (**Fig. 4a,c**). This aligns with our earlier conclusions derived from omics data, showing that *Gb3* promotes the production of oxylipins and defense compounds exhibiting repelling and toxic effects (**Fig. 5b-e**). We have introduced a new results section for omics analyses in **Line 184-200**. Additionally, we included a new discussion section that delineates the distinction between greenbug susceptibility and *Gb3*-mediated resistance by connecting *Gb3*'s potential role as a kinase-pseudokinase protein with cellular and phenotypic responses, as well as transcriptomic and metabolomic data in **Lines 243-301**. Based on these findings, we propose a new model of *Gb3*-mediated resistance in **Extended Data Figure 10** and summarize what future studies are necessary to elucidate the direct mechanism of *Gb3*-mediated resistance in **Lines 318-328**.

13. Methods, please describe the methods for rearing the greenbugs, infesting them onto the plants, and evaluating the subsequent plant phenotypes.

We have incorporated additional details on greenbug rearing, infestation and phenotypic evaluations in **Lines 392-407** of the methods section.

Reviewer #3 (Remarks to the Author):

The manuscript by Dhondup Lhamo et al. reports the isolation of the first greenbug resistance gene, Gb3, in wheat. Gb3 encodes a unique kinase fusion protein featuring a MAP3K-like domain. The authors demonstrate that this gene likely originated through a fusion event, followed by duplication and indel events within Triticeae. Transgenic expression of Gb3 in the susceptible wheat cultivar ‘Fielder’ conferred resistance to multiple biotypes, whereas CRISPR/Cas9 knockout of Gb3 in ‘TAM112’ rendered the plants susceptible. It is interesting to find that tandem kinases can confer insect resistance. Isolation of Gb3 helps

to understand the insect resistance mechanism and provides valuable genetic resources for insect resistance breeding in wheat.

We acknowledge the reviewer's recognition of *Gb3*'s importance as KFP in providing insect resistance and its value as a genetic resource for breeders and understanding the mechanisms of insect resistance.

This is the first report showing that tandem kinases confer resistance to insects. I suggest that the authors provide additional data to help readers understand how Gb3 functions in greenbug resistance. The authors conclude that Gb3 likely transmits signals from greenbugs to activate defense genes (Abstract, line 30), but more experimental evidence is needed to support this conclusion.

We apologize if our terminology regarding "activate defense genes" deviates from its traditional usage in the context of pathogen resistance. We aimed to clarify that *Gb3* activates the pathway genes involved in the biosynthesis of oxylipins and defense-related secondary metabolites, such as terpenoids. This conclusion is substantiated by the transcriptomic and metabolomic analyses presented in **Figure 5**. The oxylipins and defense metabolites are recognized for their insect repellent and toxic properties, which we elaborate on in the discussion section. Our new histological analysis (**Fig. 4**) demonstrates that greenbugs feed less on *Gb3*-resistant plants compared to susceptible ones, strongly indicating *Gb3*'s role in deterrence through these defense metabolites. Additionally, our data highlights excessive ROS accumulation at the feeding sites of susceptible plants, with our transcriptomic analysis revealing that susceptible plants initiate responses to mitigate oxidative stress rather than employing defense strategies (**Figs. 4 and 5a**). Consequently, we revised the conclusion in **Lines 30-33** of the abstract as follows:

"Comprehensive analyses indicate that greenbugs preferentially feed on susceptible plants, inducing a prolonged oxidative stress response. Conversely, resistant plants expressing *Gb3* mediate antixenosis-type resistance by producing oxylipins and other defensive compounds."

Accessions with mutations in exon 3 (D129E) and exon 8 (A441T) became susceptible. It would be useful to clarify whether these amino acid substitutions (D129E and A441T) affect Gb3 function. It is possible that Gb3 induces a hypersensitive response.

We present these two natural mutations exclusively within the context of haplotype analysis; our intention is not to delve into the specific effects of these mutations on the function of *Gb3*. At this time, we can conclude that these represent the only nonsynonymous mutations in *Gb3* that confer susceptibility to all four biotypes. Furthermore, we conducted more thorough comparisons of these *Gb3* mutations with EMS-induced mutations of eleven cloned TKs located around the same residues, as illustrated in **Extended Data Fig. 3c**. Our findings are indicated in **Lines 112-125** of the results section. It is also noteworthy that other cloned TKs identifying non-synonymous EMS mutations leading to disease susceptibility have not elaborated on how these mutations affect TK function. While speculation may arise, we assert that the two mutations are unlikely to impact kinase activities; A441T is located on a pseudokinase that lacks kinase activity (**Fig. 3c-e**), and D129E is positioned 15 amino acids away from A441T when overlapping the two domains, indicating they may play a role in protein-protein interactions with an effector or a downstream target.

Regarding hypersensitive response, **Figure 4** clearly demonstrates that ROS production is a general response to greenbug feeding and is not specifically linked to *Gb3*-mediated resistance. Additionally, our cell death assays did not reveal any response related to either greenbug susceptibility or resistance. For

further details on cell death assay, please refer to our response to **comment 1 of the 2nd reviewer's minor concerns**. These studies clearly differentiate between plant resistance to insects and pathogens.

In the proposed model of the Gb3-mediated resistance mechanism, plant hormones play a crucial role. Since SA was down-regulated here, the authors could provide more data to clarify how Gb3, plant hormones, and hormone-related genes respond to greenbug infestation.

Hormonal crosstalk plays an important role in *Gb3*-mediated resistance especially in systemic signaling; however, our primary focus is not on the intricate details of these hormonal interactions in response to greenbug infestation. Instead, we aim to clarify the fundamental biological processes and metabolites that are significantly affected in *Gb3*-resistant plants compared to susceptible ones. Transcriptomic and metabolomic analyses highlight the crucial involvement of oxylipin biosynthesis (including OPDA, KODA and JA) genes and metabolites in facilitating *Gb3*-mediated resistance, as illustrated in **Figure 5**. We recognize the reviewer's feedback regarding the initial ambiguity surrounding the proposed mechanism. We request the reviewer to have a look at the **new discussion section** we added, which explains how *Gb3* may function in greenbug resistance, also summarized in our proposed model in **Extended Data Figure 10**. Additionally, we outline in the conclusion the further experiments needed to clarify direct *Gb3*-mediated resistance in **Line 323-328**.

I would also like to know in which tissues Gb3 is expressed, and whether its expression is induced by greenbug attack. Furthermore, it would be valuable to determine the type of resistance mechanism conferred by Gb3—whether it is antibiosis, antixenosis, or tolerance.

As suggested by the reviewer, we performed the *Gb3* expression analysis at four different timepoints in both non-infested and infested tissues of leaves, stems, and roots of two independent *Gb3*-complemented lines and their corresponding nulls (**Supplementary Table 8**). *Gb3*, like many other R genes, is lowly expressed in non-infested tissues. However, upon greenbug attack, its expression is induced in the stem tissues but not in the leaves. This expression pattern aligns with the type of resistance provided by *Gb3*, which is predominantly antixenosis (Weng et al., 2004; https://doi.org/10.1603/0022-0493-97.2.654). The Weng et al. (2004) research indicates that greenbug biotype E primarily feeds on the stems of susceptible plants while preferring the leaves of resistant plants. This feeding avoidance behavior toward the stems of resistant plants likely occurs because *Gb3* is most highly expressed in that infested tissue, causing greenbugs to favor the leaves where *Gb3* is not induced. These details are added in **Lines 260-273** of the discussion section.

Finally, in line 23, the authors cite a review published 14 years ago. There has been considerable progress in this field since then, and more recent references should be included.

We appreciate the reviewer for highlighting that the cited review was published 14 years ago. In accordance with our revised manuscript's formatting as an article, we have omitted that citation from the abstract. Given the absence of a current review paper addressing the number of cloned insect R genes, we have conducted extensive literature searches to compile as many cloned insect R genes as possible across various plant species. Consequently, we have individually referenced the four insect R genes that have been cloned to date in the introduction (**Line 53**) and provided additional information in **Lines 243-247** and **Lines 264-268** of the discussion section. Of these, *Mi-1*, *Vat*, and *BPH14* were cloned over a decade ago, while *Sm1* was

cloned in 2020, which highlights the limited progress in the cloning of R genes for resistance to insects. Also, we have added more up-to-date references in the introduction and discussion sections in relation to pathogen and insect R genes.

Dear Dr. Li,

We appreciate your consideration of our revised manuscript, "*Gb3 encodes a unique kinase fusion protein to confer greenbug resistance*" [NG-LE70514R] for publication as an article in *Nature Genetics*. We sincerely thank all three reviewers for their time, attention to detail, and valuable suggestions, which have significantly helped refine this manuscript.

Reviewers' Comments:

We have highlighted all changes made in the manuscript, figures, and supplementary materials in yellow.

Reviewer #1 (Remarks to the Author):

The revised manuscript made significant revision based on the comments of the three reviewers. The authors performed additional experiments and provided strong new information to support their findings. I was satisfied with the scientific meaning and content of the revision. I have no further comments for the revised manuscript.

Thank you for acknowledging the new experimental data and recognizing it as compelling evidence for our findings. We appreciate your time and constructive feedback.

Reviewer #2 (Remarks to the Author):

The authors have addressed most of my comments from my first round of review. The overall quality of the manuscript has been significantly improved. I have only a few minor comments.

Thank you for all the corrections and constructive feedback. Below are the changes made in response to your minor comments.

L48, Lr39 is just one WTK, the author did not name it as WTK8, then the WTK8-MSP-WD40 should be WTK-MSP-WD40.

The correction has been noted:
L48: "*Lr39* (*WTK8-MSP-WD40*)" is changed to "*Lr39* (*WTK-MSP-WD40*)"

L120, based on the Extended Data Fig. 3c, the aspartate in D129 of Gb3 is not conserved.

We apologize for any confusion caused by the previous statement. We have revised it to:
L119-120: "We found that the aspartate in D129 of *Gb3* is present in Kinase I of *Lr9* and *Pm57* and the alanine in A441 of *Gb3* is conserved within both kinase domains of *Yr15* and *Sr60*."

L233-234, "Gb3 variants across all Aegilops species confer resistance to greenbug biotype E (Extended Data Fig. 9e)", as I have commented last time. This statement is not accurate, as you cannot exclude the possibility that these Aegilops species carry other greenbug resistance genes.

We have revised the previous statement to acknowledge that *Aegilops* species may carry additional greenbug resistance genes. The updated statement now reads:

L233-L235: "The *Aegilops* accessions carrying these alleles show resistance to greenbug biotype E (Extended Data Fig. 9e), which can be attributed to either *Gb3* allelic variants or other novel resistance genes present in those species."

L243-244, the statement "only four insect resistance genes were identified in plants", is not correct. As I know, there are quite a few brown planthopper resistance genes cloned from rice, such as Bph3, Bph6, Bph9, Bph14, Bph15, Bph29, and so on.

We appreciate the reviewer for highlighting the additional cloned brown planthopper resistance genes. Following a thorough review of the literature, we have identified ten R insect resistance genes across various plant species, seven of which are specific to the brown planthopper. The *BPH15* locus consists of two genes, of which the cloned gene is *BPH3*, which comprises a cluster of three *OsLecRK* genes. Therefore, we report on *BPH3* rather than *BPH15* below.

We have revised the introduction as follows:
L52: "In contrast, the progress in the cloning of insect resistance genes in wheat has been markedly slower, with only one R gene identified in wheat and a total of ten across all plant species[20–29]."

Furthermore, we incorporated all *BPH* genes into the discussion section:
L245-252: "These genes include *Mi-1* in wild tomato and *Vat* in melon for aphid resistance[22,49], *Sm1* in wheat for midge resistance[20], and *BPH3, BPH6, BPH9, BPH14, BPH29, BPH30* and *BPH32* in rice for resistance against the brown planthopper[23–29,50,51]. Among these, *Mi-1, Vat, BPH9, BPH14*, and *Sm1* encode NLR proteins, with *BPH9* featuring two NBS domains and *Sm1* additionally containing kinase and MSP domains[20,22–24,49]. *BPH3* includes a cluster of three PM-localized lectin receptor kinases[29], while the remaining *BPH* genes do not encode typical R proteins. These include *BPH6*, which encodes an uncharacterized exocyst-localized protein[28]; *BPH30*, an endomembrane protein with two leucine-rich domains[27]; *BPH32*, a PM-localized protein with a short consensus repeat domain[25]; and *BPH29*, a nuclear-localized protein containing a B3 domain[26]."

L271-272: "Aphid resistance genes such as *Mi-1* and *Vat*, as well as brown planthopper resistance genes like *BPH6* and *BPH9*, confer both antixenosis and antibiosis[22,24,28,55,56], while *Sm1*, *BPH14* and *BPH32* operate via antibiosis[23,25,48]."

L272, Supplementary Table 8, it is better to present qRT-PCR results in bar graphs. All expression data should use the same control group, so that you can compare the expression levels of Gb3 in different tissues, which is more relevant with the above statement.

We have removed the previous Supplementary Table 8 as per the reviewer's suggestion and instead present the qRT-PCR data for *Gb3* expression in various tissues as bar graphs in **Supplementary Fig. 1**. The analysis of *Gb3* expression in leaf, stem, and root tissues was conducted on three separate PCR plates, with non-infested controls from these three tissues included in one of the plates to serve as inter-plate calibrators. This time, we utilize these inter-plate calibrators, which were omitted in the previous analyses, to normalize and compare *Gb3* gene expression levels across the three tissues relative to the non-infested leaf control.

We have revised the statement below to reflect the low levels of *Gb3* expression in the leaves:
L277-278: "This preference results from the induction of *Gb3* expression in the stem during infestation, while expression remains low in the leaves (Supplementary Fig. 1)."

Furthermore, the methods section has been updated accordingly:

L534-536: "Additionally, the *Gb3* expression across various tissues was normalized using non-infested controls as inter-plate calibrators and represented in relation to the non-infested leaf control."

L307, "Furthermore, Lr9 from Ae. umbellulata and Lr58 from Ae. triuncialis are linked to", Lr58 is not from Ae. triuncialis, but also from Ae. umbellulate. Please refer to the Lr9 cloning paper.

The correction has been noted:
L311-313: "Similar trends are observed among other TK genes; for instance, *Yr15, YrG303*, and *YrH52* from wild emmer wheat all encode the same WTK1 protein[77], while *Lr9* and *Lr58* encode the same WTK6-vWA from *Ae. umbellulata*[13]."

Reviewer #3 (Remarks to the Author):

Dhondup Lhamo's manuscript has been substantially improved. New data has been added and many sections have been rewritten. Upon reading the revised manuscript, I realised that the author had reached a new conclusion: that Gb3 mediates antixenosis-type resistance. This conclusion is stated in the Abstract (line 32), the Summary (line 321) and the proposed model (Extended Data Fig. 10). However, I have a question regarding this conclusion. I could not find any experimental evidence in the manuscript that directly supports it. Currently, the data are insufficient to distinguish antixenosis from antibiosis. The authors may therefore need to provide additional supporting data.

Weng et al. (2004) conducted a comprehensive antixenosis test of near isogenic lines, comparing those with and without *Gb3*, and conclusively demonstrated that greenbugs strongly prefer susceptible lines over the resistant *Gb3* line (DOI: https://doi.org/10.1093/jee/97.2.654). They also revealed the potential presence of an antibiosis effect in the resistant *Gb3* line, based on the observed fluctuation in greenbug population density with a multipeak curve over prolonged infestation periods. Our histological analysis of resistant lines further confirms the deterrent effect of *Gb3* on greenbugs, as evidenced by a reduced number of feeding sites (**Fig. 4c**). Resistant plants containing *Gb3* produce various metabolites that can have both repelling and insecticidal properties (**Fig. 5c-e**). Consequently, we acknowledge the reviewer's point that antixenosis and antibiosis resistance cannot be distinctly differentiated and both mechanisms are likely involved. Accordingly, we have revised "antixenosis-type" to "greenbug" resistance in both the abstract (**line 32**) and the summary (**line 327**) of the proposed model (**Extended Data Fig. 10**). Furthermore, we have included a discussion acknowledging that *Gb3* provides both antixenosis and antibiosis resistance (**line 275**).
