# Supplementary material for: Gb3 encodes a unique kinase fusion protein conferring greenbug resistance in wheat
Source: Nat Genet. 2026 Sep 23;58(10):2696–705. doi: 10.1038/s41588-026-02754-0 (PMC13649570; doi:10.1038/s41588-026-02754-0)
Supplement: Supplementary file 5 [file 41588_2026_2754_MOESM5_ESM.pdf]

Tissue	Line	Days post-infestation	Rep1	Rep2	Rep3
Leaf	Non-infested COM#1	0d	6.92	3.65	4.66
Leaf	Non-infested COM#1	1d	3.87	4.32	4.04
Leaf	Non-infested COM#1	4d	3.30	3.09	3.11
Leaf	Non-infested COM#1	8d	4.22	4.04	4.15
Leaf	Infested COM#1	1d	5.36	5.36	5.36
Leaf	Infested COM#1	4d	2.55	2.48	2.72
Leaf	Infested COM#1	8d	2.95	2.97	3.02
Leaf	Non-infested COM#2	0d	6.49	7.14	5.32
Leaf	Non-infested COM#2	1d	4.50	4.56	4.40
Leaf	Non-infested COM#2	4d	4.58	4.43	4.24
Leaf	Non-infested COM#2	8d	8.10	8.85	8.23
Leaf	Infested COM#2	1d	7.69	7.67	6.15
Leaf	Infested COM#2	4d	3.66	3.71	3.20
Leaf	Infested COM#2	8d	2.93	3.23	2.75
Stem	Non-infested COM#1	0d	1.09	0.91	1.01
Stem	Non-infested COM#1	1d	1.15	1.37	1.29
Stem	Non-infested COM#1	4d	0.95	1.00	1.06
Stem	Non-infested COM#1	8d	1.01	1.26	1.30
Stem	Infested COM#1	1d	1.53	1.69	1.98
Stem	Infested COM#1	4d	6.17	6.48	7.43
Stem	Infested COM#1	8d	2.54	2.92	3.04
Stem	Non-infested COM#2	0d	1.11	1.07	0.84
Stem	Non-infested COM#2	1d	1.32	1.39	1.29
Stem	Non-infested COM#2	4d	1.83	1.69	1.66
Stem	Non-infested COM#2	8d	2.49	2.17	2.06
Stem	Infested COM#2	1d	1.32	1.27	1.31
Stem	Infested COM#2	4d	7.51	7.21	7.63
Stem	Infested COM#2	8d	4.96	5.13	4.97
Root	Non-infested COM#1	0d	2.34	2.51	2.81
Root	Non-infested COM#1	1d	3.00	3.12	3.08
Root	Non-infested COM#1	4d	3.61	3.77	3.68
Root	Non-infested COM#1	8d	3.49	3.13	3.88
Root	Infested COM#1	1d	6.97	7.72	7.68
Root	Infested COM#1	4d	12.90	12.99	12.53
Root	Infested COM#1	8d	10.97	11.31	13.22
Root	Non-infested COM#2	0d	3.20	2.29	2.77
Root	Non-infested COM#2	1d	2.13	2.22	2.23
Root	Non-infested COM#2	4d	1.93	1.97	1.83
Root	Non-infested COM#2	8d	2.64	2.83	2.70
Root	Infested COM#2	1d	3.74	4.10	4.25
Root	Infested COM#2	4d	3.56	4.10	3.69
Root	Infested COM#2	8d	4.36	4.54	3.91
