# Supplementary material for: Gb3 encodes a unique kinase fusion protein conferring greenbug resistance in wheat
Source: Nat Genet. 2026 Sep 23;58(10):2696–705. doi: 10.1038/s41588-026-02754-0 (PMC13649570; doi:10.1038/s41588-026-02754-0)
Supplement: Supplementary file 6 [file 41588_2026_2754_MOESM6_ESM.pdf]

Fig. 3

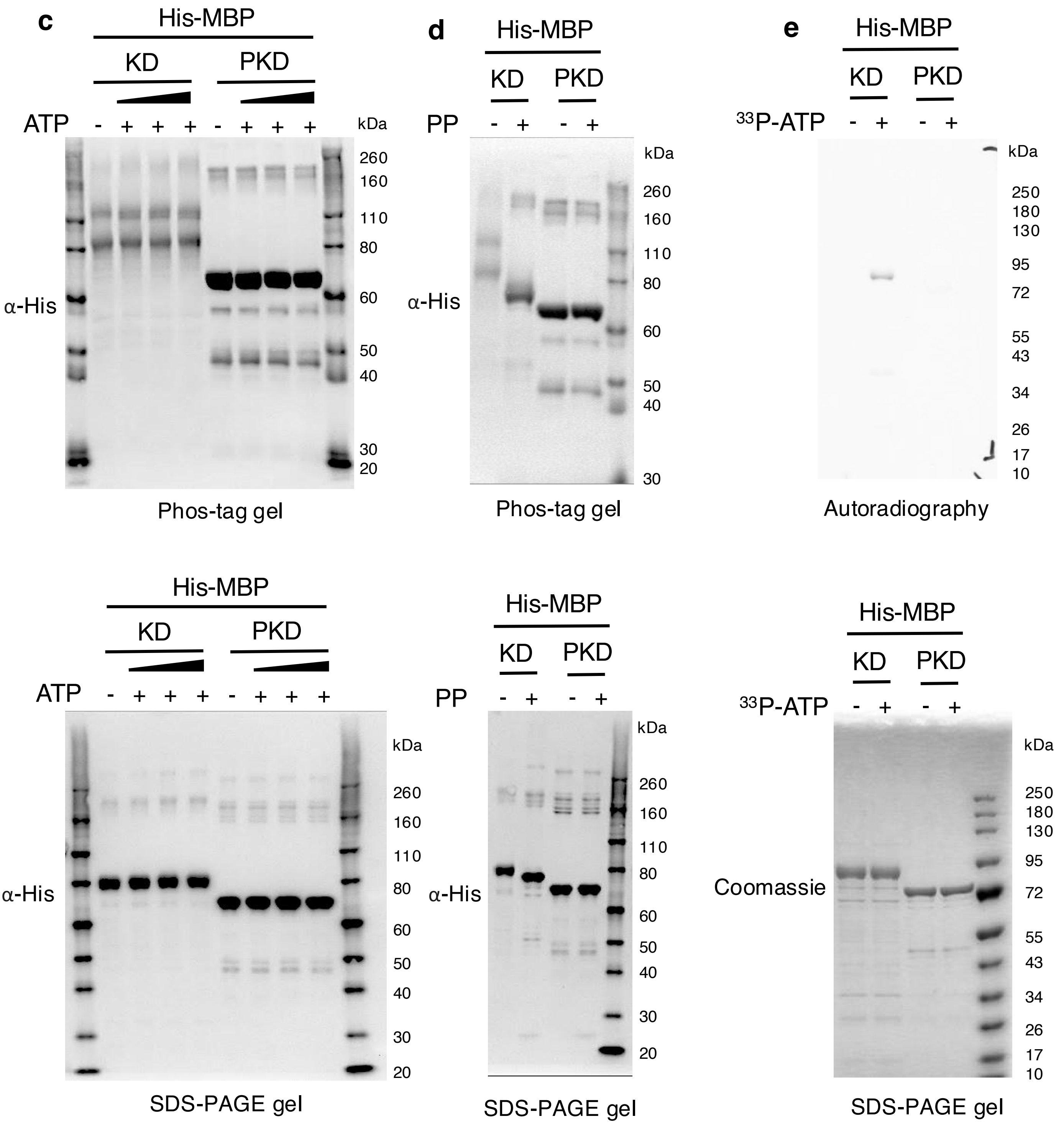

c
His-MBP
KD
PKD
ATP - + + + - + + +
kDa
260
160
110
80
60
50
40
30
20
α-His
Phos-tag gel
d
His-MBP
KD
PKD
PP - + - +
kDa
260
160
110
80
60
50
40
30
α-His
Phos-tag gel
e
His-MBP
KD
PKD
33P-ATP - + - +
kDa
250
180
130
95
72
55
43
34
26
17
10
Autoradiography
His-MBP
KD
PKD
ATP - + + + - + + +
kDa
260
160
110
80
60
50
40
30
20
α-His
SDS-PAGE gel
His-MBP
KD
PKD
PP - + - +
kDa
260
160
110
80
60
50
40
30
20
α-His
SDS-PAGE gel
His-MBP
KD
PKD
33P-ATP - + - +
kDa
250
180
130
95
72
55
43
34
26
17
10
Coomassie
SDS-PAGE gel
