# Supplementary material for: Gb3 encodes a unique kinase fusion protein conferring greenbug resistance in wheat
Source: Nat Genet. 2026 Sep 23;58(10):2696–705. doi: 10.1038/s41588-026-02754-0 (PMC13649570; doi:10.1038/s41588-026-02754-0)

Extended Data Fig. 6

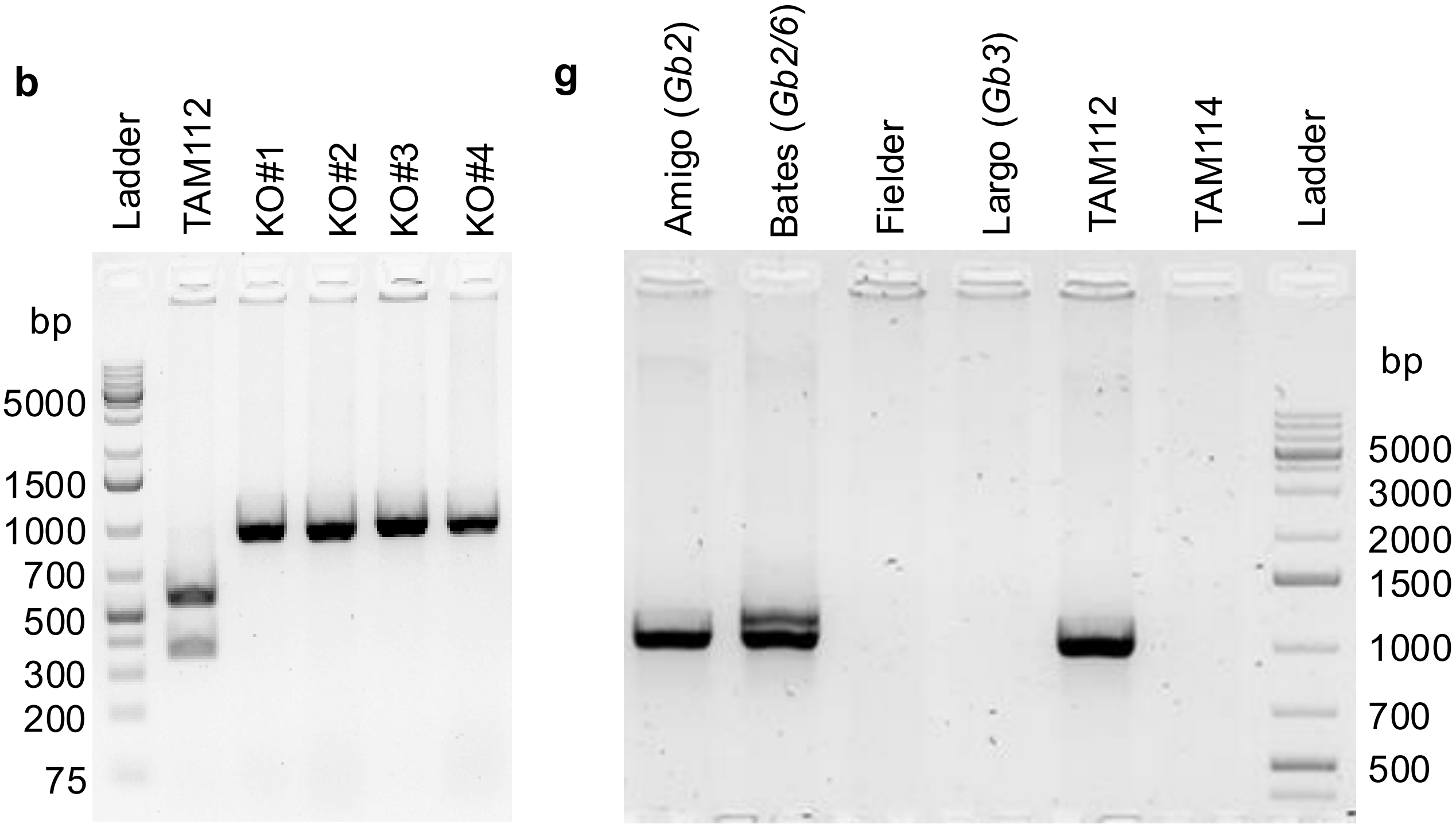

Supplement: Supplementary file 7 [file 41588_2026_2754_MOESM9_ESM.pdf]
